# Measurement Invariant but Non-Normal Treatment Responses in Guided Internet Psychotherapies for Depressive and Generalized Anxiety Disorders

**DOI:** 10.1177/10731911211062500

**Published:** 2021-12-14

**Authors:** Tom H. Rosenström, Ville Ritola, Suoma Saarni, Grigori Joffe, Jan-Henry Stenberg

**Affiliations:** 1 University of Helsinki, Finland; 2 Helsinki University Hospital, Finland

**Keywords:** longitudinal measurement invariance, response shifts, equivalence testing, unipolar trait theory, mood disorders, internet-delivered cognitive behavioral therapy

## Abstract

Assessment of treatment response in psychotherapies can be undermined by lack of longitudinal measurement invariance (LMI) in symptom self-report inventories, by measurement error, and/or by wrong model assumptions. To understand and compare these threats to validity of outcome assessment in psychotherapy research, we studied LMI, sum scores, and Davidian Curve Item Response Theory models in a naturalistic guided internet psychotherapy treatment register of 2,218 generalized anxiety disorder (GAD) patients and 3,922 depressive disorder (DD) patients (aged ≥16 years). Symptoms were repeatedly assessed by Generalized Anxiety Disorder Assessment-7 (GAD-7) or Beck Depression Inventory. The symptom self-reports adhered to LMI under equivalence testing, suggesting sum scores are reasonable proxies for disorder status. However, the standard LMI assumption of normally distributed latent factors did not hold and inflated treatment response estimates by 0.2 to 0.3 standard deviation units compared with sum scores. Further methodological research on non-normally distributed latent constructs holds promise in advancing LMI and mental health assessment.

Dialogue between the fields of psychometrics and clinical psychology might be facilitated by better model scrutiny in a well-focused and pertinent context. Close dialogue is desirable as performance of global model comparison and fit criteria can be context-dependent and clinically unrealistic models may nevertheless attain good statistical fit (e.g., Dziak et al., 2020; Singh, 2009; Yuan et al., 2016). This article therefore investigates and discusses the psychometric advance of *longitudinal measurement invariance* (LMI) model in its important application: as a device to advance measurement and understanding of change processes in mental health interventions. We focus on much-studied public health priorities, depressive disorder (DD) and generalized anxiety disorder (GAD), but similar arguments may apply to many other disorder concepts once their assessment develops (Markon, 2021; Rosenström et al., 2019; Whiteford et al., 2013).

Decline in a sum score of self-reported symptoms is a frequently used measure of treatment success in mental-health interventions. Within psychometric literature, LMI ensures that the self-report inventory in question assesses the same psychological construct before, during, and after the treatment (Liu et al., 2017). The concept of LMI is especially pertinent to psychotherapeutic treatments, where a violation of LMI may be interpreted as a *response shift* in how a client makes sense of a questionnaire item (Fokkema et al., 2013; McLeod, 2001; Murray et al., 2020). Response shifts are systematic changes in some items that occur independently of changes in the measured construct and they can have both pragmatic and theoretical consequences that make LMI techniques potentially valuable psychometric tools.

A response shift, such as a changed perception of a symptom, can be a goal of specific therapeutic techniques, whereas it is undesirable when comparing the overall benefit from different therapeutic approaches using sums of symptoms. Under an LMI violation, one could end up considering two treatments exchangeable because of their equally large sum score declines, when they in fact targeted entirely distinct symptoms that just happened to contribute similarly to the total scores. Such a mistaken “Dodo bird effect” would represent a missed opportunity for treatment personalization and improvement (Wampold & Imel, 2015, p. 33). In the parlance of LMI literature, an above kind of a response shift could be detected as a violation of “threshold invariance” (Liu et al., 2017; technical definitions in below also). But also increases in factor loadings due to the patients learning to better evaluate their symptoms would be considered a response shift while technically corresponding to the concepts of violated “weak,” “metric,” and (for ordinal data) “loadings” invariance (Fokkema et al., 2013; Liu et al., 2017). Thus, the LMI status of the data can have pragmatic implications and it is broadly relevant to the concept of response shifts during psychotherapy. Although we acknowledge that “response shift” can take even more general meanings in the literature (McLeod, 2001), for the purposes of this article, we operationalize it as an LMI violation. In what comes to many technical terms within the LMI literature, we will adhere to the terminology Liu et al. (2017) introduced for ordinal data.

Besides pragmatic treatment-related implications, possible violations of LMI could also contradict the scientific theory that the questionnaire items reflect a temporally stable latent trait construct. Violations of LMI have been reported for depressive symptoms over the course of treatment and these findings have been used as arguments for a general need to revise theory on psychopathology to reflect more the variation in specific symptoms, or networks of symptoms (Contreras et al., 2019; Fokkema et al., 2013; Fried et al., 2016). Perhaps it was a wrong idea to begin with that a simple sum of questionnaire items (or symptoms) could be used to measure and/or define depression? (e.g., Borsboom, 2017; Fried, 2015). In contrast to the earlier findings of LMI violations, more recent works have found LMI to hold for symptom assessments during psychotherapies for depression and generalized anxiety (e.g., Guo et al., 2017; Stochl et al., 2020), arguing that symptom-sum scores can be safely used as measures of treatment response (Stochl et al., 2020). Furthermore, the latent traits (latent factors) of formal LMI models might reflect “true” treatment responses even better than the sum scores due to the ability of LMI models to account for partial measurement invariance (Murray et al., 2020) and to efficiently factor out of measurement errors from the assumed latent trait (K. Bollen & Lennox, 1991).

One could say the LMI models were built to test the latent factor assumption, but a latent factor behind the symptom (or item) correlations is not the only theoretical assumption in typical LMI models. As is typical for structural equation models, the LMI models applied thus far have also assumed a priori that the latent trait in question is normally distributed in the population. Also the individual ordinal symptom items are frequently assumed to reflect a normally distributed liability (Liu et al., 2017; Verhulst & Neale, 2021). That is, the LMI modeling typically relies on normality assumptions as acceptable approximations rather than as testable hypotheses. On one hand, these assumptions are consistent with behavioral geneticists’ view of mental disorders as complex quantitative traits and thereby justifiable (Falconer, 1965; Plomin et al., 2009). On the other hand, many have argued that symmetric distributions are unnatural for symptoms that appear to have an absolute lower bound, which is the lack of the symptom or disorder (Lucke, 2015; Magnus & Liu, 2018; Reise & Rodriguez, 2016).

Our reading of the above literature indicated the following questions remain at least partly open. Does LMI hold for common instruments for measuring depression and anxiety, and why the previous findings disagree despite using large and representative samples? Are the risk traits underlying the psychiatric symptom reports well described by normal population distributions, and what practical and logical consequences such a working assumption has in LMI modeling?

Here, we tackled the above broad issues in the clinical context of online psychotherapy. Guided internet psychotherapies for DD and GAD show treatment effects comparable with traditional face-to-face psychotherapies (Andrews et al., 2018). However, large internet-psychotherapy registers may be particularly well suited to evaluating LMI. At least in our case, the online environment is very structured and all the patients go through mostly the same content. This should facilitate detection of possible response shifts compared with face-to-face psychotherapy registers, where the therapists might use more variable treatment techniques, potentially diluting response shifts due to specific therapy contents. We aimed to (1a) analyze whether treatment response self-reports in internet psychotherapies for DD and GAD adhere to LMI or not, and (1b) whether it is possible to simultaneously entertain both of the apparently conflicting perspectives? Then, we (2a) tested distributional assumptions behind LMI modeling and (2b) investigated their consequences with respect to commonly used sum scores and clinical intuitions.

## Method

### Data

We used questionnaire data from internet-delivered cognitive behavioral therapies (iCBT) for GAD (*n* = 2,218 patients) and DD (*n* = 3,922), prescribed by medical doctors for patients ≥16 years and delivered at HUS Helsinki University Hospital between 2013 and 2020. HUS is the primary provider of specialized public health care services for the 26 municipalities in the region of Uusimaa at Southern Finland (a population 1.7 million), but it has effectively delivered online psychotherapies for the entire country (a population of 5.5 million). This is because HUS has been the only public provider of Finnish language internet psychotherapies and the citizens of Finland can use specialized public health care services from any municipality. We accessed the patient data registry with approvals of Ethics Committee of HUS and institutional authorities. Based on the above details, we can assume the data predominantly represent Baltic Finnic ethnic group native to Finland, although our research register lacked precise ethnicity. We used the entire register available to us and thus we report how we determined our sample size, all data exclusions, all manipulations, and all measures in the study. The data are accessible via HUS Helsinki University Hospital with appropriate research permissions and other materials from Online Supplement, or from the first author upon reasonable request.

The inclusion criteria of the HUS-iCBT for GAD psychotherapy were a GAD diagnosis (World Health Organization [WHO], 2019), an email address, and ability to identify to secure online environment (e.g., online banking ID). The exclusion criteria were acute danger of suicide or self-harm, acute psychotic disorder, serious personality disorder, or a neurological disorder or syndrome that markedly reduces cognitive functioning. The DD psychotherapy targeted mostly mild and moderate DDs (WHO, 2019) and otherwise applied the same exclusion criteria as for the GAD therapy. The HUS-iCBT programs include multiple sessions with different topics, including contents like psychoeducation on iCBT and GAD or DD, goal definition, behavioral activation, relaxation training, cognitive restructuring, advice on a balanced life, relapse prevention, and homework. The patients are encouraged but not required to progress one session per week and they get support from an internet therapist throughout the serially progressing program.

The guided treatments involved 13 and eight weekly sessions for GAD and DD, respectively, with regular contact to a trained internet therapist. The last sessions were 3-month follow-ups. The seven-item Generalized Anxiety Disorder Assessment-7 (GAD-7) inventory was administered at each GAD-therapy session (Spitzer et al., 2006), whereas Beck Depression Inventory (BDI) was administered at Sessions 1, 3, 7, and 8 of the DD therapy (Beck et al., 1961). The HUS-iCBT version differed from the original BDI in that the Item 20 on somatic worries was excluded, leaving altogether 20-item BDI scale. More specifically, the item seems to have been lost from the database rather than intentionally left out. GAD-7 has been developed for screening and follow-up of GAD, especially in primary care context, whereas BDI screens for a broad variety of depressive symptoms. GAD items target symptom frequency within past 2 weeks (not at all, several days, more than half of the days, or nearly every day) and BDI items target verbally expressed ordinal severity levels of symptoms. Both GAD-7 and BDI use 4-point ordinal response scales ranging from no symptom to frequent or severe symptom and both are typically used by summing individual item scores to a total score.

### Statistical Approach

**Figure 1.**
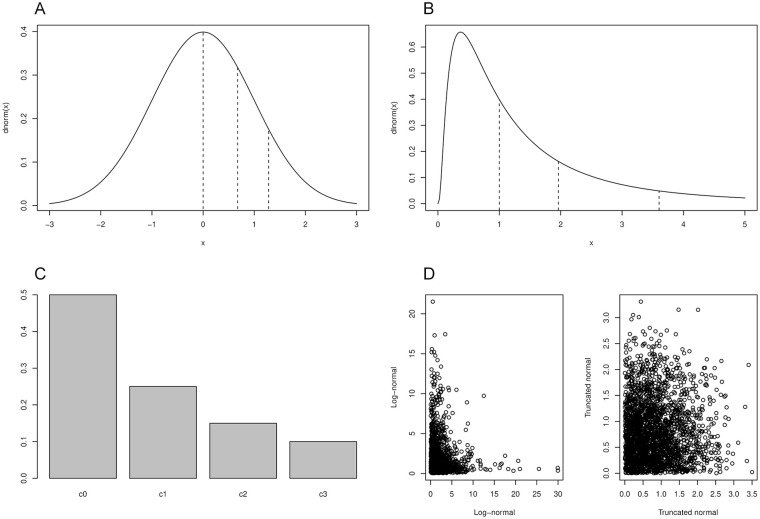
An illustration of liability-threshold modeling for ordinal-valued data. (A) Our inventories ask patients to rate their symptom as being present “not at all” (category 0; c0), “several days” (c1), “more than half of the days” (c2), or “nearly every day” (c3; gad-7), or ask them, for example, whether they “do not feel sad” (c0), “feel sad” (c1), “are sad all the time and can’t snap out of it” (c2), or “are so sad and unhappy that they can’t stand it” (c3; bdi). The underlying variable of interest (e.g., Risk, time, or sadness severity) presumably has a continuous-valued population distribution but is assessed with only four ordered categories. Assuming a normally distributed “liability” continuum, we can estimate threshold values (dashed lines) between which the population at risk lies per ordinal category, thus establishing a link between a continuous-valued data model and the ordinal-valued observations. This link model is called “liability-threshold model,” or sometimes “cumulative model.” Most common choice by far is a normally distributed model of liability, which yields a bipolar liability trait without a finite minimum or maximum value. (B) A log-normally distributed liability would be an example of a unipolar liability trait that is bound below by an absolute minimum value (e.g., No symptom at any time). (C) Liability-threshold models of the panels a and b yield the exact same observed ordinal-valued category-endorsement probabilities despite representing very different underlying models for liability. (D) Log-normal liability would, however, imply more sparse multiway tables than normal liability: simulating values from two log-normal versus (truncated) normal distributions makes this evident. Thus, the choice of underlying normality is not merely a technical one but also partly empirically accessible. *Note.* Gad-7 = generalized anxiety disorder assessment-7; bdi = beck depression inventory.

As both GAD-7 and BDI are typically used as simple sum scores when evaluating treatment efficacy, LMI and lack of response biases should ideally be demonstrated for unidimensional models. Combined with reasonable model fits, this argument led us to concentrate on unidimensional models. Investigations of LMI have traditionally involved conducting a series of statistical tests on increasingly strictly constrained longitudinal confirmatory factor analysis models (Liu et al., 2017). We conducted these tests too, with the typical treatment for ordinal-valued questionnaire items. That is, we used liability-threshold models for the individual ordinal-valued questionnaire items when considering them as parts of structural equation models for LMI (Falconer, 1965; Liu et al., 2017; Verhulst & Neale, 2021). Figure 1A and 1B illustrates how thresholds (dashed lines) on a normal or *non-normal* “liability” can be estimated to map liability-density regions to ordinal-valued item category frequencies of Figure 1C. The normal distribution is a convenient choice that, in structural equation modeling, implies normally distributed latent factors for GAD and BDI. We too used this standard assumption for LMI modeling, implementing the models with lavaan R package version 0.6-7 and weighted least squares mean- and variance-adjusted (“WLSMV”) estimation (Liu et al., 2017; Rosseel, 2012). We set the scales of latent traits so that Session 1 patient-population mean was 0 and variance 1 (along Murray et al., 2020), thus allowing interpretation of estimated means for subsequent sessions as changes in the units of Session 1 standard deviations (see Online Supplement for more comprehensive description of model structures).

First, we evaluated *configural* LMI by assessing whether the same broad factor structure applied over all measurement waves (all therapy sessions), explaining the cross-session cross-item covariance too. In practice, configural LMI is established when the model fits to the data. Configural LMI ensures that response shifts cannot alter the number of latent factors, but it does not yet ensure that the factor loadings stay invariant across sessions. Second, we evaluated *loading* LMI by testing if the fit of a model that fixes factor loadings equal across all therapy sessions differed from that of the configural invariance model. If not, the items can be considered equally sensitive to the latent factor across the therapy sessions. Third, we evaluated *threshold* LMI by testing whether the ordinal item thresholds can be fixed equal across time without deteriorating model fit. Threshold LMI is required to eliminate mean-level changes in items that are not explained by changes in the latent factor. However, it only fixes thresholds relative to each other but not their overall scale. Finally, by further fixing all unique item-liability variances to 1, we tested *unique factor* LMI: If the constraint does not significantly reduce the model fit, then the scales of the thresholds are considered time-invariant and changes in average ordinal responses are fully attributable to changes in the latent constructs. If unique factor LMI holds, we can conclude that there is no (statistical) evidence for response shifts at any level of the LMI model.

Although full LMI is established by the unique factor LMI model, testing the full model sequence is useful for detecting the structural location of potential LMI violations. Testing of the full LMI model sequence is limited by the inevitable need to fix a small number of parameters for model identification, however (e.g., Liu et al., 2017, and our Online Supplement). It is also worth noting that full LMI permits longitudinal changes in the latent factor’s (disorder trait’s) population variance, but a model with such changes is statistically equivalent to another structural equation model having fixed latent factor variance and LMI violations (Muthén, 1998).

Standard null-hypothesis significance testing has been the recommended compromise for the relatively complex LMI modeling of ordinal-valued data (Liu et al., 2017), previously rejecting LMI for measures of depression (Fried et al., 2016). However, it is not necessarily well suited to the LMI-modeling task that seeks to “confirm the null” (the LMI) rather than reject it (Counsell et al., 2020; Yuan & Chan, 2016). In the classic approach that tests a null hypothesis of perfect fit, growing sample sizes lead smaller and smaller violations to exceed statistical significance, rejecting LMI even when the absolute model fit remains good. Conversely, inability to reject the null does not always confirm good model fit as sample size could be insufficient. Therefore, we also tested whether we could reject a null hypothesis that the model fit is poor (instead of the typical null hypothesis of perfect fit), thereby providing support for the alternative hypothesis of acceptable fit. This is called chi-square equivalence testing as opposed to the traditional chi-square difference testing (Counsell et al., 2020). In this case, a *p*-value <.05 indicates no LMI violation (a successful rejection of LMI violations). We used population root mean square error of approximation (RMSEA) of 0.08 with previously published equiv_chi R function (Counsell et al., 2020). To better relate our findings to extant literature, we reported both chi-square difference and equivalence tests. In addition, we report the fit indices of RMSEA and comparative fit index (CFI) that are often used, although problematic in context of ordinal-valued items (Cheung & Rensvold, 2002; Counsell et al., 2020; Liu et al., 2017; Shi & Maydeu-Olivares, 2020). Especially, a CFI decrease of 0.01 or less has been a frequently used effect size–based indicator of LMI (Cheung & Rensvold, 2002; de Jonge et al., 2018; Gluschkoff et al., 2019). Among fit indexes, standardized root mean square residual (SRMR) has been claimed less susceptible to ambiguity from ordinal-data context and is also reported (Shi & Maydeu-Olivares, 2020).

Both structural equation modeling and item response theory (IRT) models typically assume a latent trait θ that has a normal distribution (Liu et al., 2017; Reise & Rodriguez, 2016). But could, for example, the unobservable anxiety-disorder trait behind the GAD-7 questions vary from no problem to a serious problem (0 ≤ θ < ∞) rather than from very “not anxiety prone” to seriously anxious (−∞ < θ < ∞)? Single ordinal-valued item is insufficient information for inferences on underlying latent-trait distributions (e.g., latent distributions of Figure 1A vs. Figure 1B cannot be distinguished from data of Figure 1C). However, joint distributions of multiple ordinal-valued items readily distinguish between normal and non-normal underlying variations, even when assuming only “tips of the iceberg” (risk values) of the population variation are captured by the items (cf. Figure 1D). This fact is utilized by the chi-square test of pairwise normality of symptom liabilities from two-dimensional contingency tables that we used, as implemented in the polycor package version 4.0.3 for R (Olsson, 1979). Similarly, as the latent liability distribution for given ordinal item cannot be simply inferred from its ordinal category endorsement frequencies, the latent disorder trait (latent factor) distribution cannot be simply inferred from sums of ordinal symptom scores. Using a score test of zero-inflation (“score” also refers to derivative of log-likelihood), we verified that LMI model can accommodate simple zero-inflation in symptom counts (Magnus & Liu, 2018; van den Broek, 1995) and only then strived to approximate the shape of the true latent-disorder trait from the multidimensional item data using Davidian curve IRT (Woods, 2015), as implemented in the mirt R package version 1.33.2 (Chalmers, 2012). Whereas structural equation models typically assume latent traits to have an underlying normal population distribution despite the very different observed item category distributions, Davidian curve estimation strives to *estimate* the shape of population distribution from the non-normal item category data. While this semi-parametric methodology has not been extended to full LMI modeling, it nevertheless allows us to evaluate typical distributional assumptions in a time point-wise manner using a flexible spline estimate of latent-trait distribution, introduced to later versions of mirt package. For Davidian curves, mirt implements the maximum marginal likelihood expectation maximization methodology of Woods and Lin (2009). We selected the specific spline approximation from orders 3 to 7 using the recommended Hannan–Quinn information criterion (Woods, 2015). Our Online Supplement further illustrates with simulated data that the method is robust to simple range restriction of ordinal data.

Imputation of missing data is often desirable when significant treatment dropout is observed. However, dropout from online psychotherapies may not adhere to the missing-at-random (MAR) assumption made by most standard solutions for missing data imputation (Arndt et al., 2020). In our Online Supplement, we diagnose results from typical multiple imputation techniques, showing they led to unreliable and biased imputations (Honaker et al., 2011; van Buuren & Groothuis-Oudshoorn, 2011). Although methods to handle non-MAR data have been investigated (e.g., Du et al., 2021), we are not aware of research in ordinal-valued LMI context. And irrespective of the MAR status, the task of imputing ordinal-valued data for LMI testing apparently remains unexplored in the methodological literature (Liu & Sriutaisuk, 2021). Thus, proper imputation techniques for LMI in context of iCBT remain an important and difficult future challenge for the field.

### Results

Our data included 2,218 GAD patients and 3,922 DD patients at Session 1 of the respective internet psychotherapies. GAD-7 was requested in each session from 1 to 13 of the GAD therapy, whereas BDI only in Sessions 1, 3, 7, and 8 of the DD therapy. The available sample shrank by session. Second session of the GAD psychotherapy retained 91% of patients, third session 83%, and so on, until the last actual Session 12 that retained 44%. Only 10% did the follow-up Session 13. In DD psychotherapy, 72% continued to Session 3 and 46% to the last Session 7, with 11% completing the follow-up Session 8. Altogether, we had 17,393 measurement instances on GAD patients and 8,994 observations of DD patients. Table 1 reports some basic sample characteristics for these data.

**Table 1.** Basic Sample Characteristics.

	GAD-7 (*n* = 2,218)^a^			BDI (*n* = 3,922)^b^		
Variable	*M* or %	*SD*	Range	*M* or %	*SD*	Range
Female (%)	77.05	—	—	70.49	—	—
Age (years)	33.69	11.91	15–82	35.86	12.28	16–87
Number of assessments^c^	7.74	4.27	1–12	2.18	0.84	1–3
Days between consecutive assessments^c^	21.93	19.02	1–313	56.14	59.04	4–1490
Days between first and last assessments^c^	138.29	96.90	1–954	92.79	97.09	4–1490

*Note.* GAD-7 = Generalized Anxiety Disorder Assessment-7; BDI = Beck Depression Inventory.

a GAD-7 in online psychotherapy for GAD. ^b^ BDI in online psychotherapy for depressive disorders. ^c^ The 3-month follow-up sessions, session not including symptom reporting, and sessions missing symptom reports were excluded from the calculations (a missing value can occur only in uncompleted or interrupted therapy).

In a logistic regression analysis, Session 1 GAD-7 sum score (odds ratio [OR] of 0.996 per standard deviation, *p* = .570) or female gender (OR = 0.992, *p* = .602) did not predict missingness of later GAD-7 data (i.e., dropping out), but age did weakly predict drop out from the GAD therapy (OR = 0.967 per 1 *SD* increase, or per every 11.9 years, *p* < .001). In DD therapy, age or Session 1 BDI score did not predict drop out (OR = 0.994 and 1.035, with *p* = .322 and .173, respectively), but female gender did weakly predict drop out (OR = 0.973, *p* = .031). Although it might be tempting to use these weak associations to inform an imputation analysis, we avoided that because our supplementary online analyses and the above remarks (see “Method” section) revealed number of obstacles to imputation of LMI models.

### LMI

Our unidimensional factor models had an acceptable fit to data (Table 2). All hypotheses of perfect LMI were rejected but so were all hypotheses of LMI models fitting badly, suggesting that LMI was a reasonable approximation (Table 2). That is, our data sets were sufficiently large to reject perfect model fits requested by the classic LMI approach, but in absolute sense, the LMI models nevertheless fit to data well enough (i.e., the hypotheses of nonequivalence between the data and LMI were rejected, and CFI decreases were < 0.01 in model sequences). Given these findings of acceptable fit, unidimensional scores, including sum scores, would typically be promoted as psychometrically valid procedures. However, this traditional approach simply assumes underlying normal distributions for item liabilities and latent factors instead of testing it.

**Table 2:** LMI Model Fits and Tests for GAD-7 and BDI Assessment During Internet Psychotherapies.

Model	SRMR	RMSEA	CFI	∆(χ^2^)	∆(*df*)	P_MI_	P_ME_
GAD-7							
Configural LMI	0.0425	0.0229	0.9727	—	—	—	<.001
Loadings LMI	0.0425	0.0228	0.9724	178.77	72	<.001	<.001
Threshold LMI	0.0426	0.0225	0.9718	338.47	156	<.001	<.001
Uniform factor LMI	0.0453	0.0210	0.9749	184.85	84	<.001	<.001
BDI							
Configural LMI	0.0663	0.0275	0.9336	—	—	—	<.001
Loadings LMI	0.0664	0.0277	0.9318	489.29	57	<.001	<.001
Threshold LMI	0.0666	0.0270	0.9324	358.31	116	<.001	<.001
Uniform factor LMI	0.0701	0.0254	0.9391	190.35	60	<.001	<.001

*Note.* LMI was tested via a sequence of increasingly strictly constrained SEM. Absolute SEM fit was assessed with SRMR (<0.08 typically indicates relatively good fit), RMSEA (<0.06 for relatively good fit), and CFI (>0.95 for good fit; changes <0.01 typically not considered LMI-violations). Symbols “∆(χ^2^)” and “∆(*df*)” refer to change in chi-square statistic and degrees of freedom from LMI model of the immediately above row. “P_MI_” is *p*-value for measurement invariance test (testing null hypothesis of exact LMI) and “P_ME_” *p*-value for measurement equivalence test (testing null hypothesis of nonacceptable LMI model fit). The *p*-values were <.001 also when sample size reflected average response rate instead of number of patients in first session (not shown). LMI = longitudinal measurement invariance; SEM = structural equation model; SRMR = standardized root mean square residual; RMSEA = root mean square error of approximation; CFI = comparative fit index.

### Testing Distributional Assumptions of the Standard LMI Approach to Categorical Data

#### Testing (Pairwise) Normal Item Liabilities

As suggested by Figure 1, the information on a latent factor distribution’s shape must be recovered from more than one ordinal variable. Before trying to estimate non-normal shapes, we investigated whether simple bivariate item–item distributions suggested the required information existed in the data. In Session 1 of the GAD iCBT, 18 of 21 (86%) possible pairwise item–item contingency tables violated the assumption of latent bivariate normal distribution, which is much more than the expected type I error rate of 5%. In the last Session 12, 19 of 21 (90%) pairwise tests rejected the assumed underlying normality. For BDI at Session 1, 188 of 190 (99%) of item pairings violated the latent-normality assumptions, and for the last Session 7, 184 (97%) of item pairings violated normality. These data indicate a violation of LMI model assumptions at the level of pairwise symptom liabilities, despite the LMI model reproducing well the marginal item category endorsements by assumption. They also stress the need to estimate rather than assume latent-trait distribution’s shape.

#### Estimated Latent Trait Distribution

**Figure 2.**
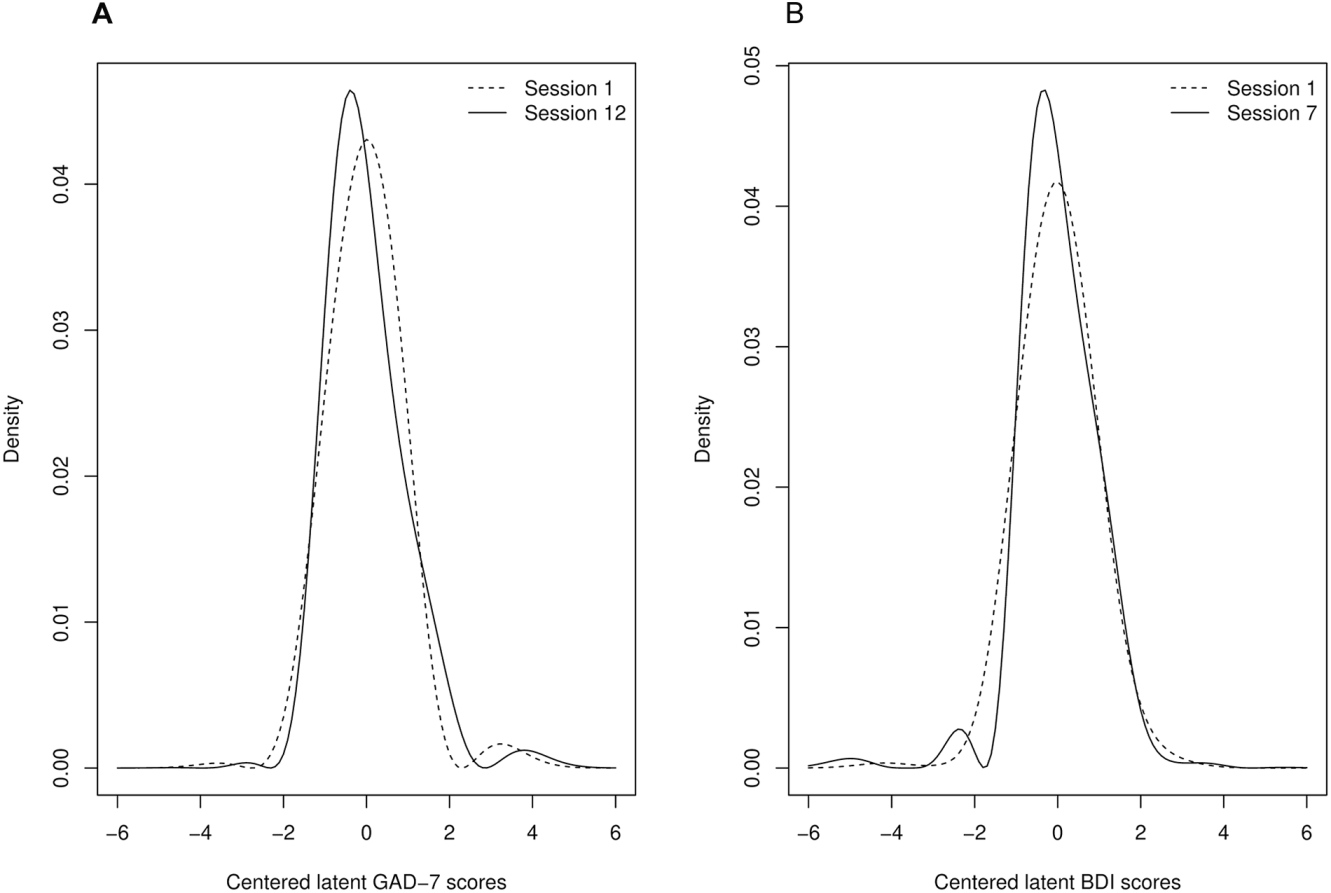
Empiric davidian curve item response theory estimates for the shapes of the latent factor distributions in the first and last online psychotherapy sessions, estimated separately. That is, values are standardized to within session variability and do not capture treatment effects, only distributional shapes, suggesting that latent factor distributions get more skewed as the therapies progress. (A) GAD-7 latent scores in gad internet psychotherapy sessions 1 (dashed Line) and 12 (solid Line). (B) BDI latent scores in depressive disorder internet psychotherapy sessions 1 (dashed line) and 7 (solid line). *Note.* GAD = Generalized Anxiety Disorder Assessment-7; BDI = Beck Depression Inventory.

Davidian curve estimation revealed how symmetrically distributed “true” population latent factor construct at the therapy start changed to skewed distributions at the final sessions (Figure 2). This effect is not present in the LMI models that assume symmetric latent-trait distributions throughout. It is worth stressing that continuous-valued latent liabilities for the ordinal-valued data were modeled and thus this finding is not a simple consequence of a floor effect in the ordinal questionnaire items and sum scores, or of items providing information only on the tail of the underlying latent factor distribution. In our Online Supplement, we show by simulation that Davidian curve estimation correctly recovers a true underlying normal distribution irrespective of ordinal items sampling it evenly or from the right tail only.

For further example, in the first therapy session of the GAD therapy, the rate of unendorsed GAD-7 symptoms could be predicted from a simple Poisson distribution for counts of endorsed (nonzero) symptoms, χ^2^(1) = 1.639, *p* = .200, for a score test, whereas in the last Session 12, this was not the case, χ^2^(1) = 78.259, *p* < .001. Thus, a dramatic change in zero-inflation was observed. However, the fitted uniform factor LMI model predicted very similar levels of zero-inflations for the Sessions 1, χ^2^(1) = 2.587, *p* = .108, and 12, χ^2^(1) = 71.598, *p* < .001, indicating that the model can accommodate to changes in skewness or zero-inflation of the observed ordinal values. But how exactly, and are unintended consequences involved?

### Consequences of LMI Violations and Normality Assumptions for Treatment Effect Estimation

**Figure 3.**
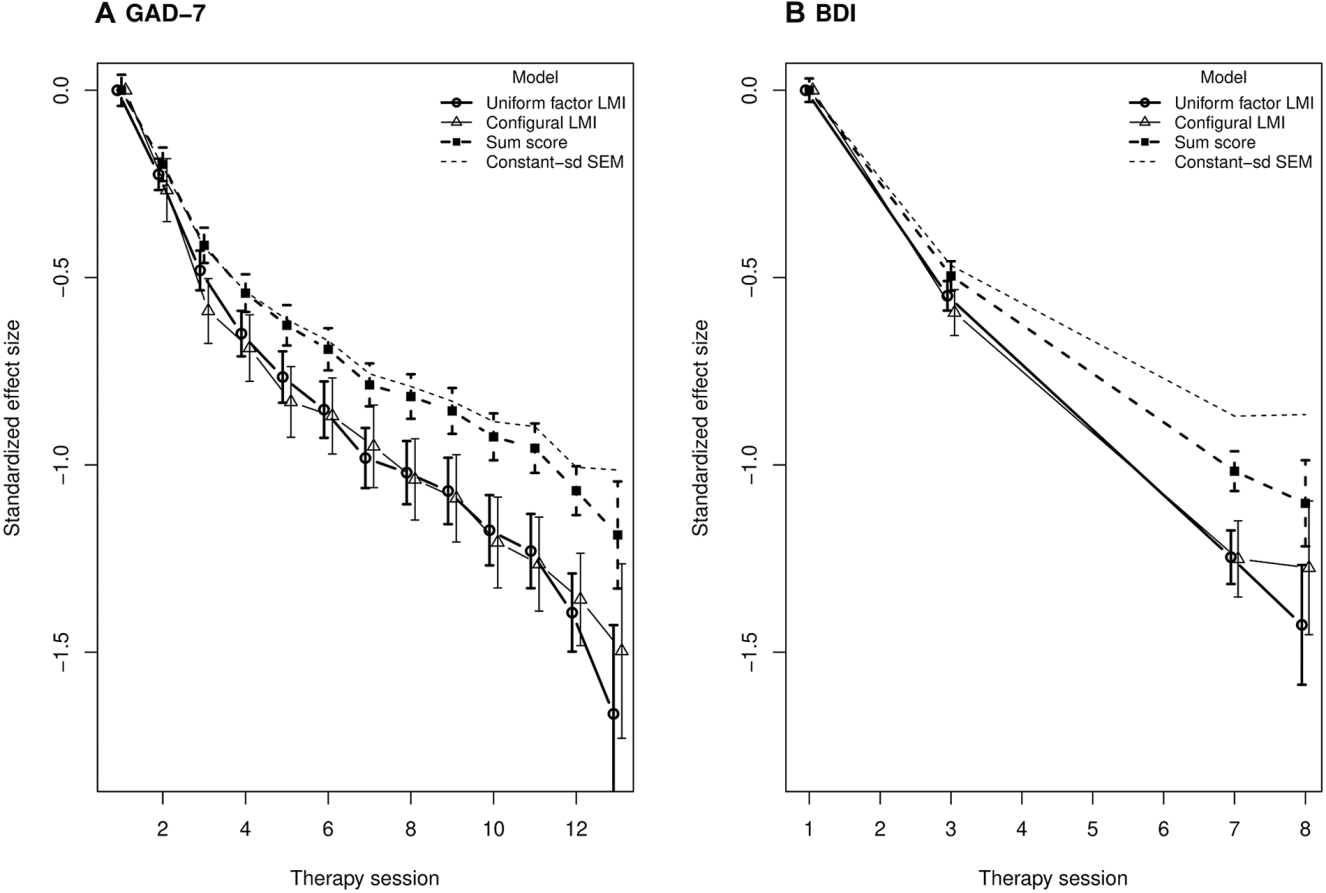
LMI and treatment response in online psychotherapies for GAD and any DD. (A) Results on gad assessment-7 (GAD-7) self-reports. Average latent factor scores for a model of full “uniform factor” LMI (thick, solid line), a model of “configural” lmi only (thin, solid line), and for observed “sum scores” (thick, dashed line) over the course of the online psychotherapy for GAD. Whiskers are for 95% confidence intervals, with effect sizes standardized so that session 1 mean is 0 and session 1 standard deviation is 1. Conclusions regarding efficacy are highly similar across lmi models but different for sum scores. If the other parameters of the uniform factor LMI model were “standardized” to session-specific latent factor standard deviations instead of session 1 standard deviation, the ensuing “constant-SD” SEM yielded treatment responses much closer to the sum scores (thin, dashed line). This model is equivalent to the uniform factor LMI model in fit but lacks the lmi interpretation (i.e., its structural parameters are not invariant across sessions). (B) Same as panel “A” but for bdi in the online psychotherapy for DD patients. *Note.* LMI = longitudinal measurement invariance; GAD-7 = Generalized Anxiety Disorder Assessment-7; DD = depressive disorder; SEM = structural equation model; BDI = Beck Depression Inventory.

In the above section on measurement invariance, violations of LMI were detected using null-hypothesis significance testing, but the equivalence testing and effect size (CFI) viewpoints suggested that these minor violations lacked practical relevance. Indeed, the models with minimal (i.e., configural) versus maximal (i.e., uniform factor) LMI gave virtually identical estimates of treatment response (Figure 3). This supports the idea that the results from equivalence testing (those supporting LMI) are the practically relevant results, whereas the classic chi-square difference testing appeared oversensitive, at least in what comes to treatment response estimation. However, both the LMI models suggested greater treatment response than the sum scores, which are typically used in the clinical practice and research (Figure 3). The LMI model estimates were a 1.39 standard unit’s decrease in GAD symptoms from Sessions 1 to 12 of GAD therapy and a 1.23-unit decrease in DD symptoms from Sessions 1 to 7 of DD therapy. These were 1.30- and 1.21-fold effects compared with those inferred from sum scores (1.07 and 1.02), respectively.

**Figure 4.**
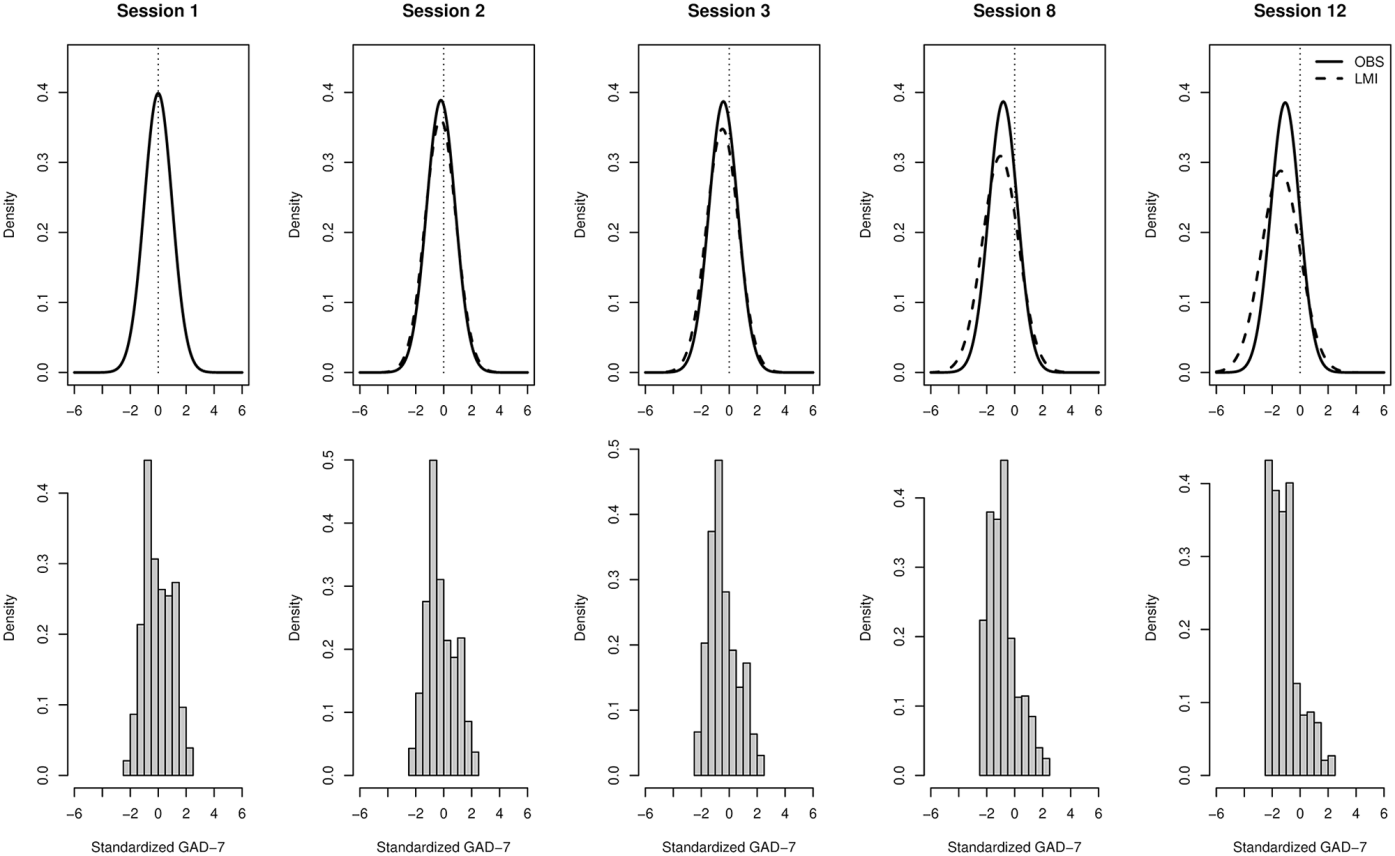
Sum-score model versus LMI model of treatment response in online psychotherapy for GAD. Standardized units of GAD-7 Symptom scores are shown in X-axes and probability/histogram densities in Y-axes. For clarity and brevity, only data from sessions 1, 2, 3, 8, and 12 are shown. Top row: for a normal distribution with mean and variance equal to those of observed sum scores (OBS: solid lines), we see a translation to lower means as the psychotherapy proceeds, but no visible changes in variance. For the uniform factor LMI model (dashed line), we see both decrease in mean and increase in variance as the psychotherapy proceeds. Bottom row: empiric sum score histograms for comparison. Note how an increasing proportion of the latent factor distributions in the top row decline below the lowest values observable from sum scores in the bottom row (below “thresholds”) as the psychotherapy proceeds. The typical LMI modeling assumes the skewed histogram, for example, at session 12 reflects a tail of a flattening and only partially visible normally distributed liability, but it does not investigate genuinely skewed or unipolar liability distributions (cf. Figure 1A vs. 1B). *Note.* LMI = longitudinal measurement invariance; GAD-7 = Generalized Anxiety Disorder Assessment-7.

We wanted to find out why the LMI models suggested greater treatment effects than the sum scores. We observed that the sum-score distribution skewness increased approximately eightfold during the course of the GAD therapy, with variance staying nearly the same, whereas the latent factor skewness stayed the same by the normality assumption in the LMI model, with variance increasing approximately threefold. The LMI model appeared to adjust to increasing zero-inflation or skewness in the ordinal data by increasing latent factor variance *while simultaneously* decreasing latent factor mean relative to sum scores (Figure 4). These effects were linked in the sense that when we fitted the exact same LMI model using an additional constraint of fixed latent variance across sessions, the model produced treatment effect estimates closer to sum scores. This happened at the expense of a worse overall fit to data, however (∆χ^2^ = 290.92, *df* = 12, *p* < .001)—genuinely worse fit, as also the equivalence testing failed to reject unacceptable fit (*F*_ml_ = 0.218, ε_0_ = 0.077, *p* = 1). Finally, it is possible to standardize the latent variance across sessions without decreasing model fit by using a different parameterization instead of additional model constraints (Muthén, 1998): again, this version of the model produced treatment effects closer to the sum score estimates than in the LMI models (see Figure 3). The catch, however, is that LMI no longer holds under the fixed-variance parameterization (e.g., factor loadings vary across sessions). These observations indicate that the underlying normality and the LMI cannot hold simultaneously unless the latent treatment effects and symptom variance increase in a way unparalleled by the observed sum scores. The mismatch between the theoretical model and the sum scores forces a practitioner to decide which one he or she believes in.

**Figure 5.**
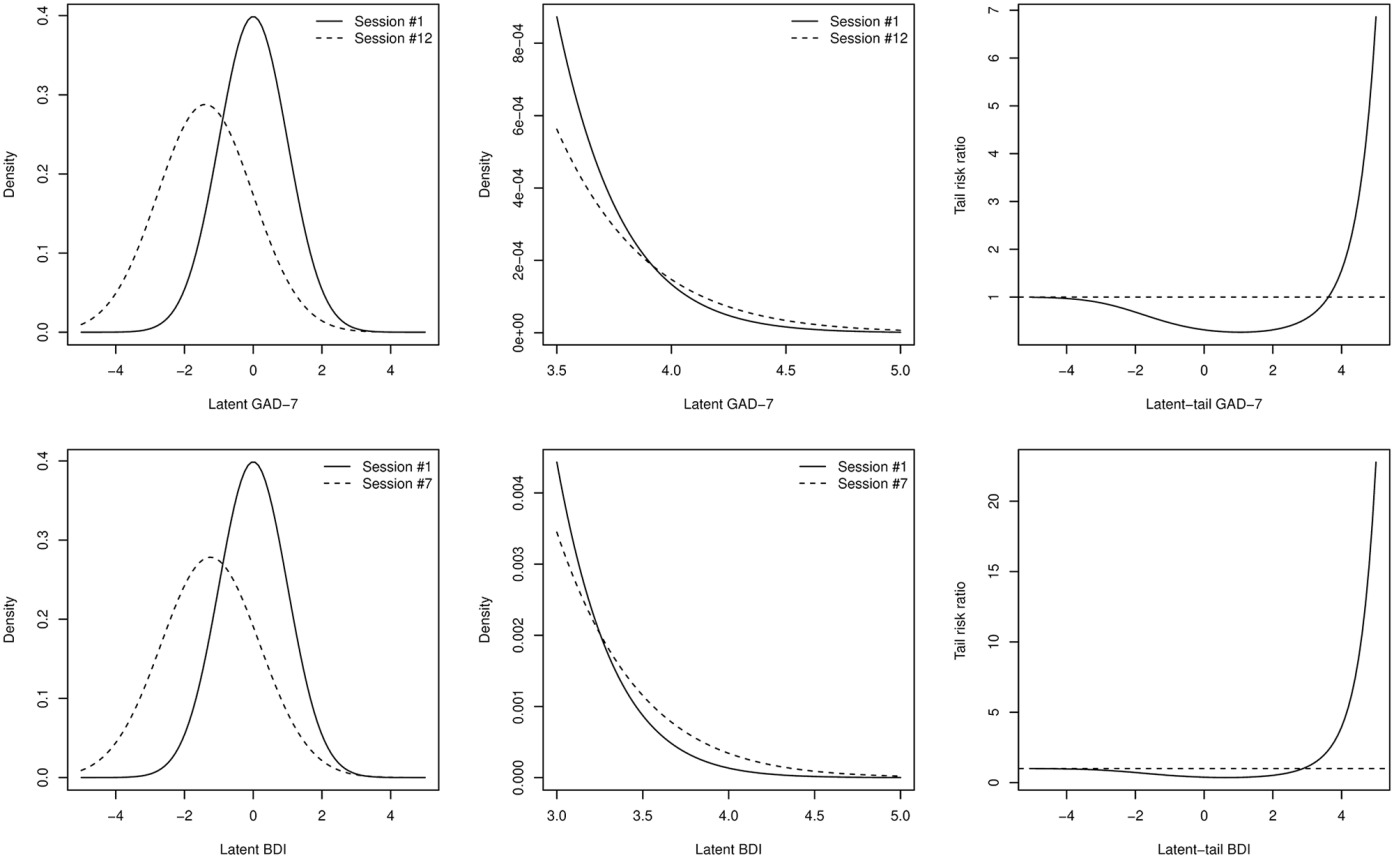
Tail effects in uniform factor LMI model. Top row is for GAD-7 inventory and bottom row for BDI. Left column: model-predicted latent factor distribution in the first (solid line) and last (dashed line) therapy sessions. Middle column: a zoom-in on the right-hand tails of the same distributions. Note how distributions for the traits in the last sessions actually spread probability mass more to the very severe end of the continuum than those in first session. Right column: tail risk ratio plotted against latent trait. The tail risk ratio was defined as probability of higher latent-trait value than X in the last session divided by probability of a value higher than X in the first session, where X refers to X-axis. Note how the risk is mostly below one as expected from the leftmost panels, but explodes at the tails due to the inflection points revealed by the middle panel. *Note.* GAD-7 = Generalized Anxiety Disorder Assessment-7; BDI = Beck Depression Inventory.

Do the normality assumptions have other consequences besides the above-detected ambiguity in the effect size estimates? For another example, we investigated effects of the latent-variance increase by session on top-coded severity. While a cursory look might indicate that the uniform factor LMI model predicted a decrease in the latent-disorder factor across its entire range (Figure 5, left column), this does not hold true for the very highest severity levels (Figure 5, middle column). The LMI model thus implies that probability, or risk, of finding a case more severe than a given factor level is generally lower after the therapeutic intervention than before it, but higher for the most severe levels (Figure 5, right column). This model prediction seems unintuitive, but is difficult to test empirically as it pertains to unobservable quantity of the model and testing would require a very large data set to capture rare events.

## Discussion

When modeling GAD-7 and BDI online questionnaire data on patients undergoing internet psychotherapy treatment at HUS Helsinki University Hospital in Finland, we found (1a) that the self-report data satisfied LMI sufficiently well from the equivalence testing and effect size perspectives. Yet, (1b) it was simultaneously possible to argue for violations of LMI from the chi-square difference testing perspective. Thus, the LMI outcome can differ depending on whether researchers test a null hypothesis of perfect fit or a null hypothesis of unacceptable fit. The possible violations of LMI were not consequential for the estimated size of treatment response, however. Instead, we found (2a) evidence that a priori assumed shapes of latent factor and item-liability distributions in the LMI models did not strictly hold true when tested for and (2b) this fact likely did play a role in treatment response estimation. For example, the use of LMI modeling increased the treatment effect observed for standardized sum score (~1.1 *SD*) by an additional ~0.3 standard deviation units in the GAD internet psychotherapy. Paradoxically, the LMI model also predicted a simultaneous total symptom decrease and an increased prevalence of most severe cases after treatment—a prediction that was not empirically testable presently.

Key aims of this article were to investigate LMI in clinically relevant topics using state-of-art methodology *and* to identify where the “state of the art” needs more methodological work to increase the clinical relevance of the LMI methodology. Currently, methods such as structural equation modeling and Davidian curve IRT have complementary strengths applicable to different contexts but we are not aware of work that combines them in ways applicable to large clinical data we analyzed. Next, we discuss the meaning of our findings and argue that further work toward understanding latent liability distributions may be important for advancing mental health research.

An acceptable level of LMI has two kinds of important implications for outcome monitoring in psychotherapy. First, LMI ensures that, for example, response shifts (changes in reporting) do not bias our estimate of treatment response, thereby ensuring that we do not interpret treatment success (or failure) from patients merely learning to interpret certain questionnaire items in a different way. Second, LMI increases our trust in having a correct conceptual model for the underlying disorder in that the questionnaire items reflect an underlying GAD or DD factor. Given the apparent importance of LMI, it may seem disturbing that findings on LMI during DD and/or GAD treatments have been mixed, with some studies concluding a clear lack of LMI (Fokkema et al., 2013; Fried et al., 2016) and others supporting LMI (e.g., Brunet et al., 2014; Stochl et al., 2020). Here, we showed that the same data can support these apparently conflicting views on LMI depending on the researchers’ adherence to traditional chi-square difference testing *versus* chi-square equivalence testing (Counsell et al., 2020; Yuan et al., 2016).

Authors of an influential didactic article recommended favoring traditional chi-square testing over fit indexes in context of LMI (Liu et al., 2017), but other recent research promotes chi-square equivalence testing over the traditional testing (Counsell et al., 2020; Yuan & Chan, 2016; Yuan et al., 2016). Besides the results reported here, also the previously reported finding on lack of LMI by Fried et al. (2016) can be re-interpreted as LMI holding from the equivalence testing point of view.^1^ Overall, we conclude that LMI seems to approximately hold when measuring symptoms of depression and anxiety during treatments with GAD-7, BDI, and many other common inventories. Furthermore, whatever small violations remained here had negligible influence on treatment effect estimation.

Instead of the linear structural constraints of the canonical LMI testing sequence, we would like to draw research focus on the factor model itself and its assumptions. While some research exists on performance of chi-square difference testing under violations of normality assumptions, violations of *latent* normality assumptions appear largely an unexplored topic (Pavlov et al., 2020). Hence, although taking the equivalence testing viewpoint may make LMI a more consistent finding than the available literature indicates, we need further research on how the methods perform when we know that the underlying distributional assumptions are not met. Interestingly, Finch et al. (2018) found group-wise measurement-invariance methodology to cope well with continuous-valued data underlined by a non-normal latent factor, suggesting that LMI testing might be more robust to latent factor non-normality than inferences about the factor itself. Presently and until better-suited ordinal-data models emerge, we consider LMI a good working hypothesis for clinical research, agreeing with Stochl et al. (2020) in that the use of sum scores appears acceptable. Although this article was primarily about estimating latent constructs whose distribution has little to do with raw sum-score distributions or even derived factor-score distributions, construct-level measurement invariance typically implies that of factor and sum scores (see, for example, our supplementary material or K. A. Bollen, 1989). But what about the supposed superiority of latent factors over sum scores?

We found that estimates based on latent factors differed from those based on sum scores, but which units a practitioner should read: the latent and supposedly true factor change with its associated larger treatment effect or the change in the sum of the ordinals (sum scores) with the smaller treatment effect. Sum scores are linear composites that reduce but do not eliminate measurement error. Therefore, they lead to biased estimates of treatment effects, whereas structural equations can tease apart measurement noise from true population-level effects (K. Bollen & Lennox, 1991). Thus, it would be tempting to conclude that our LMI model estimates of treatment effect sizes exceeded the estimates based on sum scores because they more accurately reflected true underlying treatment effects. However, we provided compelling evidence that it was not measurement noise but the normal-distribution assumption for latent factors that made the difference of ~0.3 standard units in treatment response estimation. Hence, the question of what units should be used is not self-evident but rather hinges on the question of whether the increasing latent-trait variance is a property of the model or corresponds to some psychological reality? Before addressing this more difficult questions, we start by addressing the simpler question, “Are small differences in treatment response estimates practically relevant?”

Should one be worried about a difference of 0.3 standard units in different statistical models if they provide other complementary benefits in some contexts? We think, yes. When standardizing effect sizes to baseline (or Session 1) standard deviation like here, our treatment effect in terms of BDI sum scores exceeded average previous findings by 0.39 standard units (1.07 vs. 0.68; Karyotaki et al., 2018).^2^ Add the 0.3 units from the LMI model assumptions, and we could have claimed a double effect size compared with an average internet psychotherapy. The 0.3 units is also an effect size between the conventional “small” (0.2) and “medium” (0.5) sized effects (Cohen, 1988, p. 40). Furthermore, it has been difficult to find active components of psychotherapies, or even theoretical schools, that would supersede other components or schools in treatment efficacy (Cuijpers, Cristea, et al., 2019; Cuijpers, Reijnders, et al., 2019; Wampold & Imel, 2015). Yet, ability to demonstrate superiority of some procedures plays a key role in treatment development. Accurate, theoretically justifiable, and standardized measurement procedures should facilitate our ability to find key elements of psychotherapy via more accurate detection of small effects. The robust small effects can then perhaps be developed to large effects via further research and procedure optimization.

So, which one to use, sum scores or latent traits? Our results indicated that latent normality assumptions typical to LMI and structural equation models did not hold when separately tested for. As all models are wrong and some useful, we nevertheless explored consequences of these assumptions, finding they necessarily translated to latent-trait variance increasing by treatment session, which further implied an unintuitive joint decrease in symptoms and increase in most severe cases by session with treatment. Without any further empirical evidence on such psychological phenomena, we would be inclined to rely on the “non-parametric” sum-score-based treatment response estimates over parametric latent factors at this point. However, it seems a research priority to extend the LMI models to more flexible underlying latent-trait distributions, both for scientific understanding and because sum scores cannot eliminate measurement error to the same extent a correctly identified latent factor can (K. Bollen & Lennox, 1991). Recent psychometric research has begun tackling the issue of latent non-normality but we are not aware of extensions to the context of LMI and ordinal item data (Finch et al., 2018; Lucke, 2015; Magnus & Liu, 2018; Reise & Rodriguez, 2016; Woods, 2015).

Although we expect our findings to generalize well across different contexts, they should be interpreted in the light of the following limitations. Treatment dropout may bias estimates of (intention-to-treat) treatment effects, with both negative and positive biases being feasible (Arndt et al., 2020). Hadjistavropoulos et al. (2014) conducted a small survey on reasons for dropping out from iCBT: They found that more patients did so for the reason that their “primary symptoms had improved” (22.2%) than for the reason of “not benefitting from the program” (3%). When sufficient improvement rather than nonimprovement is the dominant reason for drop out, one would expect underestimated rather than inflated nonimputed treatment effect estimates. Sample attrition from tails of the symptom distribution could also influence shape of underlying distributions, but generally reduces rather than increases variance (see, for example, Johnson et al., 1994, and our online supplement). As the aims of this study pertained different models of measurement rather than a best estimate for known measure, we used all the pairwise complete data but avoided further model-dependent missing data analyses. Literature indicates the missing-at-random assumption typically required in missing-data imputation may not hold for iCBT treatment data (Arndt et al., 2020; Hadjistavropoulos et al., 2014) and our supplementary online analysis indeed revealed failures in two widely used methods. We consider developing methods for modeling nonignorable missingness in ordinal-data LMI models an important future methodological aim in general, but expect that main conclusions of this article will withstand such adjustment efforts. In general, our treatment dropout was at normal levels for guided internet psychotherapies, was much less than typically observed for self-guided treatments, and can be improved via additional guidance (Karyotaki et al., 2015; Pihlaja et al., 2020).

Although GAD-7 and short depression questionnaires tend to fit well to unidimensional one-factor models, longer inventories like BDI typically give rise to a small degree of variance along additional common factors (Fischer et al., 2021; Huang & Chen, 2015; Stochl et al., 2020). Nevertheless, even the longer inventories fit well to one-factor models (Huang & Chen, 2015), and are typically essentially used as such when evaluating treatment effects from simple sum scores of symptoms. Here, we wanted to keep our models as close to this typical use case as possible. This made sense because the exact theoretical constructs for depression and anxiety remain debated and could have distributions other than those found for the latent factors as operationalized here and typically. Finally, we lacked a control group as our analysis was based on a naturalistic treatment register. Although this is not problematic in the sense that this study was not a treatment-efficacy trial, a deeper understanding on latent factor dimension and invariance structures might, of course, be attainable by examining several pertinent patient groups and healthy controls.

The strengths of this study include a large naturalistic treatment register, our clinically oriented investigation of psychometric and LMI modeling research, and methodology robust to range-restriction issues in commonly used Likert-type scales. We conclude that violations of LMI do not appear to undermine the typical use of symptom sum scores in estimation of treatment effects in guided GAD and DD internet psychotherapies, although such implications easily arise from the potentially oversensitive classic chi-square tests frequently used in LMI modeling. Instead, typical distributional assumptions in LMI models and other psychometric models may undermine precise treatment response estimation. Methodological research on non-normally distributed latent constructs in LMI context holds promise in advancing psychotherapy research and mental health assessment in general.

## Supplemental Material

sj-docx-1-asm-10.1177_10731911211062500 – Supplemental material for Measurement Invariant but Non-Normal Treatment Responses in Guided Internet Psychotherapies for Depressive and Generalized Anxiety Disorders Click here for additional data file. Supplemental material, sj-docx-1-asm-10.1177_10731911211062500 for Measurement Invariant but Non-Normal Treatment Responses in Guided Internet Psychotherapies for Depressive and Generalized Anxiety Disorders by Tom H. Rosenström, Ville Ritola, Suoma Saarni, Grigori Joffe and Jan-Henry Stenberg in Assessment

## Notes

1. Fried et al. (2016) reported chi-square difference test parameters *N* = 649, ∆(χ^2^) = 132.52, ∆(*df*) = 70, and *p* < .001 for their greatest longitudinal measurement invariance (LMI) violation pertaining a self-report instrument. Their absolute model fit was good, though (root mean square error of approximation [RMSEA] = 0.03 and comparative fit index [CFI] = 0.96). These parameters imply chi-square equivalence test with parameters *F*_ml_ = 0.205, ε_0_ = 0.448, and *p* < .001 for an acceptable fit of RMSEA = 0.08 (Counsell et al., 2020). Thus, both the hypothesis of perfect fit and the hypothesis of unacceptable fit would get rejected, although Fried et al. (2016) tested only for perfect fit.

2. The effect size 0.62 was given only indirectly in Karyotaki et al. (2018), being inferred from their Table 2 numbers for BDI baseline and posttreatment means and baseline standard deviation in the intervention group.
