# Supplementary material for: Measurement Invariant but Non-Normal Treatment Responses in Guided Internet Psychotherapies for Depressive and Generalized Anxiety Disorders
Source: Assessment. 2021 Dec 14;30(3):618–32. doi: 10.1177/10731911211062500 (PMC9999284; doi:10.1177/10731911211062500)
Supplement: sj-docx-1-asm-10.1177_10731911211062500 – Supplemental material for Measurement Invariant but Non-Normal Treatment Responses in Guided Internet Psychotherapies for Depressive and Generalized Anxiety Disorders [file sj-docx-1-asm-10.1177_10731911211062500.docx]

**SUPPLEMENTARY MATERIAL:**

**Measurement Invariant but Non-Normal Treatment Responses in Guided Internet Psychotherapies for Depressive and Generalized Anxiety Disorders**

Contents

[On imputation strategies 2](#_Toc86582061)

[On the relationship between factor and sum-score invariance 3](#_Toc86582062)

[A simulation example of Davidian curve estimation 4](#_Toc86582063)

[On the structural equation model constraints and scale setting 4](#_Toc86582064)

[On tail attrition and variance 5](#_Toc86582065)

[Supplementary references 6](#_Toc86582066)

[Supplementary figures 8](#_Toc86582067)

[Figure S1. Examples of badly behaving mice chains. 8](#_Toc86582068)

[Figure S2. Observed (blue) and imputed (red) value densities from Amelia II imputations. 9](#_Toc86582069)

[Figure S3. Overimputations for V3T2 of Fig. S2. 10](#_Toc86582070)

[Figure S4. Overimputations for V3T12 of Fig. S2. 11](#_Toc86582071)

[Figure S5. Simulation results for Davidian curve estimation. 12](#_Toc86582072)

[Figure S6. Sketched path diagram for configural invariance model. 13](#_Toc86582073)

[Figure S7. Truncated normal variance 14](#_Toc86582074)

[An R script to test Davidian curve estimation using simulated data 15](#_Toc86582075)

[Printed model output from lavaan R package 16](#_Toc86582076)

[Online Psychotherapy for generalized anxiety disorder 16](#_Toc86582077)

[Online psychotherapy for depressive disorders 40](#_Toc86582078)

# On imputation strategies

To our knowledge, extensive investigations of imputation strategies have been undertaken for group-wise measurement invariance models under ordinal-valued data but not for longitudinal measurement invariance under ordinal-valued data (Liu & Sriutaisuk, 2021). Our data thus contains features not properly tested in simulation studies of imputation strategies: (*i*) ordinal-valued data in longitudinal measurement invariance testing and (*ii*) increasing rate of missingness per study wave combined to (*iii*) increasingly non-normal latent factors (or increasing latent-factor variance). Full information maximum likelihood (FIML) and one from five multiple-imputation based methods showed decent performance for group-wise measurement invariance testing with ordinal-valued data, however (Liu & Sriutaisuk, 2021), and could be expected to do well in longitudinal testing too. Unfortunately, FIML method has limited ability to test unique factor variances, needs more research regarding configural invariance testing (Liu & Sriutaisuk, 2021), and suffers from a computational bottleneck for a large number (≥20) of ordinal variables (Zahery et al., 2017), which we had [84 (i.e., 7×12) for twelve GAD-7 follow ups, and 80 for our BDI version sampled 4 times]. Thus, for this empiric analysis, we were left with the multiple-imputation option, untested in this specific type of imputation task.

We tested two multiple imputation packages, “mice” and “Amelia II” that rely on the missing-at-random (MAR) assumption, paying careful attention to the quality of their imputations (Honaker et al., 2011; van Buuren & Groothuis-Oudshoorn, 2011). Besides the questionnaire items under investigation, we used age and sex for imputation. Using predictive mean matching (or ordered logit regressions), mice chained equations did not readily converge for some items (Figure S1). Potential reasons include increasing missingness by wave, non-MAR missingness, and linearity (or proportional odds) assumptions for the ordinal-valued data.

Deeming the mice imputations unsafe, we tested Amelia II. Amelia II strives to automate the tuning of the imputation model via convenient working assumptions and use of the expectation-maximization with bootstrapping as the imputation algorithm (Honaker et al., 2011). It provides a slightly different set of diagnostic tools for evaluating quality of its imputations. First, by comparing imputed values’ densities (red) to observed values (blue), we noted that imputations lead to systematically lower values compared to observed data (Figure S2). Of course, this could happen both due to bad imputations (i.e., algorithm gone askew) or due to good imputations combined with massively selective missingness (i.e., the algorithm doing exactly what it was intended for). Luckily, Amalia II incorporates a clever technique to distinguish between these fundamentally different alternatives—overimputing.

Overimputing sequentially treats each of the actually observed values *as if they had been missing*, such observed data then providing a ground truth against which one can compare the imputations. Now, we know the imputed values should be close to the observed values they aim to recover, in case the algorithm is working as intended. Plotting all overimputed values and their 90% confidence intervals (CI) against the observed values e.g. for the 3^rd^ GAD-7 item at therapy sessions #2 (Figure S3) and #12 (Figure S4), we observed that imputed values did indeed systematically underrepresent the highest item category and over-represent the lowest category. Proportion of observed values not within the 90% CI of its imputed counterpart (e.g., 11% in session #2) was, in fact, close to the nominal rate of 10%. However, for the lowest (27%) and the highest (18%) item categories the imputations were clearly biased. The near-nominal rate was only an appearance resulting from the many observations (73% of all) in the intermediate categories achieving better than nominal coverage (8%).

Thus, we concluded that it was a difficult task to create reliable imputations for these data—a task that requires primary simulation studies. We speculated reasons for failures of imputation algorithms to pertain to violations of the MAR assumption and/or the imputation models lacking sufficiently general ordinal-data models. Both patients who feel they have recovered sufficiently and patients who do not benefit from the therapy may be more likely to quit doing therapy sessions compared to patients gaining steady benefits (Arndt et al., 2020). This means that the data is not missing at random given the observations, because the missingness depends on the likely GAD-7 (or BDI) values of unobserved sessions. This could violate the MAR assumption needed for imputation. The effects of such violations may get compounded by the fact that none of the imputation techniques allows non-symmetric thresholds for underlying liabilities to endorse item categories (i.e., fully general ordinal values are not supported by mice or Amelia II despite both having partial solutions for ordinal data).

# On the relationship between factor and sum-score invariance

According to Bollen, “[s]ome researchers use factor score estimates in observed variable models (e.g., regression models). The implicit assumption is that the factor score estimates remove the problems with measurement error present in the unadjusted variables. Though using [a factor score] in place of single indicators for latent trait can reduce measurement error, it does not remove it.” (Bollen, 1989, p. 306). We develop this argument further in order to illustrate why demonstrating measurement invariance in factor models also suggest lack of response shifts in the simple sum scores commonly applied in clinics.

The regression method of factor score derivation takes a weighted sum of observed item vector ***x*** to estimate the latent score $\hat{\xi}$ (Bollen, 1989, p. 305). Specifically,

$\hat{\xi}\boldsymbol{=}\hat{\boldsymbol{\Phi}}{\hat{\boldsymbol{\Lambda}}}_{\boldsymbol{x}}{\hat{\boldsymbol{\Sigma}}}^{\boldsymbol{-1}}\boldsymbol{x}$,

where $\hat{\boldsymbol{\Phi}}$, ${\hat{\boldsymbol{\Lambda}}}_{\boldsymbol{x}}$, and ${\hat{\boldsymbol{\Sigma}}}^{\boldsymbol{-1}}$ are fixed matrices determined by the estimated factor-model parameters. Essentially, a factor score estimate is then observed item scores weighted with a matrix $\boldsymbol{W}:=\hat{\boldsymbol{\Phi}}{\hat{\boldsymbol{\Lambda}}}_{\boldsymbol{x}}{\hat{\boldsymbol{\Sigma}}}^{\boldsymbol{-1}}$. In longitudinal measurement invariance testing, we strive to establish that these weights stay fixed across time; i.e., that for $\hat{\xi}_{s}\boldsymbol{=}\boldsymbol{W}_{s}\boldsymbol{x}_{s}$ and $\hat{\xi}_{t}\boldsymbol{=}\boldsymbol{W}_{t}\boldsymbol{x}_{t}$ at any times *s* and *t*, the equality $\boldsymbol{W}_{s}\boldsymbol{=}\boldsymbol{W}_{t}$ actually holds. But, if that is the case, then also $f(\boldsymbol{W}_{s}\boldsymbol{)=}f\boldsymbol{(}\boldsymbol{W}_{t}\boldsymbol{)}$ for any deterministic function *f*. In particular, this holds for the function *f* that takes ***W****_s_* into a row vector of ones, **1***^T^*, that has equally many elements as there are items in the measurement inventory in question. Because

$\boldsymbol{1}^{T}\boldsymbol{x}=\sum_{i=1}^{K} x_{i}$,

for *K*-vectors, establishing measurement invariance of a factor model established that sum scores are just as measurement invariant as factor scores based on the regression method (provided *f* exists, as typically expected).

While there are other methods to derive factor scores and much more complex item-response models, in what comes to single patient’s estimated latent score, the methods tend to take a fixed function of her observed scores. If that fixed function stays invariant across time, then so do its (surjective) transformations. In this sense, measurement invariance of factors and sum scores are closely related concepts. And just like sum scores, factor scores carry with them the errors that the observations have, although they reduce the error via law of large numbers applied to summation of multiple items to single factor—just as do sum scores. That is at the level of any single patient. As a population model, however, sum scores may be much less accurate than factor models. At the population level, a ‘true’ factor model can indeed eliminate all measurement error with a sufficient sample size.

# A simulation example of Davidian curve estimation

It is critical for the reader of this manuscript to understand that our methodology was designed to be robust to variations in the specific part of an underlying latent-factor distribution the observed ordinal items happen to ‘measure’. Therefore, we explicitly demonstrate this using a simple simulation data together with Davian curve estimation (Chalmers, 2012; Woods, 2015). Specifically, we simulated 2000 observations from a normal distribution of mean 0 and variance 2^-1^ to represent samples from a latent trait. Seven item liabilities were derived from these by adding normally distributed unique item variances (mean 0 and variance 2^-1^), hence producing standard normal distributed item liabilities (Figure S5a for an example histogram). Two different sets of ordinal (Likert) items were created from these same simulated liability values, one that evenly samples the liability distributions and another that is more informative about the right tail of the liability distributions. Then, we tested whether our Davidian curve results correctly recovered the form of the latent-trait distribution (normal and symmetric) in both cases, without being fooled by the lopsided item informativeness in case of the right-tail sampling items. Before going to technical details, we note that Figure S5d revealed unbiased estimates in both the cases.

For the evenly sampling items, we used the threshold values of -1.5, -0.5, 0.5, 1.5 to divide the standard normal liabilities to five ordinal categories. The simulated sum score of seven such items distributed as shown in the histogram of Figure S5b. For the item set sampling the right tail of the latent distribution (and the liability distributions), we used the threshold values of 0, 0.5, 1, 1.5 to divide the standard normal liabilities to five ordinal categories. The simulated sum score of seven such items distributed as shown in the histogram of Figure S5c. However, Davidian curve estimation has been designed for estimating the shape of the *underlying* factor distribution. Thus, despite these seemingly great differences in the sum scores of evenly sampling vs. right-tail sampling items, both the sets of items were best fit with the same (the lowest tested) number of Davian parameters (3) and they produced near-identical estimates for the shape of the latent-trait distribution, correctly recovering its symmetric form (Figure S5d). For comprehensiveness, the full simulation code is given in the below section on “[a]n R script to test Davidian curve estimation using simulated data”.

# On the structural equation model constraints and scale setting

Configural invariance model can be illustrated with the schematic Figure S6 for two successive measurements. Two only for simplicity. The model generalizes in straightforward and intuitive way to arbitrary number of assessments. The model allows latent factors and observed items to correlate with their own values over time but not directly cross items: the cross-item correlations are modeled only via the loadings of items on the latent factor. Previous research by Liu et al. (2017) largely reports the lavaan constraints needed for longitudinal measurement invariance (LMI) testing, but Murray et al. (2020) complements by adjusting latent-trait mean and variance of the first assessment time point to 0 and 1, respectively, which aids interpretation of treatment course against a standard scale at therapy entry point. To sum up, the following constraints were defined in lavaan to identify the respective LMI models:

| **Longitudinal measurement invariance model** | **New constraints introduced to previous model** |
| --- | --- |
| 1. Configural invariance | See Figure S6 for the general model structure. In addition, we need for identification the following constraints:   - One factor loading fixed across time ($\lambda_{ViTt}$ = $\lambda_{ViT1}$ for all *t* and one *i*) - One threshold parameter per item fixed across time for all items, two thresholds for one of the items ($\tau_{k,ViTt}= \tau_{k,ViT1}$ for one *k* and all *i* and *t*, and for one *j* and one *s* ≠ *k* $\tau_{s,VjTt}= \tau_{s,VjT1}$) |
| 1. Loadings invariance | Set factor loadings equal across time, or measurement occasions ($\lambda_{ViT1}=\lambda_{ViTt}$ for all *i* and *t*). |
| 1. Threshold invariance | All threshold parameters per item fixed across time ($\tau_{k,ViTt}= \tau_{k,ViT1}$ for all *k*, *i*, and *t*) |
| 1. Unique factor invariance | All unique variances fixed to 1 ($\sigma_{ViTt}=1$ for all *i* and *t*) |

Further details on these measurement invariance models are available in Liu et al. (2017) and Murray et al. (2020). In the below section on printed model outputs, we deliver lavaan outputs from fitted unique factor invariance models with the widely-used Mplus style of reporting.

# On tail attrition and variance

In the main text, we observed session-by-session increasing latent-trait variance in the LMI model. We argued that the observation could have derived from the LMI model’s wrong distributional assumptions rather than genuinely increasing latent trait variance. Because we could not adjust for the possible non-ignorable patient attrition for the reasons outline above and in the main text, a reader might ponder what effects selective attrition from a distribution’s tails (e.g., high- or low-responders dropping out) has on its variance. Thus, we demonstrate here that such attrition generally reduces rather than increases variance, and therefore is not a straightforward explanation for the increasing variance.

Let us assume normally distributed trait *X*, standard normal without a loss of generality, and a threshold value *δ* such that we observe only those values of *X* for which -*δ* < *X* < *δ*. Then, it generally holds that the observed values have a lower variance than the original distribution, meaning that

Var(*X*|-*δ* < *X* < *δ*) < Var(*X*) = 1.

Indeed, the value of Var(*X*|-*δ* < *X* < *δ*) can be explicitly computed as

$1-\frac{2\delta\varphi(\delta)}{2\Phi\left( \delta\right)-1}$ ,

where $\varphi$ is the standard normal density function and $\Phi$ the standard normal cumulative distribution function (Johnson et al., 1994, page 158). This remains safely below 1 for possible values of *δ*, as shown by the Supplementary Figure S7. Thus, tail-attrition generally does not inflate variance.

It remains a possibility that non-ignorable attrition changes the shape of the latent-trait distribution instead of a genuine change in its shape during treatment. This possibility is exceedingly difficult to address via imputation methods because they typically make distributional assumptions (e.g., normal response or liability) when generating the imputations. For example, assuming underlying normality, imputing such data, and then finding more normally distributed data after imputation would prove nothing. However, irrespective of attrition, it seems likely that the shape of the patient symptom distribution is closer to that of the general population after treatment (after getting better) than before it. And, estimates of latent-trait distribution in the general population do indicate rightward skewed shapes, as we would expect under a genuine change of shape (e.g., Magnus & Liu, 2018).

## Supplementary references

Arndt, A., Lutz, W., Rubel, J., Berger, T., Meyer, B., Schröder, J., Späth, C., Hautzinger, M., Fuhr, K., Rose, M., Hohagen, F., Klein, J. P., & Moritz, S. (2020). Identifying change-dropout patterns during an Internet-based intervention for depression by applying the Muthen-Roy model. *Cognitive Behaviour Therapy*, *49*(1), 22–40. https://doi.org/10.1080/16506073.2018.1556331

Bollen, K. A. (1989). *Structural Equations with Latent Variables*. John Wiley & Sons, Inc.

Chalmers, R. P. (2012). mirt: A Multidimensional Item Response Theory package for the R environment. *Journal of Statistical Software*, *48*(6), 1–29.

Honaker, J., King, G., & Blackwell, M. (2011). Amelia II: A Program for Missing Data. *Journal of Statistical Software*, *45*(1), 1–47. https://doi.org/10.18637/jss.v045.i07

Johnson, N. L., Kotz, S., & Balakrishnan, N. (1994). *Continuous Univariate Distributions, Vol 1* (2nd ed.). John Wiley & Sons, Inc.

Liu, Y., Millsap, R. E., West, S. G., Tein, J.-Y., Tanaka, R., & Grimm, K. J. (2017). Testing measurement invariance in longitudinal data with ordered-categorical measures. *Psychological Methods*, *22*(3), 486–506. https://doi.org/10.1037/met0000075

Liu, Y., & Sriutaisuk, S. (2021). A Comparison of FIML- versus Multiple-imputation-based methods to test measurement invariance with incomplete ordinal variables. *Structural Equation Modeling: A Multidisciplinary Journal*, *28*(4), 590–608. https://doi.org/10.1080/10705511.2021.1876520

Magnus, B. E., & Liu, Y. (2018). A zero-inflated Box-Cox normal unipolar item response model for measuring constructs of psychopathology. *Applied Psychological Measurement*, *42*(7), 571–589. https://doi.org/10.1177/0146621618758291

Murray, A. L., McKenzie, K., Murray, K., & Richelieu, M. (2020). Examining response shifts in the Clinical Outcomes in Routine Evaluation- Outcome Measure (CORE-OM). *British Journal of Guidance & Counselling*, *48*(2), 276–288. https://doi.org/10.1080/03069885.2018.1483007

van Buuren, S., & Groothuis-Oudshoorn, K. (2011). mice: Multivariate imputation by chained equations in R. *Journal of Statistical Software*, *45*(3), 1–67.

Woods, C. M. (2015). Estimating the latent density in unidimensional IRT to permit non-normality. In S. P. Reise & D. A. Revicki (Eds.), *Handbook of Item Response Theory Modeling: Applications to typical performance assessment* (pp. 60–84). Taylor & Francis.

Zahery, M., Maes, H. H., & Neale, M. C. (2017). CSOLNP: Numerical Optimization Engine for Solving Non-linearly Constrained Problems. *Twin Research and Human Genetics*, *20*(4), 290–297. https://doi.org/10.1017/thg.2017.28

## Supplementary figures


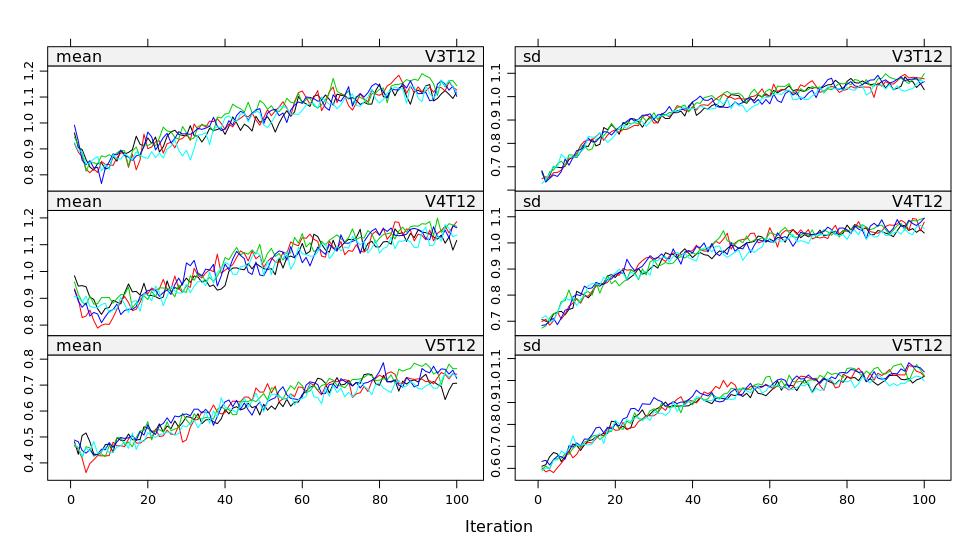


Figure S1. Examples of badly behaving mice chains. *Five chains shown per GAD-7 variable per panel, first column of panels for means, second column for standard deviations. For imputations to be trustworthy, chained equations should settle to some item means and standard deviations for the imputed values but here we observe increases even after 100 iterations (the default is 5 iterations). The chains are from multiple imputations using predictive mean matching regressions but ordered logit regressions led to similar problems.*


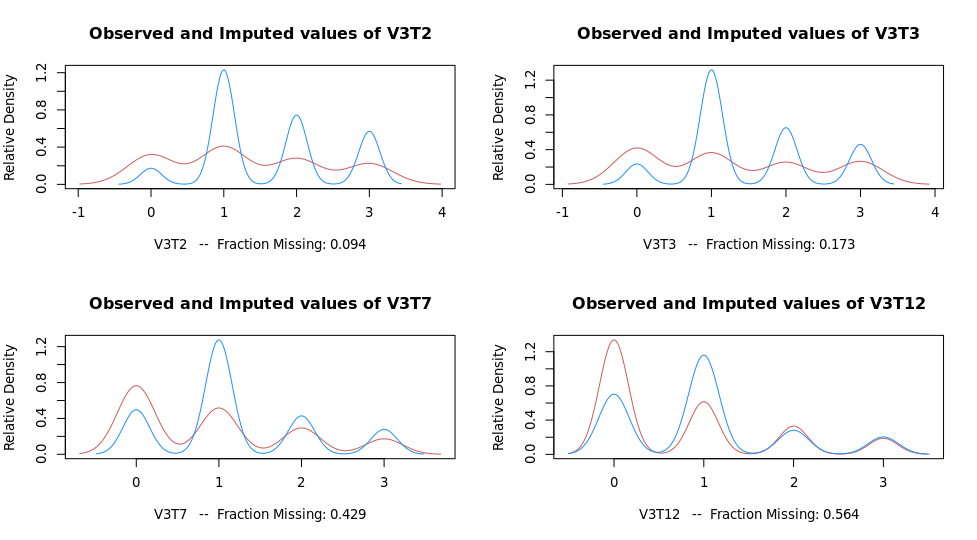


Figure S2. Observed (blue) and imputed (red) value densities from Amelia II imputations. Shown for item #3 (variable 3, or V3) of Generalized Anxiety Disorder Assessment-7 inventory at different sessions, or assessment times (T2-T12).


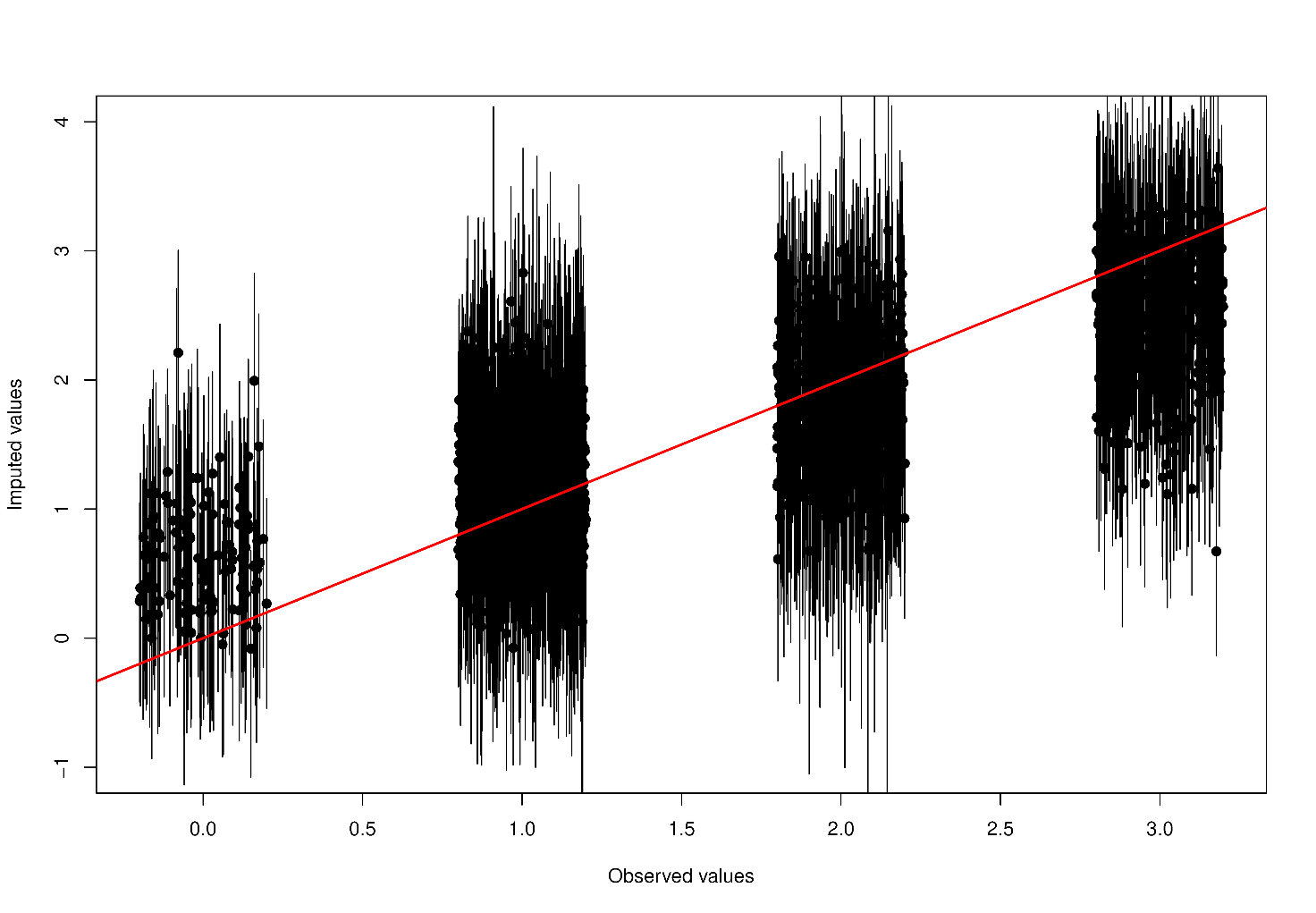


Figure S3. Overimputations for V3T2 of Fig. S2. Red line (*y* = *x-*axis) gives the estimation target when plotting (over)imputed values against the observations. Black balls show the estimates and their whiskers the 90% confidence interval of the estimate. Jitter (uniformly distributed random values between -0.2 and 0.2) was added to *x*-axis values of the plot because otherwise the integer-valued categories would not be distinguishable from each other.


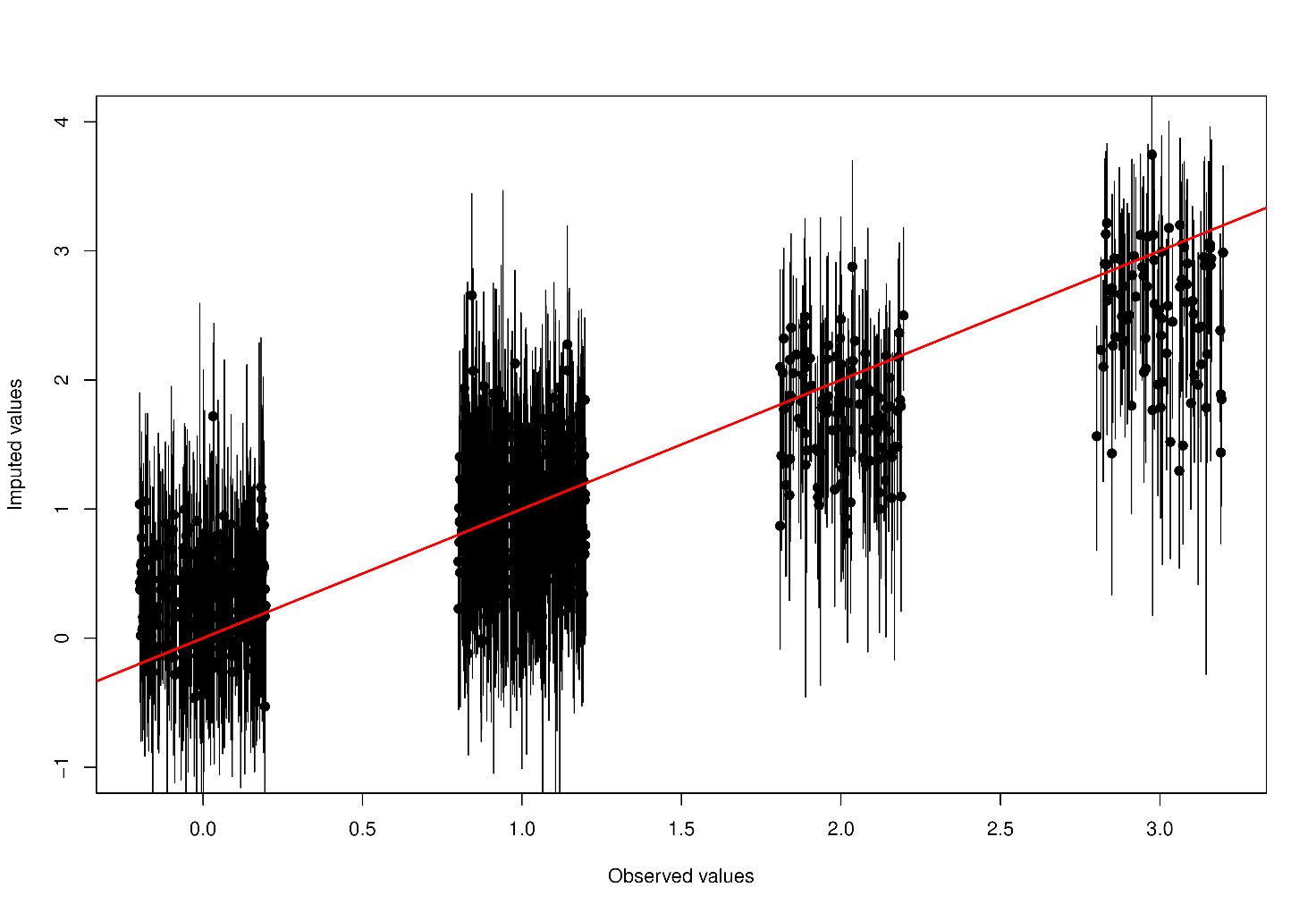


Figure S4. Overimputations for V3T12 of Fig. S2. Red line (*y* = *x-*axis) gives the estimation target when plotting (over)imputed values against the observations. Black balls show the estimates and their whiskers the 90% confidence interval of the estimate. Jitter (uniformly distributed random values between -0.2 and 0.2) was added to *x*-axis values of the plot because otherwise the integer-valued categories would not be distinguishable from each other.


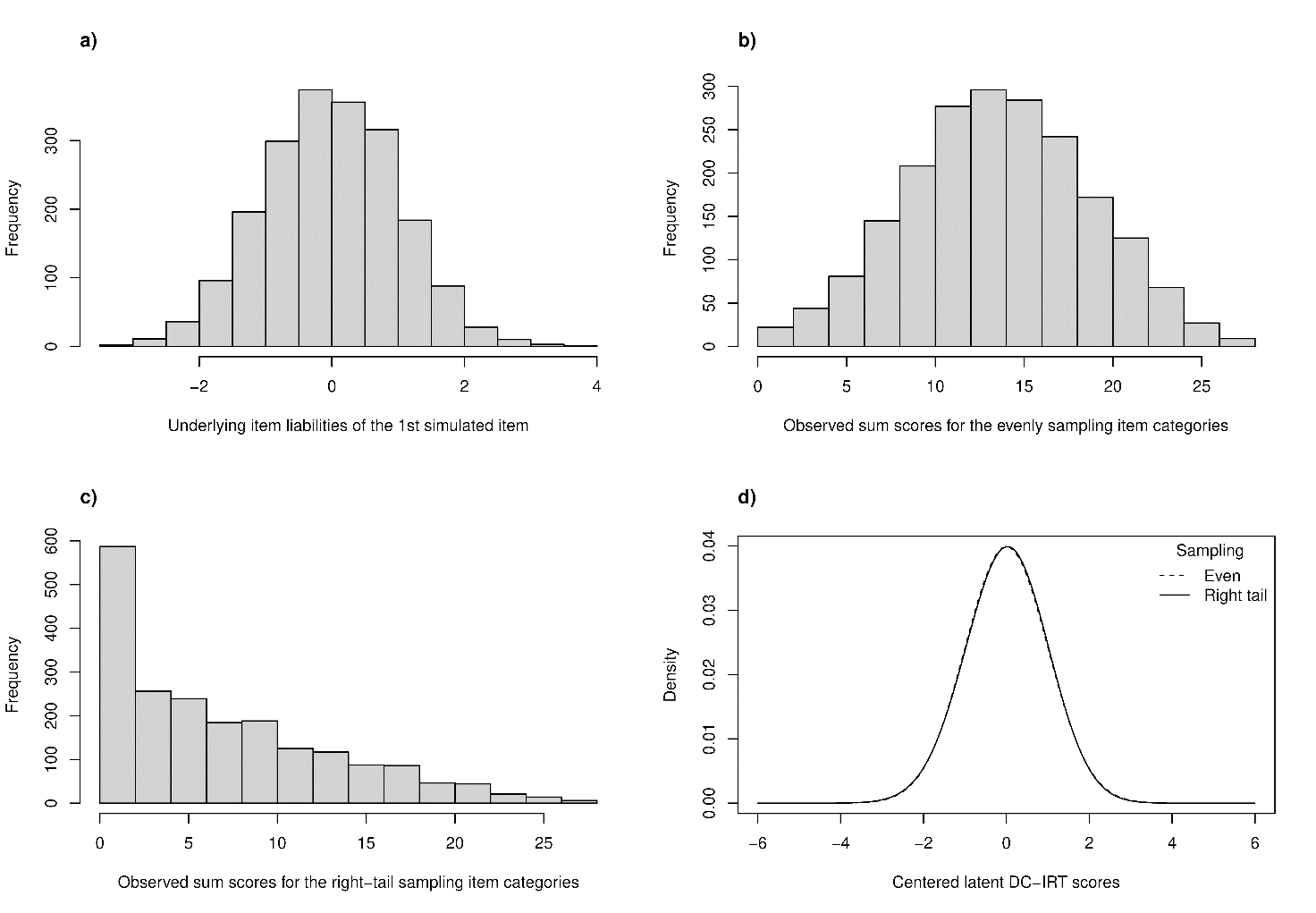


Figure S5. Simulation results for Davidian curve estimation. **a)** Histogram of underlying liabilities of item #1 out of seven. All items had similar characteristics and half of their total variance derived from the same latent trait. **b)** Histogram of observed sum scores for the set of ordinal items whose categories evenly sampled item liabilities from both sides of the liability distribution. **c)** Histogram of observed sum scores for the set of ordinal items whose categories accurately sampled only the right-tail of the liability distributions, with all liability values < 0 (half of all observations) falling to the lowest item categories. **d)** Davidian curve estimated latent-trait distributions for the item sets related to panel b (dashed line) and c (solid line). Note that the lines almost exactly overlap, suggesting the method recovered the same correct underlying distribution irrespective of the item type.


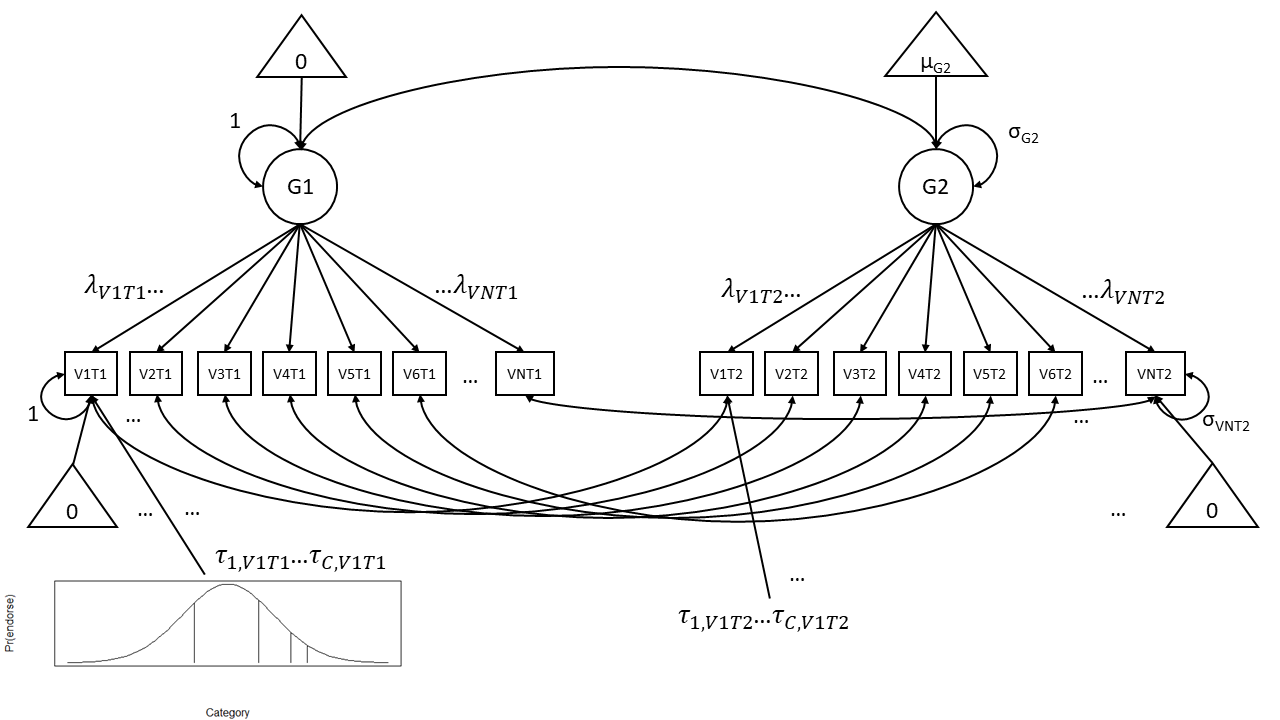


Figure S6. Sketched path diagram for configural invariance model. Triangles represent constant intercepts, circles latent variables, and boxes observed variables. Threshold values (τ) are estimated for each observed variable (V) at each measurement occasion/time (T) but figure shows only a couple examples for clarity. Similarly, each variable has an intercept of 0 and some unique variance, but only a couple examples are shown in the figure. Associated with each item’s set of thresholds is a standard normal distributed latent liability but that involves no additional parameters.


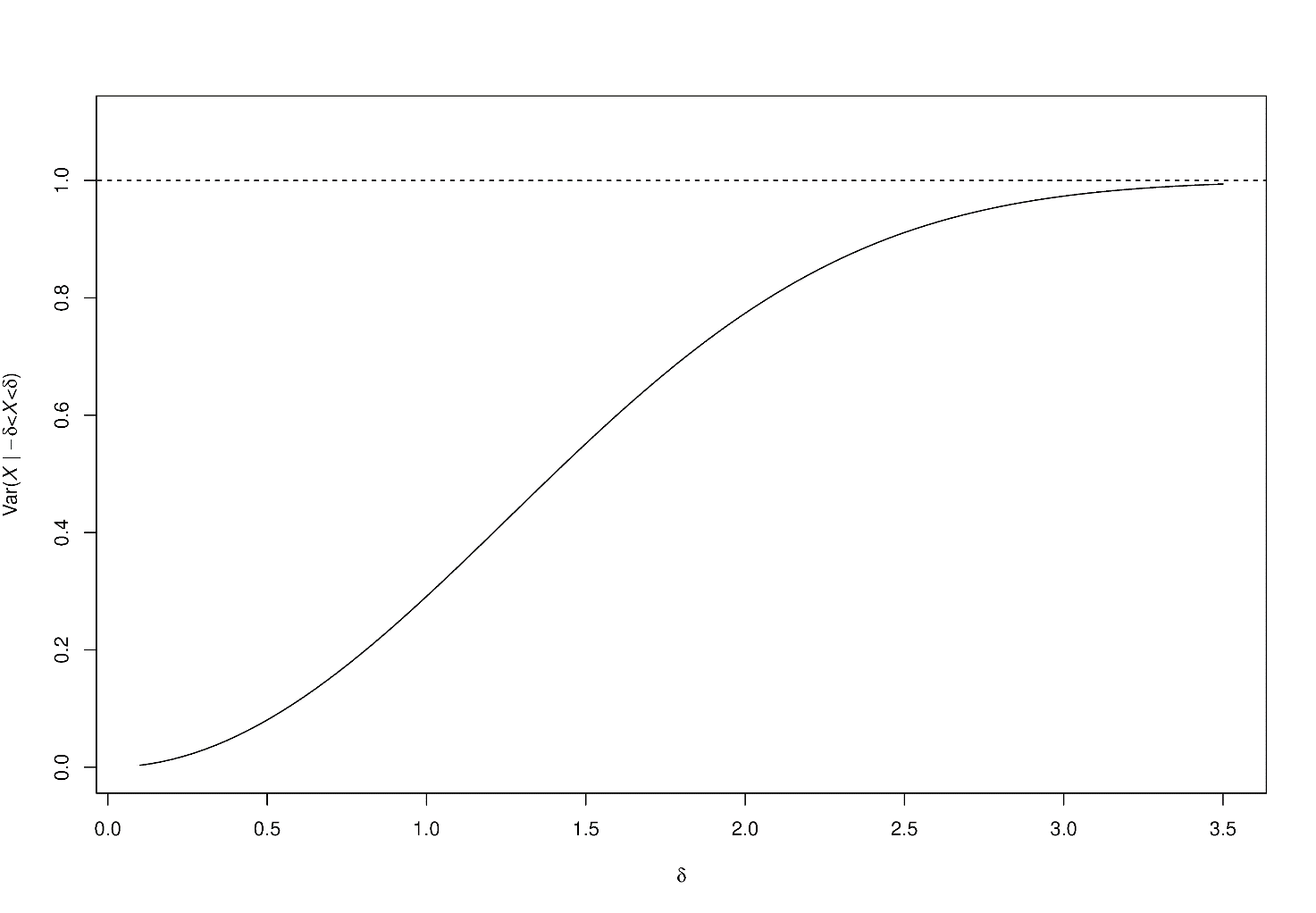


Figure S7. Truncated normal variance. Variance of (*X* |-*δ* < *X* < *δ*) as a function of *δ* when *X* has a standard normal distribution.

## An R script to test Davidian curve estimation using simulated data

# Simulate the underlying continuous-valued data

set.seed(2017)

n <- 2000

x <- rnorm(n)/sqrt(2) # Underlying trait

xx <- matrix(0,n,7) # For item liabilities

for (i in 1:7){xx[,i] <- x + rnorm(n)/sqrt(2)}

# Evenly sampling items

dw1 <- data.frame(xx)

names(dw1) <- paste0("V",1:7)

for (i in 1:7){dw1[,i] <- cut(dw1[,i], breaks = c(-Inf,-1.5,-0.5,0.5,1.5,Inf))}

dw1 <- sapply(dw1, function(x) as.numeric(ordered(x, levels = levels(dw1[,1]))))

# Items sampling high latent-trait values

dw12 <- data.frame(xx)

names(dw12) <- paste0("V",1:7)

for (i in 1:7){dw12[,i] <- cut(dw12[,i], breaks = c(-Inf,0,0.5,1,1.5,Inf))}

dw12 <- sapply(dw12, function(x) as.numeric(ordered(x, levels = levels(dw12[,1]))))

# Fit DC-IRT models to evenly sampling items

library(mirt)

dav1 <- vector("list", 5)

for (i in 1:5) dav1[[i]] <- mirt(dw1, 1, dentype = paste0('Davidian-',i+2))

dav1HQs <- rep(0,5)

for (i in 1:5) dav1HQs[i] <- extract.mirt(dav1[[i]], what = "HQ")

which.min(dav1HQs) + 2 # HQ-best number of Davidian parameters

# Fit DC-IRT models to items sampling positive end of the trait

dav12 <- vector("list", 5)

for (i in 1:5) dav12[[i]] <- mirt(dw12, 1, dentype = paste0('Davidian-',i+2))

dav12HQs <- rep(0,5)

for (i in 1:5) dav12HQs[i] <- extract.mirt(dav12[[i]], what = "HQ")

which.min(dav12HQs) + 2 # HQ-best number of Davidian parameters

# Examine HQ-best models

dav1_HQb <- dav1[[which.min(dav1HQs)]]

dav12_HQb <- dav12[[which.min(dav12HQs)]]

# Produce plots of data and best models

# postscript("fig_DCIRT_simulation.eps")

par(mfrow=c(2,2))

hist(xx[,1], main = "",

xlab = "Underlying item liabilities of the 1st simulated item")

title("a)", adj=0)

hist(rowSums(dw1)-7, main = "",

xlab = "Observed sum scores for the evenly sampling item categories")

title("b)", adj=0)

hist(rowSums(dw12)-7, main = "",

xlab = "Observed sum scores for the right-tail sampling item categories")

title("c)", adj=0)

plot(dav12_HQb@Model$Theta, dav12_HQb@Internals$Prior[[1]], type="l",

xlab = "Centered latent DC-IRT scores", ylab = "Density")

lines(dav1_HQb@Model$Theta, dav1_HQb@Internals$Prior[[1]], lty = 2)

legend("topright", legend = c("Even", "Right tail"), title = "Sampling",

lty = c(2,1), bty = "n")

title("d)", adj=0)

# dev.off()

## Printed model output from lavaan R package

For comprehensiveness, we report below direct lavaan-package prints for the uniform factor invariance models. Even here, we omit fully comprehensive reporting of every model in the LMI testing sequences because the point of that exercise is to condense information rather than display hundreds of pages of parameter estimates to a human reader.

### Online Psychotherapy for generalized anxiety disorder

#### Unique factor invariance model

lavaan 0.6-7 ended normally after 219 iterations

Estimator DWLS

Optimization method NLMINB

Number of free parameters 1012

Number of equality constraints 336

Number of observations 2218

Number of missing patterns 13

Model Test User Model:

Standard Robust

Test Statistic 10154.033 7291.080

Degrees of freedom 3692 3692

P-value (Chi-square) 0.000 0.000

Scaling correction factor 2.169

Shift parameter 2609.479

simple second-order correction (WLSMV)

Model Test Baseline Model:

Test statistic 1297760.852 147649.575

Degrees of freedom 4095 4095

P-value 0.000 0.000

Scaling correction factor 9.012

User Model versus Baseline Model:

Comparative Fit Index (CFI) 0.995 0.975

Tucker-Lewis Index (TLI) 0.994 0.972

Robust Comparative Fit Index (CFI) NA

Robust Tucker-Lewis Index (TLI) NA

Root Mean Square Error of Approximation:

RMSEA 0.028 0.021

90 Percent confidence interval - lower 0.027 0.020

90 Percent confidence interval - upper 0.029 0.022

P-value RMSEA <= 0.05 1.000 1.000

Robust RMSEA NA

90 Percent confidence interval - lower NA

90 Percent confidence interval - upper NA

Standardized Root Mean Square Residual:

SRMR 0.045 0.045

Weighted Root Mean Square Residual:

WRMR 1.525 1.525

Parameter Estimates:

Standard errors Robust.sem

Information Expected

Information saturated (h1) model Unstructured

Latent Variables:

Estimate Std.Err z-value P(>|z|)

G1 =~

V1T1 (lmb1) 1.161 0.034 34.618 0.000

V2T1 (lmb2) 1.480 0.044 33.312 0.000

V3T1 (lmb3) 1.422 0.041 34.786 0.000

V4T1 (lmb4) 1.047 0.034 30.970 0.000

V5T1 (lmb5) 0.881 0.038 23.399 0.000

V6T1 (lmb6) 0.770 0.029 26.539 0.000

V7T1 (lmb7) 0.846 0.032 26.114 0.000

G2 =~

V1T2 (lmb1) 1.161 0.034 34.618 0.000

V2T2 (lmb2) 1.480 0.044 33.312 0.000

V3T2 (lmb3) 1.422 0.041 34.786 0.000

V4T2 (lmb4) 1.047 0.034 30.970 0.000

V5T2 (lmb5) 0.881 0.038 23.399 0.000

V6T2 (lmb6) 0.770 0.029 26.539 0.000

V7T2 (lmb7) 0.846 0.032 26.114 0.000

G3 =~

V1T3 (lmb1) 1.161 0.034 34.618 0.000

V2T3 (lmb2) 1.480 0.044 33.312 0.000

V3T3 (lmb3) 1.422 0.041 34.786 0.000

V4T3 (lmb4) 1.047 0.034 30.970 0.000

V5T3 (lmb5) 0.881 0.038 23.399 0.000

V6T3 (lmb6) 0.770 0.029 26.539 0.000

V7T3 (lmb7) 0.846 0.032 26.114 0.000

G4 =~

V1T4 (lmb1) 1.161 0.034 34.618 0.000

V2T4 (lmb2) 1.480 0.044 33.312 0.000

V3T4 (lmb3) 1.422 0.041 34.786 0.000

V4T4 (lmb4) 1.047 0.034 30.970 0.000

V5T4 (lmb5) 0.881 0.038 23.399 0.000

V6T4 (lmb6) 0.770 0.029 26.539 0.000

V7T4 (lmb7) 0.846 0.032 26.114 0.000

G5 =~

V1T5 (lmb1) 1.161 0.034 34.618 0.000

V2T5 (lmb2) 1.480 0.044 33.312 0.000

V3T5 (lmb3) 1.422 0.041 34.786 0.000

V4T5 (lmb4) 1.047 0.034 30.970 0.000

V5T5 (lmb5) 0.881 0.038 23.399 0.000

V6T5 (lmb6) 0.770 0.029 26.539 0.000

V7T5 (lmb7) 0.846 0.032 26.114 0.000

G6 =~

V1T6 (lmb1) 1.161 0.034 34.618 0.000

V2T6 (lmb2) 1.480 0.044 33.312 0.000

V3T6 (lmb3) 1.422 0.041 34.786 0.000

V4T6 (lmb4) 1.047 0.034 30.970 0.000

V5T6 (lmb5) 0.881 0.038 23.399 0.000

V6T6 (lmb6) 0.770 0.029 26.539 0.000

V7T6 (lmb7) 0.846 0.032 26.114 0.000

G7 =~

V1T7 (lmb1) 1.161 0.034 34.618 0.000

V2T7 (lmb2) 1.480 0.044 33.312 0.000

V3T7 (lmb3) 1.422 0.041 34.786 0.000

V4T7 (lmb4) 1.047 0.034 30.970 0.000

V5T7 (lmb5) 0.881 0.038 23.399 0.000

V6T7 (lmb6) 0.770 0.029 26.539 0.000

V7T7 (lmb7) 0.846 0.032 26.114 0.000

G8 =~

V1T8 (lmb1) 1.161 0.034 34.618 0.000

V2T8 (lmb2) 1.480 0.044 33.312 0.000

V3T8 (lmb3) 1.422 0.041 34.786 0.000

V4T8 (lmb4) 1.047 0.034 30.970 0.000

V5T8 (lmb5) 0.881 0.038 23.399 0.000

V6T8 (lmb6) 0.770 0.029 26.539 0.000

V7T8 (lmb7) 0.846 0.032 26.114 0.000

G9 =~

V1T9 (lmb1) 1.161 0.034 34.618 0.000

V2T9 (lmb2) 1.480 0.044 33.312 0.000

V3T9 (lmb3) 1.422 0.041 34.786 0.000

V4T9 (lmb4) 1.047 0.034 30.970 0.000

V5T9 (lmb5) 0.881 0.038 23.399 0.000

V6T9 (lmb6) 0.770 0.029 26.539 0.000

V7T9 (lmb7) 0.846 0.032 26.114 0.000

G10 =~

V1T10 (lmb1) 1.161 0.034 34.618 0.000

V2T10 (lmb2) 1.480 0.044 33.312 0.000

V3T10 (lmb3) 1.422 0.041 34.786 0.000

V4T10 (lmb4) 1.047 0.034 30.970 0.000

V5T10 (lmb5) 0.881 0.038 23.399 0.000

V6T10 (lmb6) 0.770 0.029 26.539 0.000

V7T10 (lmb7) 0.846 0.032 26.114 0.000

G11 =~

V1T11 (lmb1) 1.161 0.034 34.618 0.000

V2T11 (lmb2) 1.480 0.044 33.312 0.000

V3T11 (lmb3) 1.422 0.041 34.786 0.000

V4T11 (lmb4) 1.047 0.034 30.970 0.000

V5T11 (lmb5) 0.881 0.038 23.399 0.000

V6T11 (lmb6) 0.770 0.029 26.539 0.000

V7T11 (lmb7) 0.846 0.032 26.114 0.000

G12 =~

V1T12 (lmb1) 1.161 0.034 34.618 0.000

V2T12 (lmb2) 1.480 0.044 33.312 0.000

V3T12 (lmb3) 1.422 0.041 34.786 0.000

V4T12 (lmb4) 1.047 0.034 30.970 0.000

V5T12 (lmb5) 0.881 0.038 23.399 0.000

V6T12 (lmb6) 0.770 0.029 26.539 0.000

V7T12 (lmb7) 0.846 0.032 26.114 0.000

G13 =~

V1T13 (lmb1) 1.161 0.034 34.618 0.000

V2T13 (lmb2) 1.480 0.044 33.312 0.000

V3T13 (lmb3) 1.422 0.041 34.786 0.000

V4T13 (lmb4) 1.047 0.034 30.970 0.000

V5T13 (lmb5) 0.881 0.038 23.399 0.000

V6T13 (lmb6) 0.770 0.029 26.539 0.000

V7T13 (lmb7) 0.846 0.032 26.114 0.000

Covariances:

Estimate Std.Err z-value P(>|z|)

G1 ~~

G2 0.789 0.022 36.141 0.000

G3 0.722 0.027 26.855 0.000

G4 0.695 0.029 23.662 0.000

G5 0.674 0.033 20.188 0.000

G6 0.642 0.036 17.897 0.000

G7 0.637 0.038 16.860 0.000

G8 0.597 0.040 14.832 0.000

G9 0.595 0.043 13.871 0.000

G10 0.539 0.045 12.045 0.000

G11 0.576 0.047 12.306 0.000

G12 0.565 0.050 11.249 0.000

G13 0.554 0.132 4.208 0.000

G2 ~~

G3 0.911 0.040 22.870 0.000

G4 0.887 0.042 21.280 0.000

G5 0.870 0.045 19.508 0.000

G6 0.851 0.047 18.245 0.000

G7 0.803 0.048 16.669 0.000

G8 0.755 0.050 15.233 0.000

G9 0.797 0.051 15.516 0.000

G10 0.723 0.053 13.590 0.000

G11 0.803 0.055 14.554 0.000

G12 0.784 0.060 13.182 0.000

G13 0.770 0.145 5.326 0.000

G3 ~~

G4 1.004 0.047 21.286 0.000

G5 0.946 0.048 19.523 0.000

G6 0.946 0.052 18.348 0.000

G7 0.943 0.054 17.619 0.000

G8 0.943 0.057 16.468 0.000

G9 0.892 0.056 16.034 0.000

G10 0.884 0.058 15.140 0.000

G11 0.966 0.063 15.399 0.000

G12 0.938 0.065 14.472 0.000

G13 1.013 0.148 6.826 0.000

G4 ~~

G5 1.109 0.056 19.671 0.000

G6 1.052 0.058 18.170 0.000

G7 0.982 0.058 16.816 0.000

G8 0.940 0.060 15.792 0.000

G9 0.946 0.060 15.888 0.000

G10 0.920 0.063 14.671 0.000

G11 1.011 0.066 15.349 0.000

G12 0.993 0.067 14.891 0.000

G13 0.990 0.151 6.570 0.000

G5 ~~

G6 1.222 0.065 18.826 0.000

G7 1.089 0.064 17.122 0.000

G8 1.073 0.064 16.645 0.000

G9 1.013 0.064 15.846 0.000

G10 1.070 0.069 15.455 0.000

G11 1.164 0.073 15.885 0.000

G12 1.134 0.074 15.222 0.000

G13 1.270 0.156 8.144 0.000

G6 ~~

G7 1.214 0.069 17.610 0.000

G8 1.133 0.069 16.521 0.000

G9 1.110 0.069 16.176 0.000

G10 1.071 0.071 15.099 0.000

G11 1.140 0.074 15.333 0.000

G12 1.147 0.076 15.037 0.000

G13 1.365 0.161 8.475 0.000

G7 ~~

G8 1.270 0.075 16.894 0.000

G9 1.178 0.073 16.214 0.000

G10 1.116 0.073 15.345 0.000

G11 1.226 0.079 15.619 0.000

G12 1.246 0.080 15.604 0.000

G13 1.364 0.166 8.232 0.000

G8 ~~

G9 1.291 0.079 16.403 0.000

G10 1.223 0.079 15.493 0.000

G11 1.287 0.084 15.284 0.000

G12 1.307 0.085 15.322 0.000

G13 1.353 0.170 7.975 0.000

G9 ~~

G10 1.283 0.082 15.592 0.000

G11 1.314 0.084 15.555 0.000

G12 1.332 0.086 15.536 0.000

G13 1.529 0.168 9.095 0.000

G10 ~~

G11 1.458 0.094 15.543 0.000

G12 1.439 0.094 15.322 0.000

G13 1.527 0.178 8.592 0.000

G11 ~~

G12 1.561 0.101 15.473 0.000

G13 1.739 0.181 9.596 0.000

G12 ~~

G13 1.730 0.182 9.514 0.000

.V1T1 ~~

.V1T2 0.487 0.036 13.505 0.000

.V1T3 0.402 0.043 9.291 0.000

.V1T4 0.310 0.051 6.052 0.000

.V1T5 0.263 0.056 4.657 0.000

.V1T6 0.181 0.061 2.950 0.003

.V1T7 0.175 0.062 2.839 0.005

.V1T8 0.194 0.071 2.708 0.007

.V1T9 0.166 0.074 2.239 0.025

.V1T10 0.209 0.077 2.706 0.007

.V1T11 0.303 0.078 3.854 0.000

.V1T12 0.154 0.080 1.934 0.053

.V1T13 0.699 0.152 4.601 0.000

.V1T2 ~~

.V1T3 0.364 0.040 9.020 0.000

.V1T4 0.327 0.046 7.189 0.000

.V1T5 0.240 0.053 4.537 0.000

.V1T6 0.242 0.056 4.312 0.000

.V1T7 0.187 0.059 3.148 0.002

.V1T8 0.288 0.061 4.734 0.000

.V1T9 0.205 0.065 3.157 0.002

.V1T10 0.190 0.072 2.637 0.008

.V1T11 0.236 0.070 3.350 0.001

.V1T12 0.215 0.080 2.697 0.007

.V1T13 0.628 0.181 3.474 0.001

.V1T3 ~~

.V1T4 0.393 0.044 8.978 0.000

.V1T5 0.245 0.049 5.012 0.000

.V1T6 0.203 0.054 3.726 0.000

.V1T7 0.110 0.057 1.944 0.052

.V1T8 0.130 0.060 2.171 0.030

.V1T9 0.081 0.067 1.201 0.230

.V1T10 0.196 0.074 2.669 0.008

.V1T11 0.068 0.070 0.964 0.335

.V1T12 0.103 0.078 1.327 0.184

.V1T13 0.212 0.158 1.343 0.179

.V1T4 ~~

.V1T5 0.364 0.044 8.255 0.000

.V1T6 0.233 0.049 4.755 0.000

.V1T7 0.120 0.058 2.083 0.037

.V1T8 0.202 0.064 3.175 0.001

.V1T9 0.147 0.063 2.348 0.019

.V1T10 0.054 0.073 0.748 0.455

.V1T11 0.222 0.064 3.472 0.001

.V1T12 0.217 0.077 2.808 0.005

.V1T13 0.341 0.156 2.189 0.029

.V1T5 ~~

.V1T6 0.379 0.044 8.703 0.000

.V1T7 0.210 0.054 3.917 0.000

.V1T8 0.212 0.060 3.510 0.000

.V1T9 0.191 0.059 3.222 0.001

.V1T10 0.272 0.068 3.986 0.000

.V1T11 0.239 0.068 3.533 0.000

.V1T12 0.225 0.069 3.276 0.001

.V1T13 0.316 0.152 2.083 0.037

.V1T6 ~~

.V1T7 0.467 0.043 10.750 0.000

.V1T8 0.341 0.057 6.021 0.000

.V1T9 0.194 0.057 3.438 0.001

.V1T10 0.229 0.063 3.608 0.000

.V1T11 0.260 0.062 4.155 0.000

.V1T12 0.367 0.064 5.775 0.000

.V1T13 0.376 0.121 3.104 0.002

.V1T7 ~~

.V1T8 0.396 0.053 7.491 0.000

.V1T9 0.357 0.054 6.670 0.000

.V1T10 0.155 0.067 2.306 0.021

.V1T11 0.257 0.068 3.757 0.000

.V1T12 0.338 0.068 4.956 0.000

.V1T13 0.150 0.167 0.896 0.370

.V1T8 ~~

.V1T9 0.407 0.053 7.626 0.000

.V1T10 0.243 0.062 3.941 0.000

.V1T11 0.430 0.057 7.523 0.000

.V1T12 0.324 0.060 5.423 0.000

.V1T13 0.196 0.172 1.143 0.253

.V1T9 ~~

.V1T10 0.266 0.056 4.771 0.000

.V1T11 0.211 0.057 3.682 0.000

.V1T12 0.337 0.060 5.585 0.000

.V1T13 0.129 0.196 0.659 0.510

.V1T10 ~~

.V1T11 0.325 0.054 6.044 0.000

.V1T12 0.237 0.059 4.030 0.000

.V1T13 -0.140 0.163 -0.862 0.389

.V1T11 ~~

.V1T12 0.426 0.046 9.294 0.000

.V1T13 0.304 0.146 2.084 0.037

.V1T12 ~~

.V1T13 0.185 0.181 1.020 0.308

.V2T1 ~~

.V2T2 0.274 0.047 5.867 0.000

.V2T3 0.305 0.052 5.850 0.000

.V2T4 0.222 0.061 3.642 0.000

.V2T5 0.222 0.067 3.305 0.001

.V2T6 0.093 0.073 1.282 0.200

.V2T7 0.037 0.082 0.451 0.652

.V2T8 -0.037 0.086 -0.426 0.670

.V2T9 0.103 0.090 1.139 0.255

.V2T10 0.120 0.100 1.208 0.227

.V2T11 0.127 0.101 1.261 0.207

.V2T12 0.113 0.096 1.182 0.237

.V2T13 -0.238 0.241 -0.988 0.323

.V2T2 ~~

.V2T3 0.326 0.048 6.820 0.000

.V2T4 0.249 0.058 4.293 0.000

.V2T5 0.278 0.061 4.542 0.000

.V2T6 0.280 0.071 3.938 0.000

.V2T7 0.131 0.080 1.633 0.102

.V2T8 0.027 0.078 0.341 0.733

.V2T9 0.116 0.085 1.365 0.172

.V2T10 0.187 0.086 2.185 0.029

.V2T11 0.127 0.094 1.358 0.175

.V2T12 0.053 0.094 0.562 0.574

.V2T13 -0.181 0.257 -0.704 0.481

.V2T3 ~~

.V2T4 0.329 0.053 6.259 0.000

.V2T5 0.264 0.060 4.418 0.000

.V2T6 0.279 0.065 4.329 0.000

.V2T7 0.114 0.075 1.523 0.128

.V2T8 0.066 0.072 0.918 0.358

.V2T9 0.234 0.076 3.069 0.002

.V2T10 0.245 0.093 2.625 0.009

.V2T11 0.266 0.095 2.803 0.005

.V2T12 0.145 0.094 1.540 0.124

.V2T13 0.095 0.267 0.355 0.723

.V2T4 ~~

.V2T5 0.343 0.051 6.682 0.000

.V2T6 0.180 0.061 2.970 0.003

.V2T7 0.192 0.069 2.766 0.006

.V2T8 0.122 0.079 1.549 0.121

.V2T9 0.279 0.086 3.248 0.001

.V2T10 0.152 0.090 1.694 0.090

.V2T11 0.125 0.095 1.320 0.187

.V2T12 0.170 0.099 1.713 0.087

.V2T13 -0.186 0.293 -0.634 0.526

.V2T5 ~~

.V2T6 0.287 0.058 4.929 0.000

.V2T7 0.129 0.073 1.766 0.077

.V2T8 0.047 0.081 0.583 0.560

.V2T9 0.285 0.081 3.527 0.000

.V2T10 0.140 0.084 1.671 0.095

.V2T11 0.219 0.088 2.484 0.013

.V2T12 0.225 0.092 2.446 0.014

.V2T13 -0.079 0.245 -0.322 0.748

.V2T6 ~~

.V2T7 0.267 0.060 4.443 0.000

.V2T8 0.281 0.074 3.780 0.000

.V2T9 0.221 0.077 2.878 0.004

.V2T10 0.248 0.087 2.839 0.005

.V2T11 0.119 0.091 1.303 0.192

.V2T12 0.242 0.086 2.823 0.005

.V2T13 0.282 0.208 1.360 0.174

.V2T7 ~~

.V2T8 0.110 0.070 1.574 0.116

.V2T9 0.143 0.075 1.924 0.054

.V2T10 0.188 0.083 2.250 0.024

.V2T11 0.046 0.092 0.496 0.620

.V2T12 -0.056 0.093 -0.603 0.547

.V2T13 0.145 0.220 0.660 0.509

.V2T8 ~~

.V2T9 0.496 0.064 7.781 0.000

.V2T10 0.221 0.079 2.796 0.005

.V2T11 0.292 0.085 3.447 0.001

.V2T12 0.180 0.083 2.169 0.030

.V2T13 -0.026 0.218 -0.117 0.906

.V2T9 ~~

.V2T10 0.425 0.069 6.180 0.000

.V2T11 0.295 0.079 3.720 0.000

.V2T12 0.249 0.078 3.186 0.001

.V2T13 -0.114 0.240 -0.475 0.635

.V2T10 ~~

.V2T11 0.298 0.075 3.958 0.000

.V2T12 0.290 0.074 3.945 0.000

.V2T13 -0.102 0.197 -0.520 0.603

.V2T11 ~~

.V2T12 0.376 0.061 6.204 0.000

.V2T13 -0.047 0.200 -0.235 0.814

.V2T12 ~~

.V2T13 -0.145 0.246 -0.592 0.554

.V3T1 ~~

.V3T2 0.225 0.047 4.742 0.000

.V3T3 0.304 0.052 5.803 0.000

.V3T4 0.245 0.060 4.075 0.000

.V3T5 0.225 0.063 3.545 0.000

.V3T6 0.082 0.070 1.171 0.242

.V3T7 0.128 0.077 1.653 0.098

.V3T8 0.077 0.085 0.905 0.365

.V3T9 0.210 0.088 2.396 0.017

.V3T10 0.086 0.093 0.923 0.356

.V3T11 0.298 0.091 3.264 0.001

.V3T12 0.139 0.088 1.582 0.114

.V3T13 -0.235 0.231 -1.017 0.309

.V3T2 ~~

.V3T3 0.313 0.045 6.999 0.000

.V3T4 0.352 0.054 6.567 0.000

.V3T5 0.315 0.056 5.602 0.000

.V3T6 0.249 0.066 3.753 0.000

.V3T7 0.252 0.072 3.498 0.000

.V3T8 0.210 0.078 2.700 0.007

.V3T9 0.244 0.082 2.956 0.003

.V3T10 0.214 0.089 2.407 0.016

.V3T11 0.237 0.084 2.817 0.005

.V3T12 0.256 0.085 3.016 0.003

.V3T13 -0.030 0.226 -0.132 0.895

.V3T3 ~~

.V3T4 0.295 0.051 5.807 0.000

.V3T5 0.201 0.054 3.704 0.000

.V3T6 0.202 0.064 3.163 0.002

.V3T7 0.218 0.069 3.152 0.002

.V3T8 0.138 0.075 1.840 0.066

.V3T9 0.297 0.083 3.582 0.000

.V3T10 0.274 0.081 3.370 0.001

.V3T11 0.158 0.078 2.028 0.043

.V3T12 0.113 0.082 1.382 0.167

.V3T13 0.108 0.231 0.470 0.639

.V3T4 ~~

.V3T5 0.368 0.052 7.139 0.000

.V3T6 0.311 0.060 5.204 0.000

.V3T7 0.351 0.068 5.130 0.000

.V3T8 0.060 0.077 0.786 0.432

.V3T9 0.341 0.078 4.390 0.000

.V3T10 0.314 0.089 3.549 0.000

.V3T11 0.271 0.082 3.301 0.001

.V3T12 0.273 0.085 3.213 0.001

.V3T13 0.155 0.217 0.714 0.476

.V3T5 ~~

.V3T6 0.206 0.058 3.544 0.000

.V3T7 0.316 0.067 4.718 0.000

.V3T8 0.147 0.077 1.911 0.056

.V3T9 0.219 0.072 3.033 0.002

.V3T10 0.145 0.079 1.825 0.068

.V3T11 0.165 0.076 2.163 0.031

.V3T12 0.230 0.081 2.832 0.005

.V3T13 0.323 0.202 1.599 0.110

.V3T6 ~~

.V3T7 0.195 0.061 3.226 0.001

.V3T8 0.186 0.071 2.624 0.009

.V3T9 0.198 0.077 2.580 0.010

.V3T10 0.122 0.076 1.607 0.108

.V3T11 0.275 0.079 3.480 0.001

.V3T12 0.156 0.080 1.954 0.051

.V3T13 -0.085 0.225 -0.378 0.705

.V3T7 ~~

.V3T8 0.337 0.067 5.030 0.000

.V3T9 0.329 0.068 4.814 0.000

.V3T10 0.337 0.084 3.994 0.000

.V3T11 0.285 0.078 3.654 0.000

.V3T12 0.248 0.083 2.978 0.003

.V3T13 0.093 0.197 0.473 0.636

.V3T8 ~~

.V3T9 0.388 0.064 6.056 0.000

.V3T10 0.322 0.076 4.238 0.000

.V3T11 0.235 0.074 3.183 0.001

.V3T12 0.366 0.084 4.382 0.000

.V3T13 0.284 0.230 1.232 0.218

.V3T9 ~~

.V3T10 0.308 0.068 4.507 0.000

.V3T11 0.362 0.060 6.015 0.000

.V3T12 0.240 0.069 3.475 0.001

.V3T13 0.131 0.196 0.667 0.505

.V3T10 ~~

.V3T11 0.239 0.064 3.718 0.000

.V3T12 0.267 0.068 3.929 0.000

.V3T13 0.182 0.176 1.033 0.302

.V3T11 ~~

.V3T12 0.348 0.057 6.145 0.000

.V3T13 0.069 0.199 0.349 0.727

.V3T12 ~~

.V3T13 -0.011 0.247 -0.044 0.965

.V4T1 ~~

.V4T2 0.522 0.031 16.687 0.000

.V4T3 0.427 0.037 11.422 0.000

.V4T4 0.411 0.042 9.796 0.000

.V4T5 0.370 0.045 8.206 0.000

.V4T6 0.391 0.050 7.823 0.000

.V4T7 0.513 0.050 10.316 0.000

.V4T8 0.408 0.059 6.862 0.000

.V4T9 0.385 0.059 6.556 0.000

.V4T10 0.381 0.062 6.173 0.000

.V4T11 0.368 0.065 5.631 0.000

.V4T12 0.331 0.065 5.058 0.000

.V4T13 0.355 0.151 2.344 0.019

.V4T2 ~~

.V4T3 0.506 0.033 15.467 0.000

.V4T4 0.446 0.039 11.313 0.000

.V4T5 0.353 0.045 7.780 0.000

.V4T6 0.378 0.050 7.608 0.000

.V4T7 0.381 0.053 7.119 0.000

.V4T8 0.323 0.062 5.216 0.000

.V4T9 0.366 0.063 5.794 0.000

.V4T10 0.358 0.062 5.797 0.000

.V4T11 0.338 0.067 5.024 0.000

.V4T12 0.323 0.068 4.756 0.000

.V4T13 0.328 0.153 2.149 0.032

.V4T3 ~~

.V4T4 0.476 0.037 13.015 0.000

.V4T5 0.431 0.040 10.722 0.000

.V4T6 0.416 0.045 9.234 0.000

.V4T7 0.397 0.048 8.193 0.000

.V4T8 0.416 0.052 7.999 0.000

.V4T9 0.464 0.051 9.098 0.000

.V4T10 0.348 0.054 6.417 0.000

.V4T11 0.359 0.053 6.720 0.000

.V4T12 0.351 0.063 5.551 0.000

.V4T13 0.377 0.136 2.771 0.006

.V4T4 ~~

.V4T5 0.501 0.036 13.814 0.000

.V4T6 0.500 0.041 12.182 0.000

.V4T7 0.397 0.049 8.108 0.000

.V4T8 0.418 0.053 7.936 0.000

.V4T9 0.404 0.054 7.534 0.000

.V4T10 0.382 0.055 6.991 0.000

.V4T11 0.445 0.058 7.710 0.000

.V4T12 0.384 0.061 6.350 0.000

.V4T13 0.443 0.149 2.975 0.003

.V4T5 ~~

.V4T6 0.535 0.037 14.273 0.000

.V4T7 0.489 0.049 10.075 0.000

.V4T8 0.427 0.049 8.741 0.000

.V4T9 0.485 0.054 9.056 0.000

.V4T10 0.411 0.055 7.525 0.000

.V4T11 0.330 0.056 5.906 0.000

.V4T12 0.278 0.065 4.274 0.000

.V4T13 0.391 0.136 2.884 0.004

.V4T6 ~~

.V4T7 0.495 0.044 11.367 0.000

.V4T8 0.528 0.050 10.479 0.000

.V4T9 0.586 0.047 12.489 0.000

.V4T10 0.526 0.051 10.278 0.000

.V4T11 0.512 0.054 9.560 0.000

.V4T12 0.492 0.063 7.816 0.000

.V4T13 0.639 0.134 4.771 0.000

.V4T7 ~~

.V4T8 0.584 0.042 14.008 0.000

.V4T9 0.478 0.050 9.640 0.000

.V4T10 0.503 0.054 9.252 0.000

.V4T11 0.407 0.058 6.988 0.000

.V4T12 0.409 0.059 6.931 0.000

.V4T13 0.464 0.128 3.638 0.000

.V4T8 ~~

.V4T9 0.517 0.045 11.603 0.000

.V4T10 0.520 0.049 10.546 0.000

.V4T11 0.539 0.051 10.484 0.000

.V4T12 0.465 0.058 8.054 0.000

.V4T13 0.452 0.139 3.253 0.001

.V4T9 ~~

.V4T10 0.643 0.043 15.014 0.000

.V4T11 0.588 0.050 11.861 0.000

.V4T12 0.529 0.056 9.519 0.000

.V4T13 0.658 0.120 5.483 0.000

.V4T10 ~~

.V4T11 0.591 0.043 13.607 0.000

.V4T12 0.643 0.046 14.024 0.000

.V4T13 0.523 0.136 3.846 0.000

.V4T11 ~~

.V4T12 0.636 0.040 15.923 0.000

.V4T13 0.593 0.122 4.872 0.000

.V4T12 ~~

.V4T13 0.522 0.143 3.650 0.000

.V5T1 ~~

.V5T2 0.681 0.024 28.323 0.000

.V5T3 0.561 0.032 17.489 0.000

.V5T4 0.559 0.036 15.492 0.000

.V5T5 0.463 0.040 11.704 0.000

.V5T6 0.551 0.042 13.111 0.000

.V5T7 0.539 0.047 11.508 0.000

.V5T8 0.620 0.050 12.345 0.000

.V5T9 0.531 0.052 10.229 0.000

.V5T10 0.563 0.057 9.915 0.000

.V5T11 0.544 0.058 9.416 0.000

.V5T12 0.549 0.061 8.974 0.000

.V5T13 0.488 0.137 3.554 0.000

.V5T2 ~~

.V5T3 0.665 0.025 26.577 0.000

.V5T4 0.616 0.032 19.402 0.000

.V5T5 0.547 0.037 14.671 0.000

.V5T6 0.580 0.039 14.927 0.000

.V5T7 0.500 0.047 10.518 0.000

.V5T8 0.536 0.051 10.412 0.000

.V5T9 0.517 0.053 9.774 0.000

.V5T10 0.543 0.057 9.510 0.000

.V5T11 0.499 0.058 8.606 0.000

.V5T12 0.478 0.059 8.132 0.000

.V5T13 0.539 0.135 3.998 0.000

.V5T3 ~~

.V5T4 0.659 0.029 23.049 0.000

.V5T5 0.621 0.035 17.529 0.000

.V5T6 0.572 0.041 14.127 0.000

.V5T7 0.540 0.044 12.160 0.000

.V5T8 0.590 0.048 12.340 0.000

.V5T9 0.484 0.051 9.550 0.000

.V5T10 0.479 0.052 9.217 0.000

.V5T11 0.446 0.054 8.236 0.000

.V5T12 0.439 0.058 7.558 0.000

.V5T13 0.356 0.129 2.757 0.006

.V5T4 ~~

.V5T5 0.644 0.032 20.115 0.000

.V5T6 0.680 0.036 18.674 0.000

.V5T7 0.672 0.044 15.243 0.000

.V5T8 0.684 0.047 14.575 0.000

.V5T9 0.530 0.047 11.188 0.000

.V5T10 0.547 0.052 10.558 0.000

.V5T11 0.621 0.052 11.978 0.000

.V5T12 0.526 0.058 9.125 0.000

.V5T13 0.365 0.161 2.258 0.024

.V5T5 ~~

.V5T6 0.732 0.032 23.048 0.000

.V5T7 0.676 0.042 16.040 0.000

.V5T8 0.757 0.044 17.252 0.000

.V5T9 0.640 0.047 13.654 0.000

.V5T10 0.684 0.048 14.217 0.000

.V5T11 0.586 0.051 11.468 0.000

.V5T12 0.601 0.054 11.132 0.000

.V5T13 0.523 0.141 3.714 0.000

.V5T6 ~~

.V5T7 0.711 0.036 19.829 0.000

.V5T8 0.744 0.042 17.848 0.000

.V5T9 0.659 0.045 14.670 0.000

.V5T10 0.698 0.048 14.607 0.000

.V5T11 0.570 0.051 11.140 0.000

.V5T12 0.700 0.049 14.244 0.000

.V5T13 0.646 0.118 5.501 0.000

.V5T7 ~~

.V5T8 0.759 0.037 20.782 0.000

.V5T9 0.689 0.041 16.848 0.000

.V5T10 0.705 0.047 14.978 0.000

.V5T11 0.640 0.047 13.599 0.000

.V5T12 0.646 0.052 12.375 0.000

.V5T13 0.609 0.130 4.692 0.000

.V5T8 ~~

.V5T9 0.759 0.035 21.450 0.000

.V5T10 0.756 0.041 18.601 0.000

.V5T11 0.688 0.044 15.798 0.000

.V5T12 0.698 0.048 14.399 0.000

.V5T13 0.554 0.128 4.328 0.000

.V5T9 ~~

.V5T10 0.731 0.039 18.963 0.000

.V5T11 0.612 0.044 13.982 0.000

.V5T12 0.626 0.047 13.345 0.000

.V5T13 0.518 0.116 4.457 0.000

.V5T10 ~~

.V5T11 0.712 0.037 19.145 0.000

.V5T12 0.723 0.042 17.203 0.000

.V5T13 0.728 0.112 6.498 0.000

.V5T11 ~~

.V5T12 0.716 0.038 18.917 0.000

.V5T13 0.631 0.110 5.732 0.000

.V5T12 ~~

.V5T13 0.620 0.117 5.317 0.000

.V6T1 ~~

.V6T2 0.639 0.023 27.829 0.000

.V6T3 0.584 0.027 21.660 0.000

.V6T4 0.476 0.034 14.029 0.000

.V6T5 0.479 0.035 13.799 0.000

.V6T6 0.523 0.038 13.810 0.000

.V6T7 0.494 0.043 11.405 0.000

.V6T8 0.466 0.044 10.554 0.000

.V6T9 0.396 0.046 8.681 0.000

.V6T10 0.444 0.047 9.494 0.000

.V6T11 0.412 0.048 8.588 0.000

.V6T12 0.497 0.051 9.828 0.000

.V6T13 0.624 0.129 4.848 0.000

.V6T2 ~~

.V6T3 0.600 0.025 23.784 0.000

.V6T4 0.530 0.031 17.205 0.000

.V6T5 0.508 0.035 14.654 0.000

.V6T6 0.569 0.036 15.654 0.000

.V6T7 0.530 0.041 13.063 0.000

.V6T8 0.579 0.042 13.886 0.000

.V6T9 0.467 0.044 10.573 0.000

.V6T10 0.598 0.049 12.174 0.000

.V6T11 0.524 0.050 10.537 0.000

.V6T12 0.556 0.048 11.474 0.000

.V6T13 0.644 0.131 4.902 0.000

.V6T3 ~~

.V6T4 0.566 0.028 20.353 0.000

.V6T5 0.523 0.032 16.505 0.000

.V6T6 0.545 0.033 16.364 0.000

.V6T7 0.569 0.035 16.042 0.000

.V6T8 0.585 0.035 16.486 0.000

.V6T9 0.502 0.040 12.450 0.000

.V6T10 0.546 0.046 11.815 0.000

.V6T11 0.541 0.044 12.193 0.000

.V6T12 0.492 0.047 10.427 0.000

.V6T13 0.566 0.100 5.656 0.000

.V6T4 ~~

.V6T5 0.591 0.028 20.957 0.000

.V6T6 0.595 0.033 18.219 0.000

.V6T7 0.614 0.034 18.050 0.000

.V6T8 0.565 0.038 15.022 0.000

.V6T9 0.457 0.040 11.322 0.000

.V6T10 0.590 0.041 14.391 0.000

.V6T11 0.491 0.047 10.512 0.000

.V6T12 0.578 0.047 12.332 0.000

.V6T13 0.614 0.116 5.311 0.000

.V6T5 ~~

.V6T6 0.623 0.029 21.850 0.000

.V6T7 0.588 0.034 17.061 0.000

.V6T8 0.570 0.038 14.817 0.000

.V6T9 0.456 0.041 10.998 0.000

.V6T10 0.584 0.043 13.532 0.000

.V6T11 0.485 0.045 10.845 0.000

.V6T12 0.557 0.044 12.786 0.000

.V6T13 0.526 0.103 5.110 0.000

.V6T6 ~~

.V6T7 0.624 0.029 21.525 0.000

.V6T8 0.618 0.034 18.343 0.000

.V6T9 0.522 0.039 13.530 0.000

.V6T10 0.591 0.041 14.319 0.000

.V6T11 0.489 0.045 10.813 0.000

.V6T12 0.576 0.043 13.522 0.000

.V6T13 0.605 0.115 5.274 0.000

.V6T7 ~~

.V6T8 0.670 0.031 21.960 0.000

.V6T9 0.525 0.036 14.383 0.000

.V6T10 0.591 0.039 15.308 0.000

.V6T11 0.573 0.041 13.864 0.000

.V6T12 0.561 0.044 12.761 0.000

.V6T13 0.501 0.108 4.650 0.000

.V6T8 ~~

.V6T9 0.646 0.030 21.276 0.000

.V6T10 0.583 0.038 15.262 0.000

.V6T11 0.556 0.040 14.054 0.000

.V6T12 0.549 0.040 13.583 0.000

.V6T13 0.584 0.096 6.092 0.000

.V6T9 ~~

.V6T10 0.598 0.035 17.190 0.000

.V6T11 0.473 0.039 12.239 0.000

.V6T12 0.493 0.042 11.663 0.000

.V6T13 0.600 0.098 6.136 0.000

.V6T10 ~~

.V6T11 0.600 0.035 17.198 0.000

.V6T12 0.633 0.037 17.180 0.000

.V6T13 0.702 0.090 7.842 0.000

.V6T11 ~~

.V6T12 0.600 0.035 17.315 0.000

.V6T13 0.553 0.093 5.912 0.000

.V6T12 ~~

.V6T13 0.614 0.092 6.706 0.000

.V7T1 ~~

.V7T2 0.715 0.022 32.961 0.000

.V7T3 0.658 0.025 25.962 0.000

.V7T4 0.679 0.031 21.715 0.000

.V7T5 0.620 0.035 17.558 0.000

.V7T6 0.642 0.040 16.095 0.000

.V7T7 0.648 0.041 15.829 0.000

.V7T8 0.615 0.045 13.620 0.000

.V7T9 0.635 0.045 14.265 0.000

.V7T10 0.607 0.048 12.746 0.000

.V7T11 0.623 0.051 12.282 0.000

.V7T12 0.612 0.052 11.820 0.000

.V7T13 0.742 0.125 5.939 0.000

.V7T2 ~~

.V7T3 0.655 0.024 27.288 0.000

.V7T4 0.640 0.028 23.099 0.000

.V7T5 0.615 0.031 20.050 0.000

.V7T6 0.554 0.038 14.600 0.000

.V7T7 0.664 0.038 17.425 0.000

.V7T8 0.570 0.043 13.301 0.000

.V7T9 0.550 0.045 12.333 0.000

.V7T10 0.561 0.047 11.829 0.000

.V7T11 0.492 0.052 9.547 0.000

.V7T12 0.524 0.050 10.582 0.000

.V7T13 0.407 0.138 2.943 0.003

.V7T3 ~~

.V7T4 0.710 0.026 27.535 0.000

.V7T5 0.647 0.030 21.896 0.000

.V7T6 0.592 0.038 15.750 0.000

.V7T7 0.613 0.037 16.674 0.000

.V7T8 0.596 0.040 14.882 0.000

.V7T9 0.633 0.041 15.635 0.000

.V7T10 0.631 0.043 14.805 0.000

.V7T11 0.589 0.044 13.531 0.000

.V7T12 0.598 0.047 12.804 0.000

.V7T13 0.696 0.110 6.301 0.000

.V7T4 ~~

.V7T5 0.677 0.027 24.764 0.000

.V7T6 0.674 0.034 19.988 0.000

.V7T7 0.656 0.036 18.378 0.000

.V7T8 0.653 0.039 16.636 0.000

.V7T9 0.572 0.042 13.588 0.000

.V7T10 0.610 0.044 13.747 0.000

.V7T11 0.571 0.046 12.461 0.000

.V7T12 0.613 0.046 13.460 0.000

.V7T13 0.440 0.117 3.775 0.000

.V7T5 ~~

.V7T6 0.657 0.030 22.212 0.000

.V7T7 0.689 0.036 19.139 0.000

.V7T8 0.599 0.038 15.770 0.000

.V7T9 0.658 0.041 16.044 0.000

.V7T10 0.650 0.043 15.158 0.000

.V7T11 0.672 0.043 15.543 0.000

.V7T12 0.631 0.047 13.513 0.000

.V7T13 0.629 0.108 5.810 0.000

.V7T6 ~~

.V7T7 0.705 0.031 22.383 0.000

.V7T8 0.705 0.035 20.330 0.000

.V7T9 0.700 0.038 18.257 0.000

.V7T10 0.616 0.044 13.994 0.000

.V7T11 0.734 0.041 17.868 0.000

.V7T12 0.683 0.042 16.180 0.000

.V7T13 0.364 0.122 2.985 0.003

.V7T7 ~~

.V7T8 0.675 0.029 22.910 0.000

.V7T9 0.755 0.034 21.972 0.000

.V7T10 0.720 0.039 18.444 0.000

.V7T11 0.710 0.039 18.238 0.000

.V7T12 0.723 0.040 18.221 0.000

.V7T13 0.682 0.101 6.783 0.000

.V7T8 ~~

.V7T9 0.756 0.030 25.189 0.000

.V7T10 0.664 0.036 18.693 0.000

.V7T11 0.675 0.038 17.571 0.000

.V7T12 0.617 0.041 15.157 0.000

.V7T13 0.625 0.101 6.179 0.000

.V7T9 ~~

.V7T10 0.735 0.033 22.477 0.000

.V7T11 0.683 0.037 18.349 0.000

.V7T12 0.655 0.039 16.724 0.000

.V7T13 0.538 0.095 5.682 0.000

.V7T10 ~~

.V7T11 0.756 0.031 24.715 0.000

.V7T12 0.736 0.033 22.605 0.000

.V7T13 0.554 0.098 5.633 0.000

.V7T11 ~~

.V7T12 0.770 0.028 27.187 0.000

.V7T13 0.605 0.111 5.449 0.000

.V7T12 ~~

.V7T13 0.447 0.115 3.884 0.000

Intercepts:

Estimate Std.Err z-value P(>|z|)

G1 0.000

G2 -0.225 0.021 -10.493 0.000

G3 -0.481 0.027 -17.815 0.000

G4 -0.650 0.031 -21.003 0.000

G5 -0.765 0.035 -21.871 0.000

G6 -0.853 0.038 -22.208 0.000

G7 -0.982 0.041 -24.052 0.000

G8 -1.021 0.043 -23.725 0.000

G9 -1.070 0.045 -23.637 0.000

G10 -1.175 0.048 -24.535 0.000

G11 -1.230 0.051 -24.319 0.000

G12 -1.394 0.053 -26.187 0.000

G13 -1.665 0.121 -13.765 0.000

.V1T1 0.000

.V2T1 0.000

.V3T1 0.000

.V4T1 0.000

.V5T1 0.000

.V6T1 0.000

.V7T1 0.000

.V1T2 0.000

.V2T2 0.000

.V3T2 0.000

.V4T2 0.000

.V5T2 0.000

.V6T2 0.000

.V7T2 0.000

.V1T3 0.000

.V2T3 0.000

.V3T3 0.000

.V4T3 0.000

.V5T3 0.000

.V6T3 0.000

.V7T3 0.000

.V1T4 0.000

.V2T4 0.000

.V3T4 0.000

.V4T4 0.000

.V5T4 0.000

.V6T4 0.000

.V7T4 0.000

.V1T5 0.000

.V2T5 0.000

.V3T5 0.000

.V4T5 0.000

.V5T5 0.000

.V6T5 0.000

.V7T5 0.000

.V1T6 0.000

.V2T6 0.000

.V3T6 0.000

.V4T6 0.000

.V5T6 0.000

.V6T6 0.000

.V7T6 0.000

.V1T7 0.000

.V2T7 0.000

.V3T7 0.000

.V4T7 0.000

.V5T7 0.000

.V6T7 0.000

.V7T7 0.000

.V1T8 0.000

.V2T8 0.000

.V3T8 0.000

.V4T8 0.000

.V5T8 0.000

.V6T8 0.000

.V7T8 0.000

.V1T9 0.000

.V2T9 0.000

.V3T9 0.000

.V4T9 0.000

.V5T9 0.000

.V6T9 0.000

.V7T9 0.000

.V1T10 0.000

.V2T10 0.000

.V3T10 0.000

.V4T10 0.000

.V5T10 0.000

.V6T10 0.000

.V7T10 0.000

.V1T11 0.000

.V2T11 0.000

.V3T11 0.000

.V4T11 0.000

.V5T11 0.000

.V6T11 0.000

.V7T11 0.000

.V1T12 0.000

.V2T12 0.000

.V3T12 0.000

.V4T12 0.000

.V5T12 0.000

.V6T12 0.000

.V7T12 0.000

.V1T13 0.000

.V2T13 0.000

.V3T13 0.000

.V4T13 0.000

.V5T13 0.000

.V6T13 0.000

.V7T13 0.000

Thresholds:

Estimate Std.Err z-value P(>|z|)

V1T1|t1 (t1_1) -3.582 0.070 -51.236 0.000

V1T1|t2 (t1_2) -0.446 0.034 -13.190 0.000

V1T1|t3 (t1_3) 0.681 0.033 20.409 0.000

V1T2|t1 (t1_1) -3.582 0.070 -51.236 0.000

V1T2|t2 (t1_2) -0.446 0.034 -13.190 0.000

V1T2|t3 (t1_3) 0.681 0.033 20.409 0.000

V1T3|t1 (t1_1) -3.582 0.070 -51.236 0.000

V1T3|t2 (t1_2) -0.446 0.034 -13.190 0.000

V1T3|t3 (t1_3) 0.681 0.033 20.409 0.000

V1T4|t1 (t1_1) -3.582 0.070 -51.236 0.000

V1T4|t2 (t1_2) -0.446 0.034 -13.190 0.000

V1T4|t3 (t1_3) 0.681 0.033 20.409 0.000

V1T5|t1 (t1_1) -3.582 0.070 -51.236 0.000

V1T5|t2 (t1_2) -0.446 0.034 -13.190 0.000

V1T5|t3 (t1_3) 0.681 0.033 20.409 0.000

V1T6|t1 (t1_1) -3.582 0.070 -51.236 0.000

V1T6|t2 (t1_2) -0.446 0.034 -13.190 0.000

V1T6|t3 (t1_3) 0.681 0.033 20.409 0.000

V1T7|t1 (t1_1) -3.582 0.070 -51.236 0.000

V1T7|t2 (t1_2) -0.446 0.034 -13.190 0.000

V1T7|t3 (t1_3) 0.681 0.033 20.409 0.000

V1T8|t1 (t1_1) -3.582 0.070 -51.236 0.000

V1T8|t2 (t1_2) -0.446 0.034 -13.190 0.000

V1T8|t3 (t1_3) 0.681 0.033 20.409 0.000

V1T9|t1 (t1_1) -3.582 0.070 -51.236 0.000

V1T9|t2 (t1_2) -0.446 0.034 -13.190 0.000

V1T9|t3 (t1_3) 0.681 0.033 20.409 0.000

V1T10|1 (t1_1) -3.582 0.070 -51.236 0.000

V1T10|2 (t1_2) -0.446 0.034 -13.190 0.000

V1T10|3 (t1_3) 0.681 0.033 20.409 0.000

V1T11|1 (t1_1) -3.582 0.070 -51.236 0.000

V1T11|2 (t1_2) -0.446 0.034 -13.190 0.000

V1T11|3 (t1_3) 0.681 0.033 20.409 0.000

V1T12|1 (t1_1) -3.582 0.070 -51.236 0.000

V1T12|2 (t1_2) -0.446 0.034 -13.190 0.000

V1T12|3 (t1_3) 0.681 0.033 20.409 0.000

V1T13|1 (t1_1) -3.582 0.070 -51.236 0.000

V1T13|2 (t1_2) -0.446 0.034 -13.190 0.000

V1T13|3 (t1_3) 0.681 0.033 20.409 0.000

V2T1|t1 (t2_1) -2.961 0.074 -40.244 0.000

V2T1|t2 (t2_2) -0.098 0.039 -2.525 0.012

V2T1|t3 (t2_3) 1.309 0.041 31.664 0.000

V2T2|t1 (t2_1) -2.961 0.074 -40.244 0.000

V2T2|t2 (t2_2) -0.098 0.039 -2.525 0.012

V2T2|t3 (t2_3) 1.309 0.041 31.664 0.000

V2T3|t1 (t2_1) -2.961 0.074 -40.244 0.000

V2T3|t2 (t2_2) -0.098 0.039 -2.525 0.012

V2T3|t3 (t2_3) 1.309 0.041 31.664 0.000

V2T4|t1 (t2_1) -2.961 0.074 -40.244 0.000

V2T4|t2 (t2_2) -0.098 0.039 -2.525 0.012

V2T4|t3 (t2_3) 1.309 0.041 31.664 0.000

V2T5|t1 (t2_1) -2.961 0.074 -40.244 0.000

V2T5|t2 (t2_2) -0.098 0.039 -2.525 0.012

V2T5|t3 (t2_3) 1.309 0.041 31.664 0.000

V2T6|t1 (t2_1) -2.961 0.074 -40.244 0.000

V2T6|t2 (t2_2) -0.098 0.039 -2.525 0.012

V2T6|t3 (t2_3) 1.309 0.041 31.664 0.000

V2T7|t1 (t2_1) -2.961 0.074 -40.244 0.000

V2T7|t2 (t2_2) -0.098 0.039 -2.525 0.012

V2T7|t3 (t2_3) 1.309 0.041 31.664 0.000

V2T8|t1 (t2_1) -2.961 0.074 -40.244 0.000

V2T8|t2 (t2_2) -0.098 0.039 -2.525 0.012

V2T8|t3 (t2_3) 1.309 0.041 31.664 0.000

V2T9|t1 (t2_1) -2.961 0.074 -40.244 0.000

V2T9|t2 (t2_2) -0.098 0.039 -2.525 0.012

V2T9|t3 (t2_3) 1.309 0.041 31.664 0.000

V2T10|1 (t2_1) -2.961 0.074 -40.244 0.000

V2T10|2 (t2_2) -0.098 0.039 -2.525 0.012

V2T10|3 (t2_3) 1.309 0.041 31.664 0.000

V2T11|1 (t2_1) -2.961 0.074 -40.244 0.000

V2T11|2 (t2_2) -0.098 0.039 -2.525 0.012

V2T11|3 (t2_3) 1.309 0.041 31.664 0.000

V2T12|1 (t2_1) -2.961 0.074 -40.244 0.000

V2T12|2 (t2_2) -0.098 0.039 -2.525 0.012

V2T12|3 (t2_3) 1.309 0.041 31.664 0.000

V2T13|1 (t2_1) -2.961 0.074 -40.244 0.000

V2T13|2 (t2_2) -0.098 0.039 -2.525 0.012

V2T13|3 (t2_3) 1.309 0.041 31.664 0.000

V3T1|t1 (t3_1) -3.140 0.067 -46.750 0.000

V3T1|t2 (t3_2) -0.244 0.039 -6.320 0.000

V3T1|t3 (t3_3) 1.134 0.040 28.439 0.000

V3T2|t1 (t3_1) -3.140 0.067 -46.750 0.000

V3T2|t2 (t3_2) -0.244 0.039 -6.320 0.000

V3T2|t3 (t3_3) 1.134 0.040 28.439 0.000

V3T3|t1 (t3_1) -3.140 0.067 -46.750 0.000

V3T3|t2 (t3_2) -0.244 0.039 -6.320 0.000

V3T3|t3 (t3_3) 1.134 0.040 28.439 0.000

V3T4|t1 (t3_1) -3.140 0.067 -46.750 0.000

V3T4|t2 (t3_2) -0.244 0.039 -6.320 0.000

V3T4|t3 (t3_3) 1.134 0.040 28.439 0.000

V3T5|t1 (t3_1) -3.140 0.067 -46.750 0.000

V3T5|t2 (t3_2) -0.244 0.039 -6.320 0.000

V3T5|t3 (t3_3) 1.134 0.040 28.439 0.000

V3T6|t1 (t3_1) -3.140 0.067 -46.750 0.000

V3T6|t2 (t3_2) -0.244 0.039 -6.320 0.000

V3T6|t3 (t3_3) 1.134 0.040 28.439 0.000

V3T7|t1 (t3_1) -3.140 0.067 -46.750 0.000

V3T7|t2 (t3_2) -0.244 0.039 -6.320 0.000

V3T7|t3 (t3_3) 1.134 0.040 28.439 0.000

V3T8|t1 (t3_1) -3.140 0.067 -46.750 0.000

V3T8|t2 (t3_2) -0.244 0.039 -6.320 0.000

V3T8|t3 (t3_3) 1.134 0.040 28.439 0.000

V3T9|t1 (t3_1) -3.140 0.067 -46.750 0.000

V3T9|t2 (t3_2) -0.244 0.039 -6.320 0.000

V3T9|t3 (t3_3) 1.134 0.040 28.439 0.000

V3T10|1 (t3_1) -3.140 0.067 -46.750 0.000

V3T10|2 (t3_2) -0.244 0.039 -6.320 0.000

V3T10|3 (t3_3) 1.134 0.040 28.439 0.000

V3T11|1 (t3_1) -3.140 0.067 -46.750 0.000

V3T11|2 (t3_2) -0.244 0.039 -6.320 0.000

V3T11|3 (t3_3) 1.134 0.040 28.439 0.000

V3T12|1 (t3_1) -3.140 0.067 -46.750 0.000

V3T12|2 (t3_2) -0.244 0.039 -6.320 0.000

V3T12|3 (t3_3) 1.134 0.040 28.439 0.000

V3T13|1 (t3_1) -3.140 0.067 -46.750 0.000

V3T13|2 (t3_2) -0.244 0.039 -6.320 0.000

V3T13|3 (t3_3) 1.134 0.040 28.439 0.000

V4T1|t1 (t4_1) -2.326 0.053 -44.272 0.000

V4T1|t2 (t4_2) -0.079 0.031 -2.525 0.012

V4T1|t3 (t4_3) 0.971 0.033 29.367 0.000

V4T2|t1 (t4_1) -2.326 0.053 -44.272 0.000

V4T2|t2 (t4_2) -0.079 0.031 -2.525 0.012

V4T2|t3 (t4_3) 0.971 0.033 29.367 0.000

V4T3|t1 (t4_1) -2.326 0.053 -44.272 0.000

V4T3|t2 (t4_2) -0.079 0.031 -2.525 0.012

V4T3|t3 (t4_3) 0.971 0.033 29.367 0.000

V4T4|t1 (t4_1) -2.326 0.053 -44.272 0.000

V4T4|t2 (t4_2) -0.079 0.031 -2.525 0.012

V4T4|t3 (t4_3) 0.971 0.033 29.367 0.000

V4T5|t1 (t4_1) -2.326 0.053 -44.272 0.000

V4T5|t2 (t4_2) -0.079 0.031 -2.525 0.012

V4T5|t3 (t4_3) 0.971 0.033 29.367 0.000

V4T6|t1 (t4_1) -2.326 0.053 -44.272 0.000

V4T6|t2 (t4_2) -0.079 0.031 -2.525 0.012

V4T6|t3 (t4_3) 0.971 0.033 29.367 0.000

V4T7|t1 (t4_1) -2.326 0.053 -44.272 0.000

V4T7|t2 (t4_2) -0.079 0.031 -2.525 0.012

V4T7|t3 (t4_3) 0.971 0.033 29.367 0.000

V4T8|t1 (t4_1) -2.326 0.053 -44.272 0.000

V4T8|t2 (t4_2) -0.079 0.031 -2.525 0.012

V4T8|t3 (t4_3) 0.971 0.033 29.367 0.000

V4T9|t1 (t4_1) -2.326 0.053 -44.272 0.000

V4T9|t2 (t4_2) -0.079 0.031 -2.525 0.012

V4T9|t3 (t4_3) 0.971 0.033 29.367 0.000

V4T10|1 (t4_1) -2.326 0.053 -44.272 0.000

V4T10|2 (t4_2) -0.079 0.031 -2.525 0.012

V4T10|3 (t4_3) 0.971 0.033 29.367 0.000

V4T11|1 (t4_1) -2.326 0.053 -44.272 0.000

V4T11|2 (t4_2) -0.079 0.031 -2.525 0.012

V4T11|3 (t4_3) 0.971 0.033 29.367 0.000

V4T12|1 (t4_1) -2.326 0.053 -44.272 0.000

V4T12|2 (t4_2) -0.079 0.031 -2.525 0.012

V4T12|3 (t4_3) 0.971 0.033 29.367 0.000

V4T13|1 (t4_1) -2.326 0.053 -44.272 0.000

V4T13|2 (t4_2) -0.079 0.031 -2.525 0.012

V4T13|3 (t4_3) 0.971 0.033 29.367 0.000

V5T1|t1 (t5_1) -0.759 0.041 -18.663 0.000

V5T1|t2 (t5_2) 0.865 0.030 28.805 0.000

V5T1|t3 (t5_3) 1.760 0.039 45.256 0.000

V5T2|t1 (t5_1) -0.759 0.041 -18.663 0.000

V5T2|t2 (t5_2) 0.865 0.030 28.805 0.000

V5T2|t3 (t5_3) 1.760 0.039 45.256 0.000

V5T3|t1 (t5_1) -0.759 0.041 -18.663 0.000

V5T3|t2 (t5_2) 0.865 0.030 28.805 0.000

V5T3|t3 (t5_3) 1.760 0.039 45.256 0.000

V5T4|t1 (t5_1) -0.759 0.041 -18.663 0.000

V5T4|t2 (t5_2) 0.865 0.030 28.805 0.000

V5T4|t3 (t5_3) 1.760 0.039 45.256 0.000

V5T5|t1 (t5_1) -0.759 0.041 -18.663 0.000

V5T5|t2 (t5_2) 0.865 0.030 28.805 0.000

V5T5|t3 (t5_3) 1.760 0.039 45.256 0.000

V5T6|t1 (t5_1) -0.759 0.041 -18.663 0.000

V5T6|t2 (t5_2) 0.865 0.030 28.805 0.000

V5T6|t3 (t5_3) 1.760 0.039 45.256 0.000

V5T7|t1 (t5_1) -0.759 0.041 -18.663 0.000

V5T7|t2 (t5_2) 0.865 0.030 28.805 0.000

V5T7|t3 (t5_3) 1.760 0.039 45.256 0.000

V5T8|t1 (t5_1) -0.759 0.041 -18.663 0.000

V5T8|t2 (t5_2) 0.865 0.030 28.805 0.000

V5T8|t3 (t5_3) 1.760 0.039 45.256 0.000

V5T9|t1 (t5_1) -0.759 0.041 -18.663 0.000

V5T9|t2 (t5_2) 0.865 0.030 28.805 0.000

V5T9|t3 (t5_3) 1.760 0.039 45.256 0.000

V5T10|1 (t5_1) -0.759 0.041 -18.663 0.000

V5T10|2 (t5_2) 0.865 0.030 28.805 0.000

V5T10|3 (t5_3) 1.760 0.039 45.256 0.000

V5T11|1 (t5_1) -0.759 0.041 -18.663 0.000

V5T11|2 (t5_2) 0.865 0.030 28.805 0.000

V5T11|3 (t5_3) 1.760 0.039 45.256 0.000

V5T12|1 (t5_1) -0.759 0.041 -18.663 0.000

V5T12|2 (t5_2) 0.865 0.030 28.805 0.000

V5T12|3 (t5_3) 1.760 0.039 45.256 0.000

V5T13|1 (t5_1) -0.759 0.041 -18.663 0.000

V5T13|2 (t5_2) 0.865 0.030 28.805 0.000

V5T13|3 (t5_3) 1.760 0.039 45.256 0.000

V6T1|t1 (t6_1) -1.766 0.045 -38.838 0.000

V6T1|t2 (t6_2) 0.126 0.028 4.524 0.000

V6T1|t3 (t6_3) 0.999 0.030 33.463 0.000

V6T2|t1 (t6_1) -1.766 0.045 -38.838 0.000

V6T2|t2 (t6_2) 0.126 0.028 4.524 0.000

V6T2|t3 (t6_3) 0.999 0.030 33.463 0.000

V6T3|t1 (t6_1) -1.766 0.045 -38.838 0.000

V6T3|t2 (t6_2) 0.126 0.028 4.524 0.000

V6T3|t3 (t6_3) 0.999 0.030 33.463 0.000

V6T4|t1 (t6_1) -1.766 0.045 -38.838 0.000

V6T4|t2 (t6_2) 0.126 0.028 4.524 0.000

V6T4|t3 (t6_3) 0.999 0.030 33.463 0.000

V6T5|t1 (t6_1) -1.766 0.045 -38.838 0.000

V6T5|t2 (t6_2) 0.126 0.028 4.524 0.000

V6T5|t3 (t6_3) 0.999 0.030 33.463 0.000

V6T6|t1 (t6_1) -1.766 0.045 -38.838 0.000

V6T6|t2 (t6_2) 0.126 0.028 4.524 0.000

V6T6|t3 (t6_3) 0.999 0.030 33.463 0.000

V6T7|t1 (t6_1) -1.766 0.045 -38.838 0.000

V6T7|t2 (t6_2) 0.126 0.028 4.524 0.000

V6T7|t3 (t6_3) 0.999 0.030 33.463 0.000

V6T8|t1 (t6_1) -1.766 0.045 -38.838 0.000

V6T8|t2 (t6_2) 0.126 0.028 4.524 0.000

V6T8|t3 (t6_3) 0.999 0.030 33.463 0.000

V6T9|t1 (t6_1) -1.766 0.045 -38.838 0.000

V6T9|t2 (t6_2) 0.126 0.028 4.524 0.000

V6T9|t3 (t6_3) 0.999 0.030 33.463 0.000

V6T10|1 (t6_1) -1.766 0.045 -38.838 0.000

V6T10|2 (t6_2) 0.126 0.028 4.524 0.000

V6T10|3 (t6_3) 0.999 0.030 33.463 0.000

V6T11|1 (t6_1) -1.766 0.045 -38.838 0.000

V6T11|2 (t6_2) 0.126 0.028 4.524 0.000

V6T11|3 (t6_3) 0.999 0.030 33.463 0.000

V6T12|1 (t6_1) -1.766 0.045 -38.838 0.000

V6T12|2 (t6_2) 0.126 0.028 4.524 0.000

V6T12|3 (t6_3) 0.999 0.030 33.463 0.000

V6T13|1 (t6_1) -1.766 0.045 -38.838 0.000

V6T13|2 (t6_2) 0.126 0.028 4.524 0.000

V6T13|3 (t6_3) 0.999 0.030 33.463 0.000

V7T1|t1 (t7_1) -1.457 0.044 -32.822 0.000

V7T1|t2 (t7_2) 0.279 0.030 9.172 0.000

V7T1|t3 (t7_3) 1.052 0.032 33.049 0.000

V7T2|t1 (t7_1) -1.457 0.044 -32.822 0.000

V7T2|t2 (t7_2) 0.279 0.030 9.172 0.000

V7T2|t3 (t7_3) 1.052 0.032 33.049 0.000

V7T3|t1 (t7_1) -1.457 0.044 -32.822 0.000

V7T3|t2 (t7_2) 0.279 0.030 9.172 0.000

V7T3|t3 (t7_3) 1.052 0.032 33.049 0.000

V7T4|t1 (t7_1) -1.457 0.044 -32.822 0.000

V7T4|t2 (t7_2) 0.279 0.030 9.172 0.000

V7T4|t3 (t7_3) 1.052 0.032 33.049 0.000

V7T5|t1 (t7_1) -1.457 0.044 -32.822 0.000

V7T5|t2 (t7_2) 0.279 0.030 9.172 0.000

V7T5|t3 (t7_3) 1.052 0.032 33.049 0.000

V7T6|t1 (t7_1) -1.457 0.044 -32.822 0.000

V7T6|t2 (t7_2) 0.279 0.030 9.172 0.000

V7T6|t3 (t7_3) 1.052 0.032 33.049 0.000

V7T7|t1 (t7_1) -1.457 0.044 -32.822 0.000

V7T7|t2 (t7_2) 0.279 0.030 9.172 0.000

V7T7|t3 (t7_3) 1.052 0.032 33.049 0.000

V7T8|t1 (t7_1) -1.457 0.044 -32.822 0.000

V7T8|t2 (t7_2) 0.279 0.030 9.172 0.000

V7T8|t3 (t7_3) 1.052 0.032 33.049 0.000

V7T9|t1 (t7_1) -1.457 0.044 -32.822 0.000

V7T9|t2 (t7_2) 0.279 0.030 9.172 0.000

V7T9|t3 (t7_3) 1.052 0.032 33.049 0.000

V7T10|1 (t7_1) -1.457 0.044 -32.822 0.000

V7T10|2 (t7_2) 0.279 0.030 9.172 0.000

V7T10|3 (t7_3) 1.052 0.032 33.049 0.000

V7T11|1 (t7_1) -1.457 0.044 -32.822 0.000

V7T11|2 (t7_2) 0.279 0.030 9.172 0.000

V7T11|3 (t7_3) 1.052 0.032 33.049 0.000

V7T12|1 (t7_1) -1.457 0.044 -32.822 0.000

V7T12|2 (t7_2) 0.279 0.030 9.172 0.000

V7T12|3 (t7_3) 1.052 0.032 33.049 0.000

V7T13|1 (t7_1) -1.457 0.044 -32.822 0.000

V7T13|2 (t7_2) 0.279 0.030 9.172 0.000

V7T13|3 (t7_3) 1.052 0.032 33.049 0.000

Variances:

Estimate Std.Err z-value P(>|z|)

G1 1.000

G2 1.216 0.047 25.734 0.000

G3 1.316 0.057 23.095 0.000

G4 1.462 0.067 21.710 0.000

G5 1.564 0.077 20.319 0.000

G6 1.628 0.084 19.463 0.000

G7 1.682 0.090 18.741 0.000

G8 1.666 0.096 17.375 0.000

G9 1.660 0.098 16.877 0.000

G10 1.763 0.108 16.294 0.000

G11 1.876 0.115 16.321 0.000

G12 1.920 0.118 16.254 0.000

G13 2.697 0.345 7.819 0.000

.V1T1 1.000

.V2T1 1.000

.V3T1 1.000

.V4T1 1.000

.V5T1 1.000

.V6T1 1.000

.V7T1 1.000

.V1T2 1.000

.V2T2 1.000

.V3T2 1.000

.V4T2 1.000

.V5T2 1.000

.V6T2 1.000

.V7T2 1.000

.V1T3 1.000

.V2T3 1.000

.V3T3 1.000

.V4T3 1.000

.V5T3 1.000

.V6T3 1.000

.V7T3 1.000

.V1T4 1.000

.V2T4 1.000

.V3T4 1.000

.V4T4 1.000

.V5T4 1.000

.V6T4 1.000

.V7T4 1.000

.V1T5 1.000

.V2T5 1.000

.V3T5 1.000

.V4T5 1.000

.V5T5 1.000

.V6T5 1.000

.V7T5 1.000

.V1T6 1.000

.V2T6 1.000

.V3T6 1.000

.V4T6 1.000

.V5T6 1.000

.V6T6 1.000

.V7T6 1.000

.V1T7 1.000

.V2T7 1.000

.V3T7 1.000

.V4T7 1.000

.V5T7 1.000

.V6T7 1.000

.V7T7 1.000

.V1T8 1.000

.V2T8 1.000

.V3T8 1.000

.V4T8 1.000

.V5T8 1.000

.V6T8 1.000

.V7T8 1.000

.V1T9 1.000

.V2T9 1.000

.V3T9 1.000

.V4T9 1.000

.V5T9 1.000

.V6T9 1.000

.V7T9 1.000

.V1T10 1.000

.V2T10 1.000

.V3T10 1.000

.V4T10 1.000

.V5T10 1.000

.V6T10 1.000

.V7T10 1.000

.V1T11 1.000

.V2T11 1.000

.V3T11 1.000

.V4T11 1.000

.V5T11 1.000

.V6T11 1.000

.V7T11 1.000

.V1T12 1.000

.V2T12 1.000

.V3T12 1.000

.V4T12 1.000

.V5T12 1.000

.V6T12 1.000

.V7T12 1.000

.V1T13 1.000

.V2T13 1.000

.V3T13 1.000

.V4T13 1.000

.V5T13 1.000

.V6T13 1.000

.V7T13 1.000

Scales y*:

Estimate Std.Err z-value P(>|z|)

V1T1 0.653

V2T1 0.560

V3T1 0.575

V4T1 0.691

V5T1 0.750

V6T1 0.792

V7T1 0.763

V1T2 0.615

V2T2 0.522

V3T2 0.538

V4T2 0.655

V5T2 0.717

V6T2 0.762

V7T2 0.731

V1T3 0.600

V2T3 0.508

V3T3 0.523

V4T3 0.640

V5T3 0.703

V6T3 0.749

V7T3 0.718

V1T4 0.580

V2T4 0.488

V3T4 0.503

V4T4 0.620

V5T4 0.684

V6T4 0.732

V7T4 0.699

V1T5 0.567

V2T5 0.475

V3T5 0.490

V4T5 0.607

V5T5 0.672

V6T5 0.720

V7T5 0.687

V1T6 0.559

V2T6 0.468

V3T6 0.483

V4T6 0.599

V5T6 0.665

V6T6 0.713

V7T6 0.679

V1T7 0.553

V2T7 0.462

V3T7 0.477

V4T7 0.593

V5T7 0.658

V6T7 0.708

V7T7 0.673

V1T8 0.555

V2T8 0.464

V3T8 0.478

V4T8 0.595

V5T8 0.660

V6T8 0.709

V7T8 0.675

V1T9 0.556

V2T9 0.464

V3T9 0.479

V4T9 0.596

V5T9 0.661

V6T9 0.710

V7T9 0.676

V1T10 0.544

V2T10 0.453

V3T10 0.468

V4T10 0.584

V5T10 0.650

V6T10 0.699

V7T10 0.665

V1T11 0.532

V2T11 0.442

V3T11 0.457

V4T11 0.572

V5T11 0.638

V6T11 0.688

V7T11 0.653

V1T12 0.528

V2T12 0.438

V3T12 0.452

V4T12 0.568

V5T12 0.634

V6T12 0.684

V7T12 0.649

V1T13 0.464

V2T13 0.380

V3T13 0.394

V4T13 0.503

V5T13 0.568

V6T13 0.620

V7T13 0.584

### Online psychotherapy for depressive disorders

#### Configural invariance

lavaan 0.6-7 ended normally after 113 iterations

Estimator DWLS

Optimization method NLMINB

Number of free parameters 451

Number of equality constraints 239

Number of observations 3922

Number of missing patterns 5

Model Test User Model:

Standard Robust

Test Statistic 20790.830 11255.863

Degrees of freedom 3187 3187

P-value (Chi-square) 0.000 0.000

Scaling correction factor 2.274

Shift parameter 2111.057

simple second-order correction (WLSMV)

Model Test Baseline Model:

Test statistic 736892.767 135578.514

Degrees of freedom 3160 3160

P-value 0.000 0.000

Scaling correction factor 5.541

User Model versus Baseline Model:

Comparative Fit Index (CFI) 0.976 0.939

Tucker-Lewis Index (TLI) 0.976 0.940

Robust Comparative Fit Index (CFI) NA

Robust Tucker-Lewis Index (TLI) NA

Root Mean Square Error of Approximation:

RMSEA 0.038 0.025

90 Percent confidence interval - lower 0.037 0.025

90 Percent confidence interval - upper 0.038 0.026

P-value RMSEA <= 0.05 1.000 1.000

Robust RMSEA NA

90 Percent confidence interval - lower NA

90 Percent confidence interval - upper NA

Standardized Root Mean Square Residual:

SRMR 0.070 0.070

Weighted Root Mean Square Residual:

WRMR 2.473 2.473

Parameter Estimates:

Standard errors Robust.sem

Information Expected

Information saturated (h1) model Unstructured

Latent Variables:

Estimate Std.Err z-value P(>|z|)

G1 =~

V1T1 (lmb1) 0.915 0.025 37.304 0.000

V2T1 (lmb2) 0.875 0.022 39.087 0.000

V3T1 (lmb3) 1.011 0.026 38.402 0.000

V4T1 (lmb4) 0.882 0.022 39.615 0.000

V5T1 (lmb5) 1.182 0.029 40.291 0.000

V6T1 (lmb6) 0.608 0.023 26.532 0.000

V7T1 (lmb7) 1.049 0.028 37.659 0.000

V8T1 (lmb8) 0.799 0.021 37.930 0.000

V9T1 (lmb9) 0.651 0.025 25.724 0.000

V10T1 (lm10) 0.485 0.019 25.204 0.000

V11T1 (lm11) 0.623 0.017 35.792 0.000

V12T1 (lm12) 0.738 0.022 33.500 0.000

V13T1 (lm13) 0.754 0.023 32.213 0.000

V14T1 (lm14) 0.627 0.023 27.317 0.000

V15T1 (lm15) 0.797 0.023 34.487 0.000

V16T1 (lm16) 0.442 0.018 23.969 0.000

V17T1 (lm17) 0.774 0.021 36.010 0.000

V18T1 (lm18) 0.502 0.023 21.711 0.000

V19T1 (lm19) 0.262 0.026 10.240 0.000

V20T1 (lm20) 0.382 0.019 20.669 0.000

G3 =~

V1T3 (lmb1) 0.915 0.025 37.304 0.000

V2T3 (lmb2) 0.875 0.022 39.087 0.000

V3T3 (lmb3) 1.011 0.026 38.402 0.000

V4T3 (lmb4) 0.882 0.022 39.615 0.000

V5T3 (lmb5) 1.182 0.029 40.291 0.000

V6T3 (lmb6) 0.608 0.023 26.532 0.000

V7T3 (lmb7) 1.049 0.028 37.659 0.000

V8T3 (lmb8) 0.799 0.021 37.930 0.000

V9T3 (lmb9) 0.651 0.025 25.724 0.000

V10T3 (lm10) 0.485 0.019 25.204 0.000

V11T3 (lm11) 0.623 0.017 35.792 0.000

V12T3 (lm12) 0.738 0.022 33.500 0.000

V13T3 (lm13) 0.754 0.023 32.213 0.000

V14T3 (lm14) 0.627 0.023 27.317 0.000

V15T3 (lm15) 0.797 0.023 34.487 0.000

V16T3 (lm16) 0.442 0.018 23.969 0.000

V17T3 (lm17) 0.774 0.021 36.010 0.000

V18T3 (lm18) 0.502 0.023 21.711 0.000

V19T3 (lm19) 0.262 0.026 10.240 0.000

V20T3 (lm20) 0.382 0.019 20.669 0.000

G7 =~

V1T7 (lmb1) 0.915 0.025 37.304 0.000

V2T7 (lmb2) 0.875 0.022 39.087 0.000

V3T7 (lmb3) 1.011 0.026 38.402 0.000

V4T7 (lmb4) 0.882 0.022 39.615 0.000

V5T7 (lmb5) 1.182 0.029 40.291 0.000

V6T7 (lmb6) 0.608 0.023 26.532 0.000

V7T7 (lmb7) 1.049 0.028 37.659 0.000

V8T7 (lmb8) 0.799 0.021 37.930 0.000

V9T7 (lmb9) 0.651 0.025 25.724 0.000

V10T7 (lm10) 0.485 0.019 25.204 0.000

V11T7 (lm11) 0.623 0.017 35.792 0.000

V12T7 (lm12) 0.738 0.022 33.500 0.000

V13T7 (lm13) 0.754 0.023 32.213 0.000

V14T7 (lm14) 0.627 0.023 27.317 0.000

V15T7 (lm15) 0.797 0.023 34.487 0.000

V16T7 (lm16) 0.442 0.018 23.969 0.000

V17T7 (lm17) 0.774 0.021 36.010 0.000

V18T7 (lm18) 0.502 0.023 21.711 0.000

V19T7 (lm19) 0.262 0.026 10.240 0.000

V20T7 (lm20) 0.382 0.019 20.669 0.000

G8 =~

V1T8 (lmb1) 0.915 0.025 37.304 0.000

V2T8 (lmb2) 0.875 0.022 39.087 0.000

V3T8 (lmb3) 1.011 0.026 38.402 0.000

V4T8 (lmb4) 0.882 0.022 39.615 0.000

V5T8 (lmb5) 1.182 0.029 40.291 0.000

V6T8 (lmb6) 0.608 0.023 26.532 0.000

V7T8 (lmb7) 1.049 0.028 37.659 0.000

V8T8 (lmb8) 0.799 0.021 37.930 0.000

V9T8 (lmb9) 0.651 0.025 25.724 0.000

V10T8 (lm10) 0.485 0.019 25.204 0.000

V11T8 (lm11) 0.623 0.017 35.792 0.000

V12T8 (lm12) 0.738 0.022 33.500 0.000

V13T8 (lm13) 0.754 0.023 32.213 0.000

V14T8 (lm14) 0.627 0.023 27.317 0.000

V15T8 (lm15) 0.797 0.023 34.487 0.000

V16T8 (lm16) 0.442 0.018 23.969 0.000

V17T8 (lm17) 0.774 0.021 36.010 0.000

V18T8 (lm18) 0.502 0.023 21.711 0.000

V19T8 (lm19) 0.262 0.026 10.240 0.000

V20T8 (lm20) 0.382 0.019 20.669 0.000

Covariances:

Estimate Std.Err z-value P(>|z|)

G1 ~~

G3 0.908 0.020 45.723 0.000

G7 0.898 0.035 25.311 0.000

G8 1.042 0.089 11.745 0.000

G3 ~~

G7 1.275 0.050 25.547 0.000

G8 1.425 0.108 13.251 0.000

G7 ~~

G8 1.945 0.137 14.154 0.000

.V1T1 ~~

.V1T3 0.271 0.028 9.535 0.000

.V1T7 0.277 0.050 5.529 0.000

.V1T8 0.276 0.131 2.101 0.036

.V1T3 ~~

.V1T7 0.350 0.043 8.164 0.000

.V1T8 0.365 0.101 3.597 0.000

.V1T7 ~~

.V1T8 0.460 0.101 4.550 0.000

.V2T1 ~~

.V2T3 0.485 0.021 23.556 0.000

.V2T7 0.533 0.036 14.956 0.000

.V2T8 0.454 0.087 5.209 0.000

.V2T3 ~~

.V2T7 0.547 0.029 18.828 0.000

.V2T8 0.486 0.070 6.987 0.000

.V2T7 ~~

.V2T8 0.547 0.074 7.447 0.000

.V3T1 ~~

.V3T3 0.674 0.022 30.413 0.000

.V3T7 0.621 0.037 16.572 0.000

.V3T8 0.658 0.098 6.704 0.000

.V3T3 ~~

.V3T7 0.574 0.035 16.436 0.000

.V3T8 0.472 0.103 4.595 0.000

.V3T7 ~~

.V3T8 0.649 0.074 8.814 0.000

.V4T1 ~~

.V4T3 0.415 0.023 18.242 0.000

.V4T7 0.244 0.040 6.114 0.000

.V4T8 0.131 0.106 1.233 0.218

.V4T3 ~~

.V4T7 0.434 0.034 12.849 0.000

.V4T8 0.267 0.087 3.059 0.002

.V4T7 ~~

.V4T8 0.115 0.082 1.410 0.158

.V5T1 ~~

.V5T3 0.483 0.027 17.893 0.000

.V5T7 0.493 0.052 9.553 0.000

.V5T8 0.435 0.130 3.337 0.001

.V5T3 ~~

.V5T7 0.369 0.047 7.859 0.000

.V5T8 0.368 0.121 3.049 0.002

.V5T7 ~~

.V5T8 0.389 0.106 3.682 0.000

.V6T1 ~~

.V6T3 0.714 0.015 48.693 0.000

.V6T7 0.653 0.028 23.556 0.000

.V6T8 0.735 0.053 13.748 0.000

.V6T3 ~~

.V6T7 0.686 0.023 29.300 0.000

.V6T8 0.732 0.048 15.263 0.000

.V6T7 ~~

.V6T8 0.686 0.047 14.720 0.000

.V7T1 ~~

.V7T3 0.603 0.024 25.451 0.000

.V7T7 0.764 0.041 18.480 0.000

.V7T8 0.834 0.088 9.437 0.000

.V7T3 ~~

.V7T7 0.452 0.042 10.795 0.000

.V7T8 0.376 0.099 3.778 0.000

.V7T7 ~~

.V7T8 0.501 0.091 5.518 0.000

.V8T1 ~~

.V8T3 0.594 0.019 31.219 0.000

.V8T7 0.493 0.034 14.706 0.000

.V8T8 0.453 0.076 5.940 0.000

.V8T3 ~~

.V8T7 0.458 0.028 16.125 0.000

.V8T8 0.389 0.069 5.621 0.000

.V8T7 ~~

.V8T8 0.463 0.060 7.695 0.000

.V9T1 ~~

.V9T3 0.830 0.017 47.491 0.000

.V9T7 0.811 0.033 24.840 0.000

.V9T8 0.386 0.083 4.674 0.000

.V9T3 ~~

.V9T7 0.746 0.026 28.404 0.000

.V9T8 0.663 0.061 10.955 0.000

.V9T7 ~~

.V9T8 0.791 0.067 11.849 0.000

.V10T1 ~~

.V10T3 0.560 0.018 30.729 0.000

.V10T7 0.453 0.033 13.821 0.000

.V10T8 0.303 0.086 3.536 0.000

.V10T3 ~~

.V10T7 0.583 0.026 22.507 0.000

.V10T8 0.346 0.077 4.473 0.000

.V10T7 ~~

.V10T8 0.489 0.065 7.491 0.000

.V11T1 ~~

.V11T3 0.414 0.019 21.996 0.000

.V11T7 0.317 0.031 10.274 0.000

.V11T8 0.182 0.075 2.429 0.015

.V11T3 ~~

.V11T7 0.460 0.025 18.355 0.000

.V11T8 0.327 0.063 5.219 0.000

.V11T7 ~~

.V11T8 0.257 0.061 4.210 0.000

.V12T1 ~~

.V12T3 0.661 0.017 38.015 0.000

.V12T7 0.539 0.032 17.095 0.000

.V12T8 0.554 0.075 7.418 0.000

.V12T3 ~~

.V12T7 0.614 0.029 21.345 0.000

.V12T8 0.602 0.072 8.370 0.000

.V12T7 ~~

.V12T8 0.499 0.068 7.355 0.000

.V13T1 ~~

.V13T3 0.635 0.017 36.358 0.000

.V13T7 0.572 0.032 17.640 0.000

.V13T8 0.478 0.066 7.201 0.000

.V13T3 ~~

.V13T7 0.663 0.027 24.614 0.000

.V13T8 0.440 0.071 6.181 0.000

.V13T7 ~~

.V13T8 0.586 0.066 8.923 0.000

.V14T1 ~~

.V14T3 0.798 0.012 69.164 0.000

.V14T7 0.749 0.021 35.419 0.000

.V14T8 0.749 0.048 15.729 0.000

.V14T3 ~~

.V14T7 0.793 0.017 46.396 0.000

.V14T8 0.672 0.044 15.111 0.000

.V14T7 ~~

.V14T8 0.784 0.039 19.994 0.000

.V15T1 ~~

.V15T3 0.718 0.016 44.692 0.000

.V15T7 0.626 0.029 21.664 0.000

.V15T8 0.490 0.070 7.011 0.000

.V15T3 ~~

.V15T7 0.630 0.025 25.534 0.000

.V15T8 0.550 0.068 8.101 0.000

.V15T7 ~~

.V15T8 0.663 0.053 12.537 0.000

.V16T1 ~~

.V16T3 0.565 0.015 38.139 0.000

.V16T7 0.467 0.025 18.400 0.000

.V16T8 0.542 0.053 10.306 0.000

.V16T3 ~~

.V16T7 0.537 0.022 24.240 0.000

.V16T8 0.532 0.048 10.995 0.000

.V16T7 ~~

.V16T8 0.563 0.041 13.786 0.000

.V17T1 ~~

.V17T3 0.588 0.018 32.790 0.000

.V17T7 0.479 0.032 14.760 0.000

.V17T8 0.559 0.069 8.070 0.000

.V17T3 ~~

.V17T7 0.455 0.029 15.904 0.000

.V17T8 0.470 0.062 7.584 0.000

.V17T7 ~~

.V17T8 0.480 0.061 7.819 0.000

.V18T1 ~~

.V18T3 0.722 0.016 44.019 0.000

.V18T7 0.678 0.029 23.692 0.000

.V18T8 0.629 0.066 9.564 0.000

.V18T3 ~~

.V18T7 0.713 0.027 26.779 0.000

.V18T8 0.697 0.063 11.003 0.000

.V18T7 ~~

.V18T8 0.732 0.059 12.490 0.000

.V19T1 ~~

.V19T3 0.758 0.019 39.142 0.000

.V19T7 0.556 0.039 14.245 0.000

.V19T8 0.406 0.091 4.481 0.000

.V19T3 ~~

.V19T7 0.725 0.031 23.505 0.000

.V19T8 0.621 0.078 7.934 0.000

.V19T7 ~~

.V19T8 0.768 0.052 14.704 0.000

.V20T1 ~~

.V20T3 0.810 0.009 93.963 0.000

.V20T7 0.728 0.015 47.691 0.000

.V20T8 0.563 0.043 13.001 0.000

.V20T3 ~~

.V20T7 0.780 0.013 61.134 0.000

.V20T8 0.593 0.043 13.889 0.000

.V20T7 ~~

.V20T8 0.660 0.039 17.119 0.000

Intercepts:

Estimate Std.Err z-value P(>|z|)

G1 0.000

G3 -0.549 0.020 -27.291 0.000

G7 -1.247 0.036 -34.187 0.000

G8 -1.427 0.082 -17.501 0.000

.V1T1 0.000

.V2T1 0.000

.V3T1 0.000

.V4T1 0.000

.V5T1 0.000

.V6T1 0.000

.V7T1 0.000

.V8T1 0.000

.V9T1 0.000

.V10T1 0.000

.V11T1 0.000

.V12T1 0.000

.V13T1 0.000

.V14T1 0.000

.V15T1 0.000

.V16T1 0.000

.V17T1 0.000

.V18T1 0.000

.V19T1 0.000

.V20T1 0.000

.V1T3 0.000

.V2T3 0.000

.V3T3 0.000

.V4T3 0.000

.V5T3 0.000

.V6T3 0.000

.V7T3 0.000

.V8T3 0.000

.V9T3 0.000

.V10T3 0.000

.V11T3 0.000

.V12T3 0.000

.V13T3 0.000

.V14T3 0.000

.V15T3 0.000

.V16T3 0.000

.V17T3 0.000

.V18T3 0.000

.V19T3 0.000

.V20T3 0.000

.V1T7 0.000

.V2T7 0.000

.V3T7 0.000

.V4T7 0.000

.V5T7 0.000

.V6T7 0.000

.V7T7 0.000

.V8T7 0.000

.V9T7 0.000

.V10T7 0.000

.V11T7 0.000

.V12T7 0.000

.V13T7 0.000

.V14T7 0.000

.V15T7 0.000

.V16T7 0.000

.V17T7 0.000

.V18T7 0.000

.V19T7 0.000

.V20T7 0.000

.V1T8 0.000

.V2T8 0.000

.V3T8 0.000

.V4T8 0.000

.V5T8 0.000

.V6T8 0.000

.V7T8 0.000

.V8T8 0.000

.V9T8 0.000

.V10T8 0.000

.V11T8 0.000

.V12T8 0.000

.V13T8 0.000

.V14T8 0.000

.V15T8 0.000

.V16T8 0.000

.V17T8 0.000

.V18T8 0.000

.V19T8 0.000

.V20T8 0.000

Thresholds:

Estimate Std.Err z-value P(>|z|)

V1T1|1 (t1_1) -1.304 0.032 -41.112 0.000

V1T1|2 (t1_2) 1.241 0.028 43.645 0.000

V1T1|3 (t1_3) 2.759 0.054 50.877 0.000

V1T3|1 (t1_1) -1.304 0.032 -41.112 0.000

V1T3|2 (t1_2) 1.241 0.028 43.645 0.000

V1T3|3 (t1_3) 2.759 0.054 50.877 0.000

V1T7|1 (t1_1) -1.304 0.032 -41.112 0.000

V1T7|2 (t1_2) 1.241 0.028 43.645 0.000

V1T7|3 (t1_3) 2.759 0.054 50.877 0.000

V1T8|1 (t1_1) -1.304 0.032 -41.112 0.000

V1T8|2 (t1_2) 1.241 0.028 43.645 0.000

V1T8|3 (t1_3) 2.759 0.054 50.877 0.000

V2T1|1 (t2_1) -1.775 0.035 -50.950 0.000

V2T1|2 (t2_2) 0.373 0.025 15.093 0.000

V2T1|3 (t2_3) 1.705 0.033 51.940 0.000

V2T3|1 (t2_1) -1.775 0.035 -50.950 0.000

V2T3|2 (t2_2) 0.373 0.025 15.093 0.000

V2T3|3 (t2_3) 1.705 0.033 51.940 0.000

V2T7|1 (t2_1) -1.775 0.035 -50.950 0.000

V2T7|2 (t2_2) 0.373 0.025 15.093 0.000

V2T7|3 (t2_3) 1.705 0.033 51.940 0.000

V2T8|1 (t2_1) -1.775 0.035 -50.950 0.000

V2T8|2 (t2_2) 0.373 0.025 15.093 0.000

V2T8|3 (t2_3) 1.705 0.033 51.940 0.000

V3T1|1 (t3_1) -1.764 0.037 -47.289 0.000

V3T1|2 (t3_2) 1.020 0.030 34.307 0.000

V3T1|3 (t3_3) 2.007 0.040 49.926 0.000

V3T3|1 (t3_1) -1.764 0.037 -47.289 0.000

V3T3|2 (t3_2) 1.020 0.030 34.307 0.000

V3T3|3 (t3_3) 2.007 0.040 49.926 0.000

V3T7|1 (t3_1) -1.764 0.037 -47.289 0.000

V3T7|2 (t3_2) 1.020 0.030 34.307 0.000

V3T7|3 (t3_3) 2.007 0.040 49.926 0.000

V3T8|1 (t3_1) -1.764 0.037 -47.289 0.000

V3T8|2 (t3_2) 1.020 0.030 34.307 0.000

V3T8|3 (t3_3) 2.007 0.040 49.926 0.000

V4T1|1 (t4_1) -1.677 0.033 -50.817 0.000

V4T1|2 (t4_2) 0.427 0.024 17.770 0.000

V4T1|3 (t4_3) 2.135 0.039 54.858 0.000

V4T3|1 (t4_1) -1.677 0.033 -50.817 0.000

V4T3|2 (t4_2) 0.427 0.024 17.770 0.000

V4T3|3 (t4_3) 2.135 0.039 54.858 0.000

V4T7|1 (t4_1) -1.677 0.033 -50.817 0.000

V4T7|2 (t4_2) 0.427 0.024 17.770 0.000

V4T7|3 (t4_3) 2.135 0.039 54.858 0.000

V4T8|1 (t4_1) -1.677 0.033 -50.817 0.000

V4T8|2 (t4_2) 0.427 0.024 17.770 0.000

V4T8|3 (t4_3) 2.135 0.039 54.858 0.000

V5T1|1 (t5_1) -1.632 0.038 -43.012 0.000

V5T1|2 (t5_2) 0.521 0.028 18.305 0.000

V5T1|3 (t5_3) 2.021 0.042 47.983 0.000

V5T3|1 (t5_1) -1.632 0.038 -43.012 0.000

V5T3|2 (t5_2) 0.521 0.028 18.305 0.000

V5T3|3 (t5_3) 2.021 0.042 47.983 0.000

V5T7|1 (t5_1) -1.632 0.038 -43.012 0.000

V5T7|2 (t5_2) 0.521 0.028 18.305 0.000

V5T7|3 (t5_3) 2.021 0.042 47.983 0.000

V5T8|1 (t5_1) -1.632 0.038 -43.012 0.000

V5T8|2 (t5_2) 0.521 0.028 18.305 0.000

V5T8|3 (t5_3) 2.021 0.042 47.983 0.000

V6T1|1 (t6_1) -0.140 0.023 -6.029 0.000

V6T1|2 (t6_2) 0.841 0.024 34.987 0.000

V6T1|3 (t6_3) 1.625 0.032 50.794 0.000

V6T3|1 (t6_1) -0.140 0.023 -6.029 0.000

V6T3|2 (t6_2) 0.841 0.024 34.987 0.000

V6T3|3 (t6_3) 1.625 0.032 50.794 0.000

V6T7|1 (t6_1) -0.140 0.023 -6.029 0.000

V6T7|2 (t6_2) 0.841 0.024 34.987 0.000

V6T7|3 (t6_3) 1.625 0.032 50.794 0.000

V6T8|1 (t6_1) -0.140 0.023 -6.029 0.000

V6T8|2 (t6_2) 0.841 0.024 34.987 0.000

V6T8|3 (t6_3) 1.625 0.032 50.794 0.000

V7T1|1 (t7_1) -1.888 0.041 -46.460 0.000

V7T1|2 (t7_2) 1.083 0.031 34.499 0.000

V7T1|3 (t7_3) 2.453 0.053 45.910 0.000

V7T3|1 (t7_1) -1.888 0.041 -46.460 0.000

V7T3|2 (t7_2) 1.083 0.031 34.499 0.000

V7T3|3 (t7_3) 2.453 0.053 45.910 0.000

V7T7|1 (t7_1) -1.888 0.041 -46.460 0.000

V7T7|2 (t7_2) 1.083 0.031 34.499 0.000

V7T7|3 (t7_3) 2.453 0.053 45.910 0.000

V7T8|1 (t7_1) -1.888 0.041 -46.460 0.000

V7T8|2 (t7_2) 1.083 0.031 34.499 0.000

V7T8|3 (t7_3) 2.453 0.053 45.910 0.000

V8T1|1 (t8_1) -2.084 0.037 -56.466 0.000

V8T1|2 (t8_2) -0.328 0.024 -13.672 0.000

V8T1|3 (t8_3) 0.691 0.025 27.875 0.000

V8T3|1 (t8_1) -2.084 0.037 -56.466 0.000

V8T3|2 (t8_2) -0.328 0.024 -13.672 0.000

V8T3|3 (t8_3) 0.691 0.025 27.875 0.000

V8T7|1 (t8_1) -2.084 0.037 -56.466 0.000

V8T7|2 (t8_2) -0.328 0.024 -13.672 0.000

V8T7|3 (t8_3) 0.691 0.025 27.875 0.000

V8T8|1 (t8_1) -2.084 0.037 -56.466 0.000

V8T8|2 (t8_2) -0.328 0.024 -13.672 0.000

V8T8|3 (t8_3) 0.691 0.025 27.875 0.000

V9T1|1 (t9_1) -0.404 0.026 -15.746 0.000

V9T1|2 (t9_2) 2.500 0.058 42.882 0.000

V9T1|3 (t9_3) 3.173 0.106 30.030 0.000

V9T3|1 (t9_1) -0.404 0.026 -15.746 0.000

V9T3|2 (t9_2) 2.500 0.058 42.882 0.000

V9T3|3 (t9_3) 3.173 0.106 30.030 0.000

V9T7|1 (t9_1) -0.404 0.026 -15.746 0.000

V9T7|2 (t9_2) 2.500 0.058 42.882 0.000

V9T7|3 (t9_3) 3.173 0.106 30.030 0.000

V9T8|1 (t9_1) -0.404 0.026 -15.746 0.000

V9T8|2 (t9_2) 2.500 0.058 42.882 0.000

V10T1| (t10_1) -0.309 0.021 -14.455 0.000

V10T1| (t10_2) 0.069 0.021 3.286 0.001

V10T1| (t10_3) 1.604 0.029 55.901 0.000

V10T3| (t10_1) -0.309 0.021 -14.455 0.000

V10T3| (t10_2) 0.069 0.021 3.286 0.001

V10T3| (t10_3) 1.604 0.029 55.901 0.000

V10T7| (t10_1) -0.309 0.021 -14.455 0.000

V10T7| (t10_2) 0.069 0.021 3.286 0.001

V10T7| (t10_3) 1.604 0.029 55.901 0.000

V10T8| (t10_1) -0.309 0.021 -14.455 0.000

V10T8| (t10_2) 0.069 0.021 3.286 0.001

V10T8| (t10_3) 1.604 0.029 55.901 0.000

V11T1| (t11_1) -1.151 0.026 -44.955 0.000

V11T1| (t11_2) 0.619 0.021 28.831 0.000

V11T1| (t11_3) 1.786 0.034 52.965 0.000

V11T3| (t11_1) -1.151 0.026 -44.955 0.000

V11T3| (t11_2) 0.619 0.021 28.831 0.000

V11T3| (t11_3) 1.786 0.034 52.965 0.000

V11T7| (t11_1) -1.151 0.026 -44.955 0.000

V11T7| (t11_2) 0.619 0.021 28.831 0.000

V11T7| (t11_3) 1.786 0.034 52.965 0.000

V11T8| (t11_1) -1.151 0.026 -44.955 0.000

V11T8| (t11_2) 0.619 0.021 28.831 0.000

V11T8| (t11_3) 1.786 0.034 52.965 0.000

V12T1| (t12_1) -0.989 0.027 -36.313 0.000

V12T1| (t12_2) 0.913 0.025 36.285 0.000

V12T1| (t12_3) 2.547 0.055 46.626 0.000

V12T3| (t12_1) -0.989 0.027 -36.313 0.000

V12T3| (t12_2) 0.913 0.025 36.285 0.000

V12T3| (t12_3) 2.547 0.055 46.626 0.000

V12T7| (t12_1) -0.989 0.027 -36.313 0.000

V12T7| (t12_2) 0.913 0.025 36.285 0.000

V12T7| (t12_3) 2.547 0.055 46.626 0.000

V12T8| (t12_1) -0.989 0.027 -36.313 0.000

V12T8| (t12_2) 0.913 0.025 36.285 0.000

V12T8| (t12_3) 2.547 0.055 46.626 0.000

V13T1| (t13_1) -1.515 0.034 -44.014 0.000

V13T1| (t13_2) 0.153 0.023 6.575 0.000

V13T1| (t13_3) 2.571 0.053 48.173 0.000

V13T3| (t13_1) -1.515 0.034 -44.014 0.000

V13T3| (t13_2) 0.153 0.023 6.575 0.000

V13T3| (t13_3) 2.571 0.053 48.173 0.000

V13T7| (t13_1) -1.515 0.034 -44.014 0.000

V13T7| (t13_2) 0.153 0.023 6.575 0.000

V13T7| (t13_3) 2.571 0.053 48.173 0.000

V13T8| (t13_1) -1.515 0.034 -44.014 0.000

V13T8| (t13_2) 0.153 0.023 6.575 0.000

V13T8| (t13_3) 2.571 0.053 48.173 0.000

V14T1| (t14_1) -0.915 0.028 -32.829 0.000

V14T1| (t14_2) 0.471 0.023 20.412 0.000

V14T1| (t14_3) 1.114 0.027 41.402 0.000

V14T3| (t14_1) -0.915 0.028 -32.829 0.000

V14T3| (t14_2) 0.471 0.023 20.412 0.000

V14T3| (t14_3) 1.114 0.027 41.402 0.000

V14T7| (t14_1) -0.915 0.028 -32.829 0.000

V14T7| (t14_2) 0.471 0.023 20.412 0.000

V14T7| (t14_3) 1.114 0.027 41.402 0.000

V14T8| (t14_1) -0.915 0.028 -32.829 0.000

V14T8| (t14_2) 0.471 0.023 20.412 0.000

V14T8| (t14_3) 1.114 0.027 41.402 0.000

V15T1| (t15_1) -1.620 0.034 -47.419 0.000

V15T1| (t15_2) 0.170 0.024 7.159 0.000

V15T1| (t15_3) 1.834 0.037 49.165 0.000

V15T3| (t15_1) -1.620 0.034 -47.419 0.000

V15T3| (t15_2) 0.170 0.024 7.159 0.000

V15T3| (t15_3) 1.834 0.037 49.165 0.000

V15T7| (t15_1) -1.620 0.034 -47.419 0.000

V15T7| (t15_2) 0.170 0.024 7.159 0.000

V15T7| (t15_3) 1.834 0.037 49.165 0.000

V15T8| (t15_1) -1.620 0.034 -47.419 0.000

V15T8| (t15_2) 0.170 0.024 7.159 0.000

V15T8| (t15_3) 1.834 0.037 49.165 0.000

V16T1| (t16_1) -0.997 0.025 -40.613 0.000

V16T1| (t16_2) 0.684 0.022 31.535 0.000

V16T1| (t16_3) 1.263 0.026 48.767 0.000

V16T3| (t16_1) -0.997 0.025 -40.613 0.000

V16T3| (t16_2) 0.684 0.022 31.535 0.000

V16T3| (t16_3) 1.263 0.026 48.767 0.000

V16T7| (t16_1) -0.997 0.025 -40.613 0.000

V16T7| (t16_2) 0.684 0.022 31.535 0.000

V16T7| (t16_3) 1.263 0.026 48.767 0.000

V16T8| (t16_1) -0.997 0.025 -40.613 0.000

V16T8| (t16_2) 0.684 0.022 31.535 0.000

V16T8| (t16_3) 1.263 0.026 48.767 0.000

V17T1| (t17_1) -1.604 0.032 -50.062 0.000

V17T1| (t17_2) 0.325 0.023 13.941 0.000

V17T1| (t17_3) 2.074 0.039 52.676 0.000

V17T3| (t17_1) -1.604 0.032 -50.062 0.000

V17T3| (t17_2) 0.325 0.023 13.941 0.000

V17T3| (t17_3) 2.074 0.039 52.676 0.000

V17T7| (t17_1) -1.604 0.032 -50.062 0.000

V17T7| (t17_2) 0.325 0.023 13.941 0.000

V17T7| (t17_3) 2.074 0.039 52.676 0.000

V17T8| (t17_1) -1.604 0.032 -50.062 0.000

V17T8| (t17_2) 0.325 0.023 13.941 0.000

V17T8| (t17_3) 2.074 0.039 52.676 0.000

V18T1| (t18_1) 0.355 0.021 16.601 0.000

V18T1| (t18_2) 1.502 0.028 53.235 0.000

V18T1| (t18_3) 2.271 0.048 46.877 0.000

V18T3| (t18_1) 0.355 0.021 16.601 0.000

V18T3| (t18_2) 1.502 0.028 53.235 0.000

V18T3| (t18_3) 2.271 0.048 46.877 0.000

V18T7| (t18_1) 0.355 0.021 16.601 0.000

V18T7| (t18_2) 1.502 0.028 53.235 0.000

V18T7| (t18_3) 2.271 0.048 46.877 0.000

V18T8| (t18_1) 0.355 0.021 16.601 0.000

V18T8| (t18_2) 1.502 0.028 53.235 0.000

V18T8| (t18_3) 2.271 0.048 46.877 0.000

V19T1| (t19_1) 1.010 0.022 45.468 0.000

V19T1| (t19_2) 1.561 0.029 54.246 0.000

V19T1| (t19_3) 1.923 0.038 50.608 0.000

V19T3| (t19_1) 1.010 0.022 45.468 0.000

V19T3| (t19_2) 1.561 0.029 54.246 0.000

V19T3| (t19_3) 1.923 0.038 50.608 0.000

V19T7| (t19_1) 1.010 0.022 45.468 0.000

V19T7| (t19_2) 1.561 0.029 54.246 0.000

V19T7| (t19_3) 1.923 0.038 50.608 0.000

V19T8| (t19_1) 1.010 0.022 45.468 0.000

V19T8| (t19_2) 1.561 0.029 54.246 0.000

V19T8| (t19_3) 1.923 0.038 50.608 0.000

V20T1| (t20_1) -0.698 0.023 -30.052 0.000

V20T1| (t20_2) 0.298 0.021 14.168 0.000

V20T1| (t20_3) 1.151 0.027 42.890 0.000

V20T3| (t20_1) -0.698 0.023 -30.052 0.000

V20T3| (t20_2) 0.298 0.021 14.168 0.000

V20T3| (t20_3) 1.151 0.027 42.890 0.000

V20T7| (t20_1) -0.698 0.023 -30.052 0.000

V20T7| (t20_2) 0.298 0.021 14.168 0.000

V20T7| (t20_3) 1.151 0.027 42.890 0.000

V20T8| (t20_1) -0.698 0.023 -30.052 0.000

V20T8| (t20_2) 0.298 0.021 14.168 0.000

V20T8| (t20_3) 1.151 0.027 42.890 0.000

Variances:

Estimate Std.Err z-value P(>|z|)

G1 1.000

G3 1.376 0.040 34.034 0.000

G7 2.053 0.084 24.522 0.000

G8 2.716 0.223 12.160 0.000

.V1T1 1.000

.V2T1 1.000

.V3T1 1.000

.V4T1 1.000

.V5T1 1.000

.V6T1 1.000

.V7T1 1.000

.V8T1 1.000

.V9T1 1.000

.V10T1 1.000

.V11T1 1.000

.V12T1 1.000

.V13T1 1.000

.V14T1 1.000

.V15T1 1.000

.V16T1 1.000

.V17T1 1.000

.V18T1 1.000

.V19T1 1.000

.V20T1 1.000

.V1T3 1.000

.V2T3 1.000

.V3T3 1.000

.V4T3 1.000

.V5T3 1.000

.V6T3 1.000

.V7T3 1.000

.V8T3 1.000

.V9T3 1.000

.V10T3 1.000

.V11T3 1.000

.V12T3 1.000

.V13T3 1.000

.V14T3 1.000

.V15T3 1.000

.V16T3 1.000

.V17T3 1.000

.V18T3 1.000

.V19T3 1.000

.V20T3 1.000

.V1T7 1.000

.V2T7 1.000

.V3T7 1.000

.V4T7 1.000

.V5T7 1.000

.V6T7 1.000

.V7T7 1.000

.V8T7 1.000

.V9T7 1.000

.V10T7 1.000

.V11T7 1.000

.V12T7 1.000

.V13T7 1.000

.V14T7 1.000

.V15T7 1.000

.V16T7 1.000

.V17T7 1.000

.V18T7 1.000

.V19T7 1.000

.V20T7 1.000

.V1T8 1.000

.V2T8 1.000

.V3T8 1.000

.V4T8 1.000

.V5T8 1.000

.V6T8 1.000

.V7T8 1.000

.V8T8 1.000

.V9T8 1.000

.V10T8 1.000

.V11T8 1.000

.V12T8 1.000

.V13T8 1.000

.V14T8 1.000

.V15T8 1.000

.V16T8 1.000

.V17T8 1.000

.V18T8 1.000

.V19T8 1.000

.V20T8 1.000

Scales y*:

Estimate Std.Err z-value P(>|z|)

V1T1 0.738

V2T1 0.752

V3T1 0.703

V4T1 0.750

V5T1 0.646

V6T1 0.855

V7T1 0.690

V8T1 0.781

V9T1 0.838

V10T1 0.900

V11T1 0.849

V12T1 0.805

V13T1 0.799

V14T1 0.847

V15T1 0.782

V16T1 0.915

V17T1 0.791

V18T1 0.894

V19T1 0.967

V20T1 0.934

V1T3 0.682

V2T3 0.698

V3T3 0.645

V4T3 0.695

V5T3 0.585

V6T3 0.814

V7T3 0.631

V8T3 0.730

V9T3 0.795

V10T3 0.869

V11T3 0.807

V12T3 0.756

V13T3 0.749

V14T3 0.805

V15T3 0.731

V16T3 0.888

V17T3 0.740

V18T3 0.862

V19T3 0.956

V20T3 0.912

V1T7 0.606

V2T7 0.623

V3T7 0.568

V4T7 0.620

V5T7 0.508

V6T7 0.754

V7T7 0.554

V8T7 0.658

V9T7 0.731

V10T7 0.821

V11T7 0.746

V12T7 0.687

V13T7 0.679

V14T7 0.744

V15T7 0.659

V16T7 0.845

V17T7 0.670

V18T7 0.812

V19T7 0.936

V20T7 0.877

V1T8 0.553

V2T8 0.570

V3T8 0.515

V4T8 0.567

V5T8 0.457

V6T8 0.707

V7T8 0.501

V8T8 0.605

V9T8 0.682

V10T8 0.781

V11T8 0.698

V12T8 0.635

V13T8 0.627

V14T8 0.695

V15T8 0.606

V16T8 0.808

V17T8 0.617

V18T8 0.770

V19T8 0.918

V20T8 0.846
